# Climate suitability of the Mediterranean Basin for citrus black spot disease (*Phyllosticta citricarpa*) based on a generic infection model

**DOI:** 10.1038/s41598-022-22775-z

**Published:** 2022-11-18

**Authors:** Anaïs Galvañ, Naima Boughalleb-M’Hamdi, Najwa Benfradj, Sabrine Mannai, Elena Lázaro, Antonio Vicent

**Affiliations:** 1 Institut Valencià d’Investigacions Agràries (IVIA), Centre de Protecció Vegetal i Biotecnologia, 46113 Moncada, Valencia Spain; 2 Department of Biological Sciences and Plant Protection, Institut Supérieur Agronomique de Chott Mariem, LR21AGR05, University of Sousse, Chott Mariem, Sousse, 4042 Tunisia

**Keywords:** Plant sciences, Plant ecology

## Abstract

Citrus black spot (CBS), caused by the fungus *Phyllosticta citricarpa*, is associated with serious yield and quality losses. The climate suitability of the Mediterranean Basin for CBS development has been long debated. However, CBS has been described in Tunisia. In this study, a generic model was used to simulate potential infections by ascospores and pycnidiospores together with a degree-day model to predict the onset of ascospore release. High-resolution climatic data were retrieved from the ERA5-Land dataset for the citrus-growing regions in the Mediterranean Basin and other locations where CBS is present. In general, the onset of ascospore release was predicted to occur late in spring, but there is no agreement on the adequacy of this empirical model for extrapolation to the Mediterranean Basin. The generic model indicated that infections by ascospores and pycnidiospores would be concentrated mainly in autumn, as well as in spring for pycnidiospores. In contrast to previous studies, the percentage of hours suitable for infection was higher for pycnidiospores than for ascospores. The values obtained with the generic infection model for Tunisia and several CBS-affected locations worldwide were similar to those for other citrus-growing regions in Europe and Northern Africa. These results support previous work indicating that the climate of the Mediterranean Basin is suitable for CBS development.

## Introduction

Citrus black spot (CBS), caused by the fungus *Phyllosticta citricarpa* (McAlpine) Van der Aa (Synonym: *Guignardia citricarpa* Kiely), affects most cultivated *Citrus* species, thereby inducing serious economic losses^1^. Symptoms of the disease consist of different types of blemishes on the fruit rind. Citrus black spot lesions do not affect the edible part of the fruit, but their presence on the peel makes them unsuitable for the fresh market. The disease also induces premature fruit drop, resulting in serious yield losses^2^. Less often, lesions caused by *P. citricarpa* can also be present in twigs and leaves, mainly in highly susceptible hosts such as lemon (*Citrus limon* (L.) Burm.f.)^1^.

The species *P. citricarpa* is heterothallic and requires the combination of two complementary mating types to reproduce sexually^3^. Ascospores are sexually produced in the leaf litter and released into the orchard air once they are mature. Airborne ascospores disseminate the pathogen over relatively long distances and are considered the primary inoculum source in some areas^4,5^. *P. citricarpa* also reproduces asexually to produce conidia (pycnidiospores) in pycnidia that are formed in lesions in fruit, leaves and twigs as well as in the leaf litter^6^. Pycnidiospores are produced in slimy masses and disseminated over relatively short distances by rain splash, although their potential for dispersal can be greater with wind-driven rains^7^. Pycnidiospores were deemed not to be epidemiologically important^1^, but recent studies demonstrate that they play a major role in certain epidemiological settings^6,8–12^.

Ascospores and pycnidiospores of *P. citricarpa* germinate in the presence of adequate temperatures and wetness, then form appressoria which infect the host plant tissues. Nevertheless, the effects of different temperatures and wetness durations in *P. citricarpa* infection have been not quantified and only in vitro studies on spore germination and appressorium formation are available^13–15^. Citrus leaves are susceptible for about 8-10 months after they emerge^16^. The critical period for fruit infection starts at fruit set and lasts 4–7 months^17^. The disease is characterized by a long incubation period and symptoms appear 2–5 months after infection. Symptoms are expressed at the ripening stage and even later during postharvest. The disease has a long lag phase and, depending on the climate and host susceptibility, several years may elapse between pathogen introduction and inoculum build-up and epidemic onset^18^.

The disease is presently causing crop losses in citrus-growing regions in America, Asia, Africa and Australia^19^. In the Mediterranean Basin, Guarnaccia et al.^20^ cited the finding of *P. citricarpa* in leaf litter in Italy, Malta and Portugal and its sister species, *P. paracitricarpa* Guarnaccia & Crous, in Greece. However, those reports were not confirmed in the official surveys conducted by the competent National Plant Protection Organizations. Recently, CBS has been described in the main citrus-growing region in Tunisia^21^.

Due to its potential negative impact on citrus production, a number of citrus-growing countries worldwide consider *P. citricarpa* a quarantine pathogen and it has been recommended for regulation by several Regional Plant Protection Organizations^19,22^. In the Mediterranean Basin, *P. citricarpa* has quarantine status in the European Union (EU), Egypt, Israel, Jordan, Montenegro, Morocco, Tunisia and Turkey^19,22^. According to Regulation (EU) 2019/2072^23^, the introduction of citrus plants into the EU from third countries is prohibited. Importing citrus fruits into the EU from areas where *P. citricarpa* is present is allowed, but they should comply with special requirements including the application of appropriate treatments and official inspections to ensure they are free of symptoms. Furthermore, Regulation (EU) 2019/1702^23^ also establishes *P. citricarpa* as one of the priority quarantine ‘pests’ in the EU, and thus annual surveys are mandatory, together with the implementation of contingency plans, simulation exercises, action plans for eradication and the provision of information to the public.

Humid subtropical climates with rainy summers and mild winters are considered the most favourable for CBS epidemics^1^. It is known that warm temperatures and frequent rains favour the development of *P. citricarpa* pseudothecia, ascospore release, pycnidiospore dispersal and subsequent infections^4,5,7^. Nevertheless, CBS is also present in areas with arid climates characterized by low annual rainfall values^24^. The suitability of Mediterranean climates for CBS development has been widely debated^24–31^. In fact, some of the exclusion measures for *P. citricarpa* established by the EU regulations have been considered unjustified based on the assumption that climates in the Mediterranean Basin are not conducive to CBS^26,27^.

Climate suitability for CBS has been assessed using different modelling methodologies, in some cases resulting in contradictory conclusions particularly in relation to the citrus-growing areas in the EU. The software CLIMEX was used in several studies to estimate the potential geographic range of CBS based on distribution data and biological/epidemiological information^26,27^. Nevertheless, spatially-explicit species distribution models indicated that, in addition to the climate itself, spatial proximity to affected areas is also relevant in the geographic distribution of CBS^25^, and so approaches like CLIMEX that do not take spatial autocorrelation into account could be problematic in this regard.

Climate suitability for CBS was also assessed with process-based models based on temperature and wetness duration requirements for *P. citricarpa* infection. A generic infection model for foliar fungal plant pathogens and other modelling proposals built under non-linear functions were used to simulate *P. citricarpa* pycnidiospore and ascospore infection^28,29,31,32^. In the case of the generic infection model, it was designed primarily for pathogens that do not have extensive biological data^33^, as it is the case of *P. citricarpa*. Empirical degree-day models for *Phyllosticta* spp. ascospore maturation and release were developed to predict the periods of potential ascospore availability^4,5^. Some studies combined those models with the non-linear functions noted above, integrating both ascospore availability and infection^28,31,32^.

The recent report of CBS in Tunisia^21^ opens up a new perspective in relation to the climate suitability of the Mediterranean Basin for CBS development. Previous studies using the models for *P. citricarpa* infection and/or ascospore availability were implemented with climate data from specific locations, thus having a very low spatial coverage^4,5,28,32^. Others used gridded climate data, but only for the EU territory and with a relatively coarse spatial resolution^29,31^. Models for *P. citricarpa* infection were parameterized based on spore germination data and no statistical inference was performed. Moreover, citrus-growing regions in Tunisia were not considered in any of these previous works.

The objective of our study is thus to assess the climate suitability for CBS development by implementing the models for *P. citricarpa* infection, pseudothecium maturation and onset of ascospore release. A dataset with high spatial and temporal resolution was used covering all the citrus production regions in the Mediterranean Basin. Bayesian inference was explored to estimate the parameters of the *P. citricarpa* infection model. Results will help to unravel the epidemiological features that allowed the disease to establish itself and spread under Mediterranean conditions. Likewise, our study will contribute to reducing uncertainty in risk assessment that informs risk management decisions. Following open-science principles, data and code are made available to enhance reproducibility and replicability.

## Results

### Generic infection model

**Figure 1:**
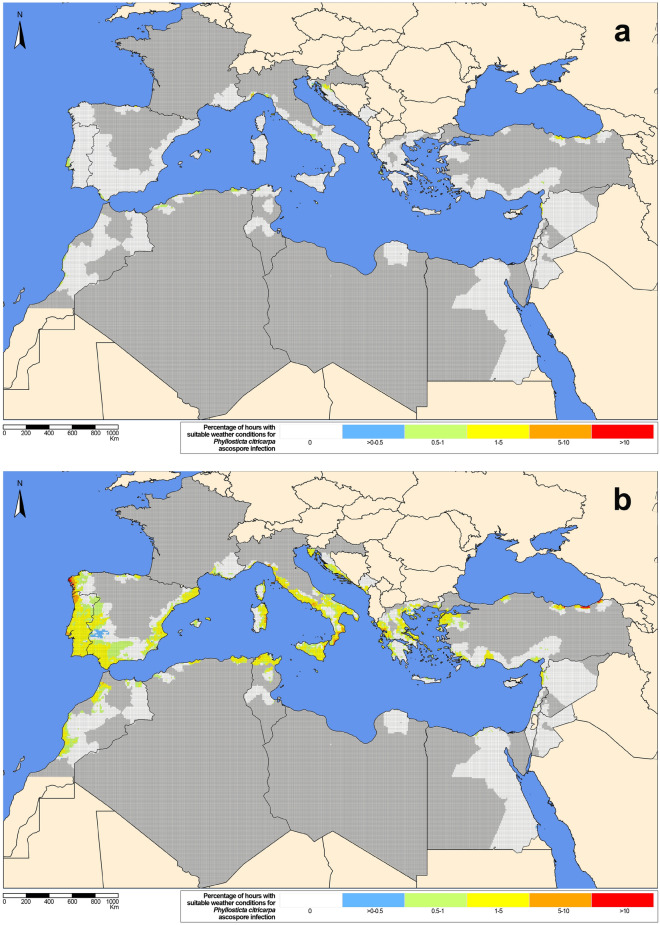
Percentage of hours ($=$ 0 white, ]0–0.5] blue, ]0.5–1] green, ]1–5] yellow, ]5–10] orange, $>10$ red) with suitable weather conditions for *Phyllosticta citricarpa *ascospore infection (generic infection model for foliar fungal pathogens by Magarey et al.^33^, configuration scenario S1) for the 9-km grid interpolated climatic data of the citrus-growing regions in the Mediterranean Basin from 2009 to 2018 for (**a**) spring (March to May) and (**b**) autumn (September to November). Non citrus areas inside citrus-growing countries in dark-grey. The maps were created by the authors using the software R 3.6.0, https://www.R-project.org.

**Figure 2:**
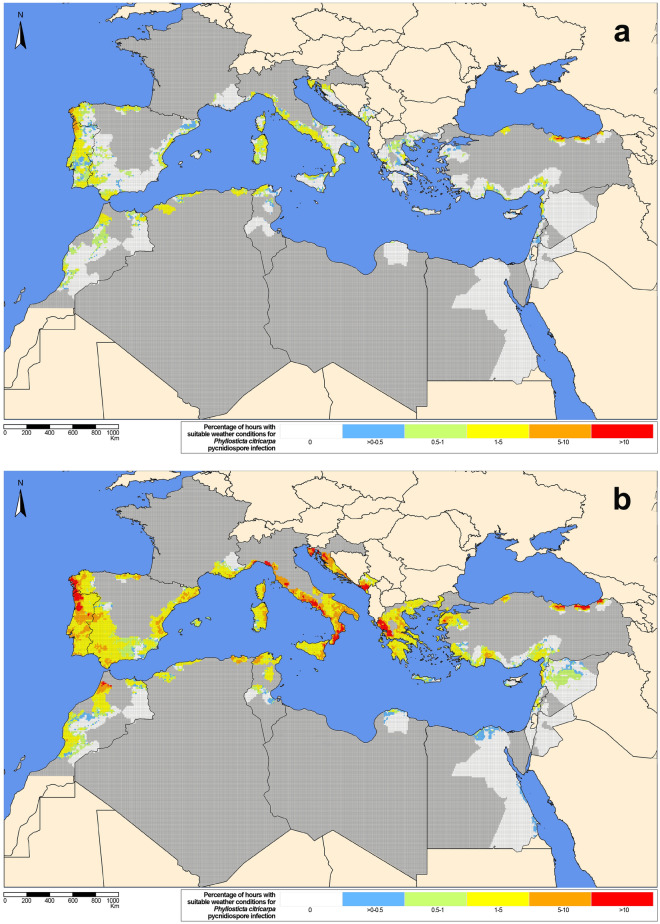
Percentage of hours ($=$ 0 white, ]0–0.5] blue, ]0.5–1] green, ]1–5] yellow, ]5–10] orange, $>10$ red) with suitable weather conditions for *Phyllosticta citricarpa* pycnidiospore infection (generic infection model for foliar fungal pathogens by Magarey et al.^33^, configuration scenario S1) for the 9-km grid interpolated climatic data of the citrus-growing regions in the Mediterranean Basin from 2009 to 2018 for (**a**) spring (March to May) and (**b**) autumn (September to November). Non citrus areas inside citrus-growing countries in dark-grey. The maps were created by the authors using the software R 3.6.0, https://www.R-project.org.

The generic infection model simulated ascospore and/or pycnidiospore infections in all the citrus-growing countries of the Mediterranean Basin, with both configuration scenarios S1 (Figs. 1, 2) and S2 (Supplementary Figs. SA1, SA2). For the overall 10-year study period, the model generally simulated a higher percentage of hours with suitable weather conditions for ascospore and pycnidiospore infection in autumn than in spring, defined as per the meteorological season calendar^34^ (Figs. 1, 2, Supplementary Materials A–D). A few infections were simulated by the model in summer and winter. These results were consistent with both S1 and S2. In general, October and November had the highest simulated values for ascospore and pycnidiospore infections.

The extent of the area and the percentage of hours with suitable weather conditions for ascospore and pycnidiospore infection were higher with S2 (Supplementary Figs. SA1, SA2) than with S1 (Figs. 1, 2). In general, values simulated for ascospores and pycnidiospores were more similar with S2 than with S1. With S1, values simulated for pycnidiospores were in general higher than for ascospores, while with S2, for certain months and countries, the percentage of hours with suitable weather conditions for ascospore infection was higher than for pycnidiospores. However, with S1 Cyprus, Syria, Turkey, Tunisia and Libya had a higher percentage of hours with suitable weather conditions for ascospore infection than for pycnidiospores in winter months (December to February). In France, Greece, Italy and Lebanon this occurred from November to April, from October to May in the Iberian Peninsula and Montenegro, and from January to May in Malta and Morocco (Supplementary Materials B–D).

No favourable climatic conditions for infection were simulated in Jordan for ascospores and pycnidiospores with S1 and ascospores with S2, respectively. No ascospore infections were simulated in spring with S1 (Figs. 1, 2) in Libya and with S2 (Supplementary Figs. SA1, SA2) in Cyprus, Egypt, Israel and Lebanon. With S1, the model simulated a few hours with favourable weather conditions for ascospore infection in spring. Except for Malta and Morocco, the highest values were observed in May (Supplementary Materials B–D). With S2, the model simulated numerous hours with favourable weather conditions for ascospore infection in spring, except for Egypt, Jordan and Libya. In general, the percentage of hours with suitable weather conditions for ascospore infection in spring was higher in coastal than in inland areas, both with S1 and S2. With S1, the percentage of hours with suitable weather conditions for ascospore infection was generally between 0 and 1%, with some peaks about 1–5% and 5–10% in the coastal areas. In S2, values were around 1–5% with some peaks of 5–10% and > 10% also in coastal areas. In autumn, the percentage of hours with suitable weather conditions for ascospore infection simulated with S1 was also higher in coastal than in inland areas (Fig. 1, Supplementary Materials B, C). For instance, the coastal areas of the northwest of Spain, Sardinia and Catania in Italy, and Ordu, Trabzon and Artvin in Turkey had the highest values, with peaks of 5–10% and > 10%. Coastal areas in Trabzon, Turkey, had the maximum value of up to 26%. With S2, most of the areas with high values of ascospore infections in spring also presented high values in autumn. In this case, values ranged between 1–5%, 5–10% and > 10%. The highest values were also simulated in the coastal areas of northwest Spain and Portugal, Italy, Greece and Ordu, Trabzon and Artvin in Turkey, with peaks of > 10% (Supplementary Figs. SA1, SA2).

For pycnidiospores, the highest percentage of hours with suitable weather conditions occurred in October and November, both with S1 and S2 (Fig. 2, Supplementary Material A). In Spring, the highest percentage of hours with suitable weather conditions in both scenarios was obtained in May (Fig. 2, Supplementary Materials A, B). A few infections with pycnidiospores were simulated in summer and winter. In spring, the highest percentage of hours with suitable weather conditions for pycnidiospore infection with S1 occurred in the north-western coastal areas of the Iberian Peninsula and the coastal areas of Turkey (Fig. 2a, Supplementary Material D). In autumn, the highest values were in the north-western coastal areas of the Iberian Peninsula, Croatia and Greece, western coastal areas of Italy, northeast coastal areas of Montenegro and northern coastal areas of Turkey. With S1, the values in spring ranged from 1–5%, whereas in autumn a number of grid cells showed values between 5–10 and > 10%. With S2, the highest values in spring were also observed in the north-western coastal areas of the Iberian Peninsula and northern coastal areas of Turkey (Supplementary Material A). In autumn, with S2, the highest values were observed mainly in western and northeast coastal areas of the Iberian Peninsula, Italy, Croatia, Montenegro, Tunisia, Morocco, Greece and Algeria as well as in some areas of southeast France and Turkey. With S2, the percentage of hours with suitable weather conditions in spring ranged from 1–5%, while in autumn the values were 5–10% and > 10%.

**Figure 3:**
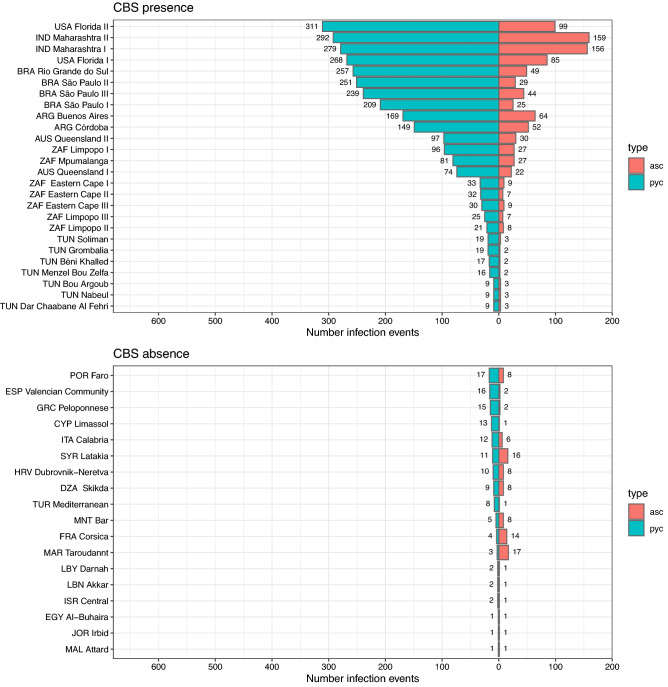
Number of infection events for *Phyllosticta citricarpa* ascospores (asc) and pycnidiospores (pyc) from 2009 to 2018 simulated by the generic infection model for foliar fungal pathogens by Magarey et al.^33^ (configuration scenario S1) for locations where citrus black spot (CBS) is either present or absent. Number of infection events in x-axis and selected locations where CBS is either present or absent in y-axis. Ascospores (asc) in red and pycnidiospores (pyc) in turquoise.

In the selected locations where CBS is present worldwide (Table 2), the generic infection model also simulated a higher number of infection events for pycnidiospores than for ascospores, both with S1 (Fig. 3) and S2 ( Supplementary Fig. SA3). This was also the case with S2 for the selected locations where CBS is absent (Table 2). In contrast, with S1 the model simulated higher numbers of infection events for ascospores than for pycnidiospores in the selected locations where CBS is absent in Morocco, Syria, France, Portugal, Croatia, Algeria and Montenegro. Overall, the model simulated higher values for pycnidiospore infection with S2 than with S1. Nevertheless, in Gayndah (AUS Queensland II), Morocco, Libya, Lebanon, Egypt, Jordan and Malta the model simulated higher values for ascospore infection with S1 than with S2. The model did not simulate any ascospore infection event in Libya, Lebanon, Egypt, Jordan or Malta with S2. For the selected location where CBS is present, with S1 the model simulated the highest number of ascospore (n > 150) and pycnidiospore (n > 300) infection events in locations with warm wet summers (Fig. 3, Table 2), like India (IND Maharashtra I-II) and Florida (USA Florida II). The lowest numbers of ascospore (n $\ge$ 2) and pycnidiospore (n$\ge$9) infection events were simulated in locations with hot dry summers, like Tunisia and South Africa (ZAF Limpopo III, ZAF Eastern Cape I-III). With S1, the highest number of ascospore infection events (n = 159) was in India (IND Maharashtra II) and the lowest (n = 2) in Tunisia (TUN Grombalia, TUN Béni Khalled, TUN Menzel Bou Zelfa). For pycnidiospores, the highest number of infection events (n = 311) was simulated in Florida (USA Florida II) and the lowest (n = 9) in Tunisia (TUN Bou Argoub, TUN Nabeul, TUN Dar Chaabane Al Fehri) (Fig. 3, Table 2).

With S2 (Supplementary Fig. SA3, Table 2), the highest numbers of ascospore (n > 80) and pycnidiospore (n > 600) infection events were also simulated by the model in locations with warm wet summers, like Brazil (BRA São Paulo I, II, III, BRA Rio Grande do Sul), Argentina (ARG Buenos Aires, ARG Córdoba) and India (IND Maharashtra I and II). The lowest numbers of ascospore (n$\ge$4) and pycnidiospore (n$\ge$43) infection events were simulated in locations with hot dry summers like Tunisia (TUN Bou Argoub, TUN Nabeul, TUN Dar Chaabane Al Fehri) and South Africa (ZAF Limpopo III). The highest number of ascospore infection events (n = 82) was in India (IND Maharastra II) and the lowest (n = 4) in Tunisia (TUN Bou Argoub). The highest number of pycnidiospore infection events (n = 610) was simulated in Brazil (BRA São Paulo II) and the lowest (n = 43) in Tunisia (TUN Nabeul and TUN Dar Chaabane Al Fehri).

Considering the selected locations where CBS is absent, all of them in the Mediterranean Basin with hot dry summers, with S1 (Fig. 3, Table 2) the highest number of ascospore infection events (n = 17) was simulated in Morocco (MAR Taroudannt) and the lowest (n = 1) in Cyprus (CYP Limassol), Turkey (TUR Mediterranean), Libya (LBY Darnah), Lebanon (LEB Akkar), Israel (ISR Central), Egypt (EGY Al-Buhaira), Jordan (JOR Irbid) and Malta (MAL Attard). The highest number of pycnidiospore infection events (n = 17) was simulated in Portugal (POR Faro) and the lowest (n = 1) in Egypt (EGY Al-Buhaira), Jordan (JOR Irbid) and Malta (Mal Attard). With S2 (Supplementary Fig. SA3, Table 2), the highest number of ascospore infection events (n = 67) was in Italy (ITA Calabria) and the lowest (n = 0) in Libya (LBY Darnah), Lebanon (LEB Akkar), Egypt (EGY Al-Buhaira), Jordan (JOR Irbid) and Malta (MAL Attard).

### Pseudothecium maturation and onset of ascospore release model

**Figure 4:**
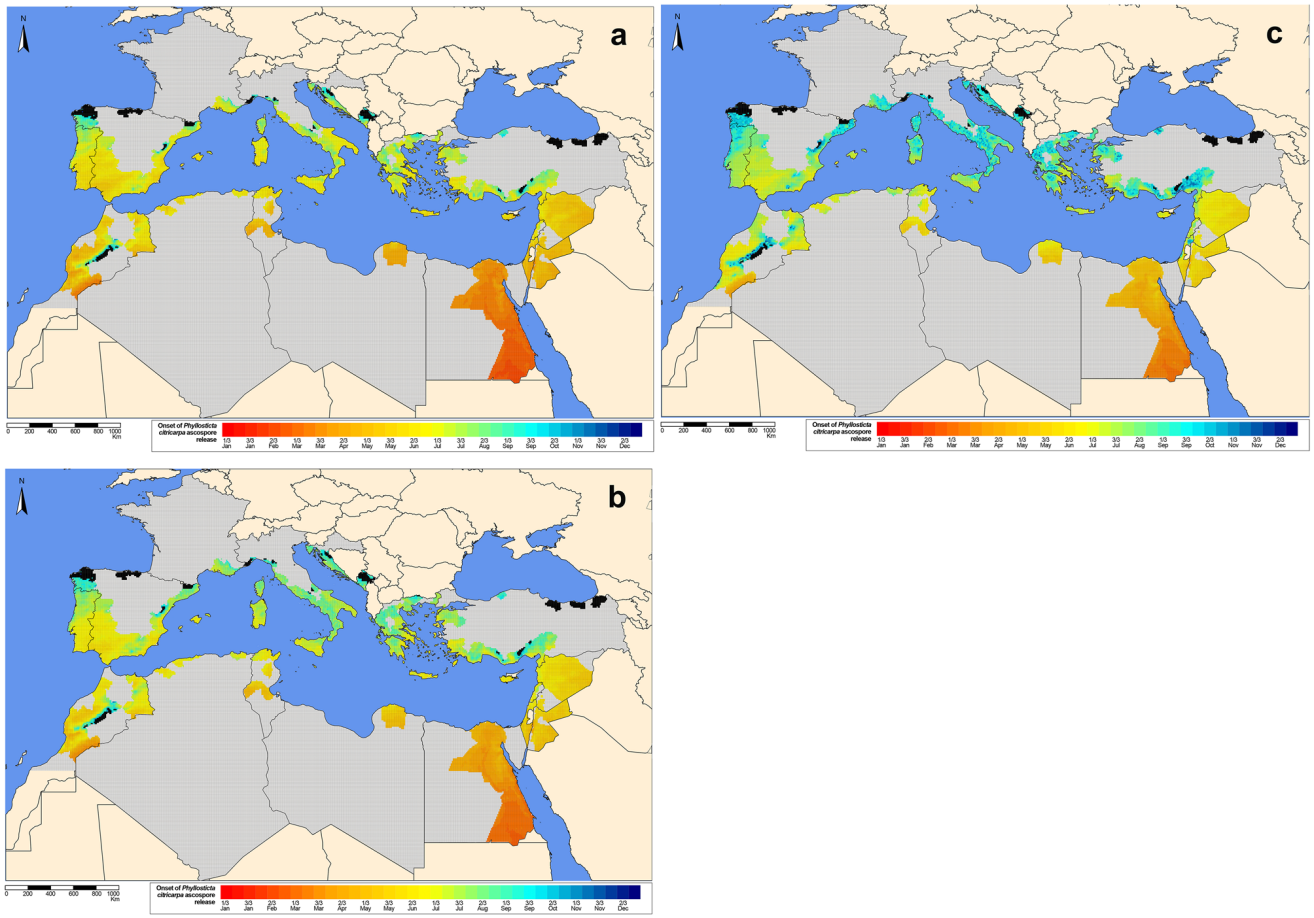
Onset of *Phyllosticta citricarpa* ascospore release predicted by the model by Moyo et al.^4^ with a probability threshold of 0.5 for the 9-km grid interpolated climatic data of the citrus-growing regions in the Mediterranean Basin from 2009 to 2018. (**a**) 5th, (**b**) 50th and (**c**) 95th percentiles. Black cells with probability $<0.5$. Calendar date range aggregated by 1/3 month. Color scale from red (1/3 January) to dark blue (3/3 December). Non citrus regions inside citrus-growing countries in dark-grey. The maps were created by the authors using the software R 3.6.0, https://www.R-project.org.

In general, the model by Moyo et al.^4^ predicted the onset of ascospore release in the study area between March and October, with the earliest release dates in the southern countries of the Mediterranean Basin, particularly in some regions of Egypt, Israel, Jordan and Morocco (Fig. 4, Supplementary Material E). This trend was consistent over the 10-year study period given the relatively small variability observed with the 5th, 50th and 95th percentiles. Nevertheless, a shift in the predictions towards the latest dates was observed as the percentile increased. In a few grid cells, considering the 50th percentile, the model predicted the earliest ascospore release in February in Egypt and the latest in December in the northwest of Spain. Considering again the 50th percentile, the main period for the onset of ascospore release was from March (i.e. Egypt) and September (i.e. Montenegro) (Fig. 4, Supplementary Material E). Grid cells in Algeria, Cyprus, Egypt, Israel, Jordan, Libya, Syria and Tunisia were relatively homogeneous in terms of the dates of ascospore release, resulting in narrower periods mainly concentrated in late spring and early summer. In contrast, grid cells in Croatia, Greece, France, Italy, Lebanon, Montenegro, Morocco, Portugal, Spain and Turkey presented more heterogeneity in the predicted dates with wider periods of ascospore release, from late spring to early autumn. With the probability threshold of 0.5, no ascospore release was predicted in some areas in Croatia, Italy, Montenegro, Morocco, Portugal, Spain and Turkey. The same trends of the 50th percentile were also observed in the 5th and 95th percentiles, with earlier dates predicted with the 5th percentile and later dates with the 95th percentile. Overall, except in some regions in Morocco and Egypt, the onset of ascospore release was predicted to occur mainly between spring and summer, especially in late spring.

## Discussion

Some studies have considered that the climate conditions in the Mediterranean Basin were not suitable for CBS development. Based on this claim, the adequacy of the phytosanitary measures established by the EU legislation to reduce the risk of entry of *P. citricarpa* has been questioned^26,27^. In our study, the infection component of CBS was simulated with a generic infection model^33^ for *P. citricarpa* ascospores and pycnidiospores. In configuration scenario S1 the model parameters were fixed following previous studies^28,29,31^, whereas in configuration scenario S2 they were estimated by approximate Bayesian computation^35^. The pseudothecium maturation and ascospore release components were predicted with a degree-day model^4^ for *Phyllosticta* spp.

Except for a few regions in Algeria, Croatia, France, Greece, Italy, Montenegro, Morocco, Portugal, Spain, Syria, Tunisia and Turkey, virtually no ascospore infections were simulated in winter, spring and summer for *P. citricarpa* in the Mediterranean Basin with configuration scenario S1 (Fig. 1, Supplementary Materials B, C). With a $T_{min}$ = $15\,\,^\circ \hbox {C}$ and $W_{min}$ = 15 h many wetness periods were considered not suitable for infection by *P. citricarpa* ascospores. However, as in previous studies^29,31^, a substantial percentage of hours in autumn were considered conducive to ascospore infections. Likewise, ascospore infections with S2 were mainly concentrated in spring and autumn (Supplementary Fig. SA1). The role of *P. citricarpa* ascospores in CBS epidemics in Mediterranean conditions or even whether they are present at all is not known. Nevertheless, it is interesting to note that the values for ascospore infections with S1 and S2 in the Cap Bon region in Tunisia, where CBS is present, were similar to those obtained in citrus-growing regions in Europe and Northern Africa (Figs. 1, 3, Supplementary Materials A, C, Table 2).

In the case of *P. citricarpa* pycnidiospores, infections were seldom simulated by the model in winter and summer with S1 (Fig. 2, Supplementary Materials B, D). Nevertheless, in line with previous studies^29,31^, a considerable percentage of hours were simulated that were suitable for infection in spring and particularly in autumn. As in the case of ascospores, the values for pycnidiospore infections obtained with the generic infection model for the CBS-affected areas in Tunisia with S1 and S2 were similar to those in the European citrus-growing regions of the Mediterranean Basin and Northern Africa (Figs. 2, 3, Supplementary Materials A, D, Table 2). Simulation results for both *P. citricarpa* ascospores and pycnidiospores support previous studies indicating the suitability of Mediterranean climates for the development of CBS^24,25,29–31^.

Process-based models, like the generic infection model used in our study, are considered more adequate for non-equilibrium scenarios, where all potentially suitable areas may have not been colonized yet by the disease^36^. However, process-based models still rely on disease distribution data to define thresholds for climate suitability. Magarey et al.^28^ defined a threshold to consider a location suitable for CBS based on the values of the generic infection model for the Eastern Cape in South Africa. However, our study shows that lower number of infection events for *P. citricarpa* ascospores and pycnidiospores were obtained for the locations in Tunisia where CBS is present than those obtained for the Easter Cape (Fig. 3, Supplementary Material A). Therefore, based on our results, the previous threshold for climate suitability^28^ should be decreased. Lower threshold values might be considered in the future whether CBS is able to establish in more arid areas.

In contrast to previous works^29,31^, despite using the same parameterization (S1) as in those studies, the percentage of hours with weather conditions suitable for infection was higher for pycnidiospores than for ascospores (Figs. 1, 2, 3, Supplementary Material A, Table 2). With a $T_{min} = 10\,\,^{\circ }$C and $W_{min}$ = 12 h, parameter values for the generic infection model were less restrictive for pycnidiospores than for ascospores ($T_{min} = 15\,\,^{\circ }$C and $W_{min}$ = 15 h), and thus more wetness periods were initially considered, particularly in spring. To account for the rain-splash dispersal of *P. citricarpa* pycnidiospores, only those wetness periods starting with a precipitation of > 0.2 mm/h^28^ were considered. The value of 0.2 mm corresponds to the resolution of most rain collectors used in agrometeorological stations. However, considering that ERA5-Land provides accumulated precipitation volumes^37^, the process to calculate actual hourly precipitation values^38^ might overestimate resolution, and thus higher precipitation thresholds could be considered also in line with the biological evidence available^7,39^.

Recent implementations of the temperature function of the generic infection model on a global scale with other fungal plant pathogens did not incorporate the effects of humidity on infection risk^40^. In this global study, the generic infection model with the temperature function alone replicated well the overall spatiotemporal patterns obtained when a wetness restriction was considered. However, our results indicated that different values for $W_{min}$ as well as the inclusion of a precipitation threshold influenced not only the overall values but also the seasonal trend of the simulated infection periods, which is of major epidemiological relevance. The ERA5-Land dataset allows the calculation of hourly relative humidity, from which wetness can be estimated using relative humidity thresholds. However, these thresholds may differ depending on the geographical location and the crop^41^. In our study, the relative humidity threshold used was obtained based on visual observations of leaf wetness in irrigated citrus orchards under Mediterranean conditions^42^.

With the use of approximate Bayesian computation (ABC), the generic infection model parameters ^33^ were estimated and their related uncertainty was assessed^35^. Posterior median values were used to fix model parameter values for configuration scenario S2. However, with S2, the simulated values for pycnidiospore infections were anomalously higher than those for ascospores (Supplementary Material A). In fact, these large differences are not in line with the biological evidence available for *P. citricarpa*. The median value estimated for $W_{min}$ was 19.92 h for ascospores, but as low as 6.33 for pycnidiospores. Despite considering the rain-splash dispersal requirement, this considerably low value of $W_{min}$ together with a $T_{min}$ of $8.03\,\,^\circ \hbox {C}$ resulted in most wetness periods being favourable for pycnidiospore infection.

Our study incorporated an inferential procedure to fix the values of the parameters of the generic infection model and, unlike previous studies^28,29,31^, provides an assessment of the uncertainty associated with them (Table 1). The utility and the performance of this inferential method was illustrated by Makowski et al.^35^ for *Plurivorosphaerella nawae*, a fungal pathogen of persimmon. Nevertheless, this methodological approach may be influenced by the definition of the priors and by the number and values of the tested temperatures used to define the wetness duration intervals (i.e. the availability and the quality of the experimental data) ^35^. Therefore, in view of the results obtained, it would be interesting to explore the performance of other prior distributions and ABC algorithms ^43^. Additionally, the scarce data on germination and/or appressorium formation^14,15,18^ evidences the need for controlled experiments for pycnidiospore and ascospore infection using properly identified *P. citricarpa* isolates under different temperature and wetness regimes in order to reduce the uncertainties associated with the outcomes of the generic infection model.

**Table 1:** Numerical descriptive (mean, median, 5% percentile, 95% percentile and standard deviation (SD)) for the prior and posterior distribution of the five model parameters ($T_{min}$, $T_{opt}$ and $T_{max}$ as the minimum, optimum and maximum temperature in $\,\,^\circ \hbox {C}$ and $W_{max}$ and $W_{min}$ as the upper and lower bounds of the leaf wetness duration requirement *W*(*T*), in hours) for *Phyllosticta citricarpa* ascospores (Asc.) and pycndiospores (Pyc.) estimated by approximate Bayesian computation.

Prior distribution						Posterior distribution					
Parameters	Mean	Median	5%	95%	SD	Spore type	Mean	Median	5%	95%	SD
$T_{min}$	7.13	7.09	1.61	12.70	3.56	Asc.	7.50	7.73	1.70	12.79	3.60
						Pyc.	7.70	8.03	1.75	12.90	3.60
$T_{opt}$	18.67	18.70	9.91	27.10	5.45	Asc.	23.16	24.95	11.64	27.79	4.91
						Pyc.	22.91	23.51	16.16	27.20	3.35
$T_{max}$	27.80	28.26	19.65	34.32	4.56	Asc.	30.20	31.62	21.27	34.66	4.19
						Pyc.	31.81	32.41	26.36	34.70	2.51
$W_{min}$	24.98	24.91	4.32	45.74	13.26	Asc.	19.10	19.92	5.23	31.12	7.82
						Pyc.	6.33	6.22	3.43	9.30	1.85
$W_{max}$	60.64	61.86	22.27	92.63	21.79	Asc.	47.06	35.44	24.91	89.42	21.42
						Pyc.	44.80	36.71	14.77	89.33	24.04

The model by Moyo et al.^4^ for ascospore maturation and release was used in our study. With the exception of some areas in Egypt and in the south of Morocco, in general the onset of ascospore release was predicted late in spring (Fig. 4, Supplementary Material E). This result implies that most *P. citricarpa* ascospores would be released during summer and autumn in the Mediterranean Basin, as suggested by previous studies^5^. Virtually no infections were simulated in summer by the generic model (Fig. 1, Supplementary Materials B, C), thus leaving autumn as the critical period for ascospore infection. Similar results were obtained with previous implementations of the generic infection model for *P. citricarpa*^29,31^. Citrus trees are evergreen and three main flushes occur under Mediterranean conditions, concentrated in spring, summer and autumn^44^. Considering that citrus leaves are susceptible to infection for 8–10 months^16^, the simulated ascospore infections in autumn would encounter susceptible leaf tissues. In the case of fruits, it was reported that they are susceptible from fruit set up to 4–7 months later, depending on the country^17^. The simulated ascospore infections in autumn would take place 5–7 months after fruit set. In the case of *P. citricarpa*, experimental evidence of complete ontogenic resistance in citrus fruits is not available. Hence, it is plausible to consider that the autumn ascosporic infections simulated by the model in the Mediterranean Basin would coincide with susceptible fruit on the trees. The disease has a relatively long incubation period and it may take 2–5 months after infection for symptoms to appear^18^. Therefore, late-maturing citrus cultivars that ripen in winter or spring would potentially be the most impacted by those autumn ascosporic infections.

Cold winter temperatures were suggested as one of the main climatic factors limiting the development of CBS in the Mediterranean Basin^26,27^. In fact, in the degree models for *Phyllosticta* ascospores, lower winter temperatures result in delayed release onset and escape from spring infections. However, those degree models are empirical, developed based on observations of ascospore release in particular locations but without experimental control over the explanatory variable (i.e. temperature). Therefore, their extrapolation to other geographic areas with different temperature regimes is problematic and may lead to misleading conclusions. Moyo et al.^4^ improved the model by Fourie et al.^5^ by including additional locations where CBS is present in arid areas in the Easter Cape and Limpopo provinces in South Africa. However, those locations do not completely cover the full range of climatic conditions found in the Mediterranean Basin, where those degree-day models were projected^5^ as in the present study, yielding potentially inaccurate predictions. Data on ascospore dynamics under Mediterranean climate conditions, such as Tunisia, are needed to develop empirical models that are more appropriate for extrapolation to other regions in the Mediterranean Basin.

Ascospores are considered the primary inoculum source of *P. citricarpa* in South Africa, where pycnidiospores are deemed to play a minor epidemiological role^1^. However, pycnidiospores are more relevant in CBS epidemics in areas in the USA^8–10^, Brazil^11^, Australia^6^ and Cuba^12^, which are characterized by large amounts of rainfall. In fact, the number of pycnidiospore infection events simulated by the generic infection model for those locations were among the highest and much greater than the corresponding number of ascosporic infection events (Fig. 3, Table 2). The relative role of pycnidiospores and ascospores in CBS epidemics under Mediterranean conditions is unknown. The generic infection model resulted in similar numbers of ascospore infection events in Tunisia compared with the arid locations in Eastern Cape and Limpopo in South Africa where CBS is present (Fig. 3, Table 2). However, the number of pycnidiospore infection events for those locations in South Africa was substantially higher than in Tunisia. This might indicate that ascospores play a more relevant role than pycnidiospores in CBS epidemics under Mediterranean conditions, but epidemiological studies should be carried out under field conditions to elucidate this.

New species of *Phyllosticta* have recently been described in citrus, including pathogens and endophytes that may coexist in the same area. Molecular methods are required for the identification of these species^45^. Taxonomic uncertainty affects all the modelling studies available on CBS, including species distribution models and process-based models for infection and/or reproduction of *P. citricarpa*. For instance, presence/absence data published before the new molecular methods for *Phyllosticta* spp. identification became available or data from laboratory experiments with isolates identified only morphologically. The same underlying data from EFSA Panel on Plant Health (PLH)^31^ and Magarey et al.^28^ were used in our study to set the configuration scenarios, in the case of pycnidiospores including also a more recent study^15^. Only in this latter work the isolate used in the experiments was identified molecularly, but no evidences of misidentification were found in the other underlying studies considered^13,14^. Hence, these data from different sources were included so taxonomic uncertainty was somehow captured by the models.

Without access to more detailed information, whole administrative regions where the presence of citrus was reported were considered in our study. This resulted in an increased variability in the model outcomes (Supplementary Materials B, E), as non-cultivated areas in those regions were also taken into account. More accurate maps of citrus cultivation in the Mediterranean Basin would be necessary to narrow down the variability in model simulations.

**Table 2:** Locations where citrus black spot (CBS) is either present or absent included in the climate suitability analysis.

	Code	Country	Location	Köppen-Geiger climate classification^68^	Latitude/longitude	Reference
CBS Present	AUS Queensland I	Australia	Emerald	BSh	$23^\circ 32'49.20''\hbox {S}$, $148^\circ 08'52.80''$E	Magarey et al.^28^
	AUS Queensland II	Australia	Gayndah	BSh	$25^\circ 37'01.20''$S, $151^\circ 35'13.20''$E	Magarey et al.^28^
	USA Florida I	United States	Montura	Aw	$26^\circ 41'16.80''$N, $81^\circ 07'12.00''$W	Magarey et al.^28^
	USA Florida II	United States	Polk	Cfa	$27^\circ 39'03.60''$N, $81^\circ 31'12.00''$W	Magarey et al.^28^
	ZAF Eastern Cape I	South Africa	Addo	BSh	$33^\circ 34'08.40''$S, $25^\circ 41'31.20''$E	Magarey et al.^28^
	ZAF Eastern Cape II	South Africa	Kirkwood	BSh	$33^\circ 28'19.20''$S, $25^\circ 32'42.00''$E	Magarey et al.^28^
	ZAF Limpopo I	South Africa	Letsitele	Cwa	$23^\circ 51'36.00''$S, $30^\circ 17'52.80''$E	Magarey et al.^28^
	ZAF Mpmulanga	South Africa	Nelspruit	Cwa	$25^\circ 27'18.00''$S, $30^\circ 58'19.20''$E	Magarey et al.^28^
	ZAF Limpopo II	South Africa	Ohrigstad	Cwb	$24^\circ 39'08.00''$S, $30^\circ 37'54.40''$E	Moyo et al.^4^
	ZAF Limpopo III	South Africa	Musina	BSh	$22^\circ 38'12.10''$S, $30^\circ 08'07.30''$E	Moyo et al.^4^
	ZAF Eastern Cape III	South Africa	Sundland	BSh	$33^\circ 30'40.70''$S, $25^\circ 39'20.80''$E	Moyo et al.^4^
	BRA São Paulo I	Brazil	Tambaú	Aw	$21^\circ 30'52.00''$S, $47^\circ 12'01.00''$W	Silva Junior et al.^56^
	BRA São Paulo II	Brazil	Río Claro	Aw	$22^\circ 25'00.00''$S, $47^\circ 18'00.00''$W	Bellote et al.^57^
	BRA São Paulo III	Brazil	Itapetininga	Cfa	$23^\circ 36'00.00''$S, $48^\circ 18'00.00''$W	de Andrade et al.^58^
	BRA Rio Grande do Sul	Brazil	Santa C. do Rio Pardo	Cfa	$22^\circ 50'00.00''$S, $49^\circ 21'00.00''$W	de Andrade et al.^58^
	ARG Córdoba	Argentina	Córdoba	Cfa	$28^\circ 34'28.00''$S, $58^\circ 42'32.00''$W	Rodríguez et al.^59^
	ARG Buenos Aires	Argentina	Buenos Aires	Cfa	$27^\circ 45'00.00''$S, $57^\circ 37'00.00''$W	Rodríguez et al.^59^
	Maharashtra I	India	Nawegaon	Aw	$21^\circ 13'59.99''$N, $79^\circ 22'0.0001''$E	Daset et al^60^
	IND Maharashtra I	India	Pathar	Aw	$21^\circ 30'00.00''$N, $79^\circ 1'59.8700''$E	Daset et al.^60^
	TUN Nabeul	Tunisia	Nabeul	Csa	$36^\circ 27'04.70''$N, $10^\circ 44'08.40''$E	Boughalleb-M’Hamdi et al.^21^
	TUN Soliman	Tunisia	Soliman	Csa	$36^\circ 41'47.90''$N, $10^\circ 29'35.00''$E	Boughalleb-M’Hamdi et al.^21^
	TUN Menzel Bou Zelfa	Tunisia	Menzel BouZelfa	Csa	$36^\circ 40'59.80''$N, $10^\circ 34'59.00''$E	Boughalleb-M’Hamdi et al.^21^
	TUN Grombalia	Tunisia	Grombalia	Csa	$36^\circ 36'09.20''$N,$10^\circ 30'05.40''$E	Boughalleb-M’Hamdi et al.^21^
	TUN Béni Khalled	Tunisia	Béni Khalled	Csa	$36^\circ 38'52.50''$N,$10^\circ 35'29.30''$E	Boughalleb-M’Hamdi et al.^21^
	TUN Bou Argoub	Tunisia	Bou Argoub	Csa	$36^\circ 31'51.70''$N, $10^\circ 33'08.00''$E	Boughalleb-M’Hamdi et al.^21^
	TUN Dar Chaabane Al Fehri	Tunisia	Dar Chaabane Al Fehri	Csa	$36^\circ 27'45.70''$N,$10^\circ 44'57.30''$E	Boughalleb-M’Hamdi et al.^21^
CBS Absent	ESP Valencian Community	Spain	Valencia	BSk	$39^\circ 36'00.00''$N, $0^\circ 24'00.00''$W	For CBS absent locations, coordinates are from the grid cells centroids
	POR Faro	Portugal	Faro	Csa	$37^\circ 0.0'45.25''$N, $7^\circ 54'04.10''$W	
	MAR Taroudannt	Morocco	Taroudannt	BWh	$30^\circ 30'00.00''$N , $8^\circ 48'00.00''$W	
	DZA Skikda	Algeria	Skikda	Csa	$36^\circ 48'00.00''$N , $6^\circ 48'00.00''$E	
	FRA Corsica	France	Corsica	Csa	$42^\circ 17'56.26''$N, $9^\circ 29'55.50''$E	
	MAL Attard	Malta	Attard	Csa	$35^\circ 53'23.00''$N, $14^\circ 25'16.50''$E	
	ITA Calabria	Italy	Calabria	Csa	$38^\circ 13'06.24''$N, $16^\circ 14'11.54''$E	
	HRV Dubrovnik-Neretva	Croatia	Dubrovnik-Neretva	Csa	$43^\circ 00'1.04''$N, $17^\circ 35'59.14''$E	
	MNT Bar	Montenegro	Bar	Csa	$42^\circ 06'27.47''$N, $19^\circ 06'44.39''$E	
	GRC Peloponnese	Greece	Peloponnese	Csa	$37^\circ 36'11.92''$N, $22^\circ 41'47.94''$E	
	LBY Darnah	Libya	Darnah	BSh	$32^\circ 41'49.88''$N, $22^\circ 41'53.20''$E	
	EGY Al-Buhaira	Egypt	Al-Buhaira	BWh	$31^\circ 00'00.00''$N, $30^\circ 36'00.00''$E	
	TUR Mediterranean	Turkey	Mediterranean	Csa	$37^\circ 00'00.00''$N, $35^\circ 06'00.00''$E	
	CYP Limassol	Cyprus	Limassol	Csa	$34^\circ 42'20.48''$N, $33^\circ 00'05.00''$E	
	JOR Irbib	Jordan	Irbib	Csa	$32^\circ 30'00.00''$N, $35^\circ 48'00.00''$E	
	LBN Akkar	Lebanon	Akkar	Csa	$34^\circ 35'47.29''$N, $36^\circ 00'55.33''$E	
	SYR Latakia	Syria	Latakia	Csa	$35^\circ 30'07.42''$N, $35^\circ 54'06.80''$E	
	ISR Central	Israel	Central	Csa	$32^\circ 00'00.00''$N, $34^\circ 48'00.00''$E	

## Methods

### Study area

The study area was set considering the main citrus-growing countries in the Mediterranean Basin (Algeria, Croatia, Cyprus, Egypt, France, Greece, Israel, Italy, Jordan, Lebanon, Libya, Malta, Montenegro, Morocco, Portugal, Spain, Syria, Tunisia and Turkey). For Cyprus, France, Greece, Italy, Portugal, Spain and Turkey, citrus-growing regions were identified at NUTS3 level based on EUROSTAT data^46^. For the other countries: Algeria^47^, Egypt^48^, Israel^49^, Jordan^50^, Lebanon^51^, Libya^52^, Morocco^53^, Syria^54^ and Tunisia^55^, as well as for 19 georeferenced locations where CBS is present outside the study area: Australia^28^, USA^28^, Brazil^56–58^, Argentina^59^, India^60^ and South Africa^4,28^ (Table 2), identification was performed through a literature search. CBS occurrence locations within and outside the study area were used to compare them with the citrus-growing regions in the Mediterranean Basin where the disease is officially absent^19^.

### Climatic data

Climatic variables for the study area and the selected overseas locations from 2009 to 2018 were obtained from the ERA5-Land dataset^61^ via the Clinate Data User (CDS) user interface. ERA5-Land produces a total of 50 variables from 1950 to the present describing the water and energy cycles over land, globally, hourly and at a gridded spatial resolution of 0.1 $\times$ 0.1$^{\circ }$ ($\sim$ 9 km)^37^. Hourly data were downloaded from ERA5-Land for air temperature at 2 m (K), dew point temperature at 2 m (K) and total precipitation (m). In ERA5-Land, total precipitation (m) is the accumulated volume from 00 UTC of day=D at each hour until 00 UTC of day = D + 1^38^. The actual precipitation during each hour was then obtained by calculating the increase in precipitation volume in mm from the previous hour. Dew point temperature was used to calculate relative humidity (RH) as a % in accordance with Wallace and Hobbs^62^ as:

1 $$\begin{aligned} RH \,=\, 100\cdot \left( \frac{e_{s}(Td)}{e_{s}(T)}\right) \end{aligned}$$

with $e_{s}(Td)$ and $e_{s}(T)$ denoting actual vapour pressure and saturation vapour pressure in hPa, respectively; with *Td* as the dew point temperature at 2 m and *T* as the air temperature at 2 m both in $^\circ \hbox {C}$ and for $e_{s}(Td)\le e_{s}(T)$. Actual and saturation vapour pressure were estimated following Bolton^63^ as:

2 $$\begin{aligned} \begin{aligned} e_{s}(Td)\,&=\, 6.12\cdot \exp {\left[ (17.67\cdot Td/(243.5 + Td)\right] } \\ e_{s}(T)\,&=\, 6.12\cdot \exp {\left[ (17.67\cdot T/(243.5 + T)\right] } \end{aligned} \end{aligned}$$

Based on relative humidity, the hourly occurrence of leaf wetness (i.e. visible presence of water on a plant surface)^64^ was considered for those relative humidity values equal to or higher than 87.5%^42^.

Daily maximum, minimum and mean temperature; total precipitation^38^; and mean relative humidity were computed from the hourly variables. Daily vapour pressure deficit (*VPD*) was estimated from mean values of temperature ($T_{mean}$) and relative humidity ($RH_{mean}$) in accordance with Moyo et al.^4^:

3 $$\begin{aligned} VPD\,=\, \left( \frac{1 - RH_{mean}}{100}\right) \cdot 6.11\cdot \exp {\left[ (17.47\cdot T_{mean})/(239 + T_{mean})\right] } \end{aligned}$$

with $T_{mean}$ in $^\circ \hbox {C}$, $RH_{mean}$ in % and *VPD* in hPa.

### Generic infection model

The occurrence of infection events and percentage of hours with weather conditions suitable for infection by *P. citricarpa* ascospores or pycnidiospores were computed using the generic infection model developed by Magarey et al.^33^.

This model simulates the leaf wetness duration requirement (*W*(*T*), in hours) to achieve a critical disease intensity at a given temperature *T*. The model uses a temperature response function^65,66^, (*f*(*T*)), to estimate the probability of infection considering the pathogen’s cardinal temperatures:

4 $$\begin{aligned} f(T) = \left\{ \begin{matrix} \left( \frac{T_{max} - T}{T_{max} - T_{opt}}\right) \left( \frac{T - T_{min}}{T_{opt} - T_{min}}\right) ^{(T_{opt} - T_{min})/( T_{max} - T_{opt})} &{} \text{ if }\,\,\, T_{min} \le T \le T_{max} \\ \\ 0 &{} ,\text{ otherwise }\end{matrix}\right. \end{aligned}$$

with $T_{min}$, $T_{opt}$ and $T_{max}$ as the minimum, optimum and maximum temperature in °C for infection, respectively, and *T* as the hourly mean temperature during the wetness period. Thus, the wetness duration requirement (*W*(*T*)) for the critical disease threshold at temperature *T* is computed as:

5 $$\begin{aligned} W(T) = \left\{ \begin{matrix} W_{min}/f(T) &{} \text{ if }\,\,\, W_{min}/f(T) \le W_{max} \\ \\ W_{max} &{} ,\text{ otherwise }\end{matrix}\right. \end{aligned}$$

with $W_{max}$ and $W_{min}$ as the upper and lower bounds of *W*(*T*) in hours.

The critical dry-period interruption parameter ($D_{50}$) is defined as the duration of a dry period at relative humidities $<95\%$ that will result in a $50\%$ reduction in disease compared with a continuous wetness period and determines the additivity of two interrupted wet periods^33^.

The length of the wetness periods ($W(T)_{data}$) and their corresponding mean temperature (i.e. hourly mean temperature) (*T*) were computed for the 10-year period under consideration (2009–2018) in the 9-km grid cells set by the climatic ERA5-Land dataset for the study area comprising the citrus-growing regions in the Mediterranean Basin, as well as for the 19 locations selected outside the study area. In the case of pycnidiospores, to account for the rain-splash dispersal requirement, only those infection periods starting with a precipitation > 0.2 mm/h^28^ were considered.

The generic infection model was run under the two configuration scenarios indicated below. For both pycnidiospores and ascospores, an infection event was considered to occur if *W*(*T*) simulated by the model under the computed mean temperature (*T*) was less than or equal to $W(T)_{data}$. The length in hours of each infection event coincides then with $W(T)_{data}$. The resulting number of infection events and their corresponding length in hours were simulated for each month and year over the 10-year period (2009–2018). The percentage of hours with weather conditions suitable for successful infection was calculated based on the total number of hours for each month and year.

#### Model parameterization

The model parameter values were fixed according to two configuration scenarios. For the first configuration scenario (S1), parameter values were set following previous studies on *P. citricarpa*^28,29,31^: for ascospores, $T_{min}$ = $15\,\,^\circ \hbox {C}$, $T_{opt}$ = $27\,\,^\circ \hbox {C}$, $T_{max}$ = $35\,\,^\circ \hbox {C}$, $W_{min}$ = 15 h and $W_{max}$ = 38 h; and for pycnidiospores they were set as $T_{min} = 10\,\,^{\circ }\hbox {C}$, $T_{opt}$ = $25\,\,^\circ \hbox {C}$, $T_{max}$ = $35\,\,^\circ \hbox {C}$, $W_{min}$ = 12 h and $W_{max}$ = 35 h. $D_{50}$ was fixed at 3 h, for both ascospores and pycnidiospores.

For the second configuration scenario (S2), $D_{50}$ was also fixed at 3 h for both ascospores and pycnidiospores, while the rest of the parameters were estimated from two sources of information: i) prior information about the parameter values derived from the literature, and ii) the possible range of values for *W*(*T*) for one or several temperatures *T* derived from experimental data. Both sources of information were combined using inferential Bayesian procedures.

For either ascospores or pycnidiospores, prior information about the five model parameters were defined in probabilistic terms by means of uniform distributions (*U*(*a*, *b*)) in which lower (*a*) and upper bounds (*b*) were defined as per Makowski et al.^35^ based on values reported by Magarey et al.^33^ for a series of fungal pathogens: $T_{min}\sim U(1,13.3)$, $T_{opt}\sim U(8.5,28)$, $T_{max}\sim U(18,35)$, $W_{min}\sim U(2,48)$ and $W_{max}\sim U(6,96)$.

A possible range of values for *W*(*T*) were extracted from laboratory experiments with isolates identified as *P. citricarpa*. For ascospores, data on germination were extracted from Kotzé^13^. Appressorium formation was not reported in this experiment. For pycnidiospores, data on appressorium formation were extracted from Noronha^14^ and Wang and Dewdney^15^. Only in this latter study the isolate used was identified molecularly.

Lower ($W_{LOW}(T)$) and/or upper ($W_{UP}(T)$) bounds were defined for *W*(*T*). The lower bound ($W_{LOW}(T)$) was defined as the highest wetness duration leading to 0% germination for ascospores or appressorium formation for pycnidiospores. The upper bound ($W_{UP}(T)$) was defined as the lowest duration leading to a minimum 60% germination for ascospores or 70% appressorium formation for pycnidiospores.

In the study by Kotzé^18^ values equal to or greater than 60% germination were obtained at $29.5\,\,^\circ \hbox {C}/36\,\hbox {h}$, while 0 and 9.2% germination were recorded at $15\,\,^\circ \hbox {C}/23\,h$ and $15\,\,^\circ \hbox {C}/36\,h$, respectively, and thus we set $W(15\,\,^{\circ }\text{C})>23\,\text{ h }$, $W(29.5\,\,^{\circ }\text{C})< 36\,\text{ h }$. In the study by Noronha^14^ values equal to or greater than 70% of appressorium formation were obtained at $15\,\,^\circ \hbox {C}/48\,\hbox {h}$ and $30\,\,^\circ \hbox {C}/36\,\hbox {h}$, while 0 and  7% appresorium formation were recorded at $10\,\,^\circ \hbox {C}/12\,\hbox {h}$ and $10\,\,^\circ \hbox {C}/24\,\hbox {h}$, respectively. Thus, we set $W(15\,\,^{\circ }\text{C})< 48\,\text{ h }$, $W(30\,\,^{\circ }\text{C})< 36\,\text{ h }$ and $W(10\,\,^{\circ }\text{C})> 12\,\text{ h }$. In the study by Wang and Dewdney^15^, values equal to or greater than 70% of appressorium formation were obtained at $24\,\,^\circ \hbox {C}/10\,\hbox {h}$ while 0 and  19% appressorium formation were recorded at $24\,\,^\circ \hbox {C}/4\,\hbox {h}$ and $24\,\,^\circ \hbox {C}/6\,\hbox {h}$, respectively, hence we fixed $4\,\text{ h }<W(24\,\,^{\circ }\text{C})< 10\,\text{ h }$.

Given the nature of the data, i.e. the wetness duration requirement for a given *T* was defined over a range of values, approximate Bayesian computation (ABC)^35^ was used to compute parameter posterior distributions as defining the likelihood function becomes difficult for this type of data. ABC was addressed using the rejection algorithm developed by Marjoram et al.^67^ as implemented by Makowski et al.^35^ to estimate the following posterior distributions for ascospores and pycnidiospores, respectively:

$$\begin{aligned}&\pi \left[ \Theta \mid W(10\,\,^{\circ }\text{C})> 12\,\text{ h }, \,\,W(15\,\,^{\circ }\text{C})< 48\,\text{ h },\,\, 4\,\text{ h }<W(24\,\,^{\circ }\text{C})< 10\,\text{ h }, \,\,W(30\,\,^{\circ }\text{C})< 36\,\text{ h } \right] \\&\pi \left[ \Theta \mid W(15\,\,^{\circ }\text{C})>23\,\text{ h },\,\, W(29.5\,\,^{\circ }\text{C})< 36\,\text{ h } \right] \end{aligned}$$

with $\Theta \,=\,(T_{min}, T_{opt}, T_{max}, W_{min}, W_{max})$ denoting the five model parameters. Due to overlap of the prior distributions, the following constraints: $T_{max}>T_{opt}>T_{min}$ and $W_{max}>W_{min}$ were established to address the simulation. A total of 50,000 simulations were carried out for each type of spore, of which 5358 were accepted for ascospores and 1355 simulations for pycnidiospores. The computations were replicated five times in order to assess the stability of the results. Prior and posterior distributions of parameter values obtained for *P. citricarpa* ascospores and pycnidiospores were summarized by their mean, median, the 5% and 95% percentiles, and SD (Table 1). In general, the mean and median for each parameter showed relatively similar values in the prior and posterior distributions, respectively.

For ascospores, $T_{min}$, $T_{opt}$ and $T_{max}$ showed similar values in the prior and posterior distributions, respectively. Compared with the priors, the posterior means increased from 5 to 24% and from 9 to 33% in the case of the medians. The posterior standard deviations and the 5–95% interval of the three cardinal temperature parameters slightly decreased in relation to the priors, except for $T_{min}$, which remained stable. The posterior mean and median of $W_{min}$ presented similar values, but those for $W_{max}$ showed notable differences. Posterior means and medians decreased in relation to the priors, by up to 43% in the case of the median of $W_{max}$. Compared with the priors, the posterior standard deviation and the 5–95% interval were substantially reduced for $W_{min}$ but not for $W_{max}$.

For pycnidiospores, the posterior distribution of $T_{min}$, $T_{opt}$ and $T_{max}$ increased compared with their corresponding priors, from 8 to 23% for the means and from 13 to 26% for the medians. The posterior standard deviation and the 5–95% interval of the cardinal temperature parameters decreased in relation to the priors, except for $T_{min}$, which remained stable. The posterior mean and median of $W_{min}$ presented similar values, but those for $W_{max}$ showed notable differences. Posterior means and medians decreased in relation to the priors, by up to 75% in the case of the mean of $W_{min}$. The standard deviation and the 5–95% interval for the posterior $W_{min}$ were much smaller than its prior, but slightly greater in the case of $W_{max}$.

The posterior means and medians of $T_{min}$, $T_{opt}$, $T_{max}$ and $W_{max}$ were similar for ascospores and pycnidiospores. However, in the case of $W_{min}$ they were much lower for pycnidiospores (6.33 vs. 19.10 for the mean and 6.22 vs. 19.92 for the median).

For the second configuration scenario (S2), the generic infection model was run considering the median values of the approximated posterior distributions (Table 1). Specifically, parameter values for ascospores were $T_{min}$ = $7.73\,\,^\circ \hbox {C}$, $T_{opt}$ = $24.95\,\,^\circ \hbox {C}$, $T_{max}$ = $31.62\,\,^\circ \hbox {C}$, $W_{min}$ = 19.92 h and $W_{max}$ = 35.44 h; and for pycnidiospores $T_{min} = 8.03\,\,^{\circ }$C, $T_{opt}$ = $23.51\,\,^\circ \hbox {C}$, $T_{max}$ = $32.41\,\,^\circ \hbox {C}$, $W_{min}$ = 6.22 h and $W_{max}$ = 36.71 h.

### Pseudothecium maturation and onset of ascospore release model

A degree-day model developed by Moyo et al.^4^ was used to predict pseudothecium maturation and subsequent ascospore release of *P. citricarpa*. This model was built on previous work by Fourie et al.^5^ and computes the probability of first ascospore release (*PFAR*) as follows:

6 $$\begin{aligned} PFAR \,=\, \exp (-\exp (-(-3.131 + 0.007 \cdot DDtemp - 0.007 \cdot DDwet))) \end{aligned}$$

with *DDtemp* denoting cumulative degree-days and calculated as $DDtemp = ((Tmin + Tmax) / 2) - \text{ base } \text{ temp }$, with a base temperature of 10 $^{\circ }$C^5^, and *DDwet* as cumulative degree-days for rainy or humid days ($DDwet\,=\,DDtemp$ accumulation only on days with measurable rainfall ($>\,0.1\,\hbox {mm}$) or *VPD* $<\,5$ hPa). Cumulative degree-days were computed from the daily climatic data within the study area comprising the citrus-growing regions in the Mediterranean Basin at the 9-km grid spatial resolution fixing the biofix on the 1st January.

The date of the first meaningful discharge of *P. citricarpa* ascospores (> 5 ascospores trapped per day) was estimated by fixing the threshold of *PFAR* in 0.5^4^. This date, defined as the onset of ascospore release, was simulated over the 10-year period from 2009 to 2018 for each grid cell within the study area. The between-year variability in the onset of ascospore release across the ten years under consideration were summarized for each 9-km cell by the 5th, 50th and 95th percentiles.

## Supplementary Information

Supplementary Information 1.

Supplementary Information 2.

Supplementary Information 3.

Supplementary Information 4.

Supplementary Information 5.

## Data Availability

Following open-science principles, once our work is accepted for publication, data and code will be deposited in a public repository to enhance reproducibility and replicability. To make data and code available in the revision process, they have been uploaded as supplementary material. All data used in this study can be downloaded from the ERA5-Land dataset^61^ via the Climate Data Store (CDS) https://cds.climate.copernicus.eu/cdsapp#/dataset/reanalysis-era5-land?tab=formweb interface. Data curation and formal analysis have been implemented in the *R* statistical programming ^69^. All the generic code used in the research is open-source and available at: https://zenodo.org/record/7313040#.Y24Q73bMKUk.
