# Supplementary material for: Climate suitability of the Mediterranean Basin for citrus black spot disease (Phyllosticta citricarpa) based on a generic infection model
Source: Sci Rep. 2022 Nov 18;12:19876. doi: 10.1038/s41598-022-22775-z (PMC9674692; doi:10.1038/s41598-022-22775-z)
Supplement: Supplementary file 1 — Supplementary Information 1. [file 41598_2022_22775_MOESM1_ESM.pdf]

Supplementary Material A. Climate suitability of the Mediterranean Basin for citrus black spot disease (*Phyllosticta citricarpa*) based on a generic infection model. Figures of configuration scenario S2 for ascospores and pycnidiospores

Anaïs Galvañ[1], Naima Boughalleb-M'Hamdi[2], Najwa Benfradj[2], Sabrine Mannai[2], Elena Lázaro[1,+], and Antonio Vicent[1,+,*]

[1]Institut Valencià d'Investigacions Agràries (IVIA), Centre de Protecció Vegetal i Biotecnologia, 46113 Moncada, Valencia, Spain
[2]Department of Biological Sciences and Plant Protection,Institut Supérieur Agronomique de Chott Mariem, LR21AGR05, University of Sousse,Chott Mariem,Sousse, 4042, Tunisia
[*]vicent_antciv@gva.es
[+]These authors contributed equally to this work

Supplementary Figures SA1 to SA3

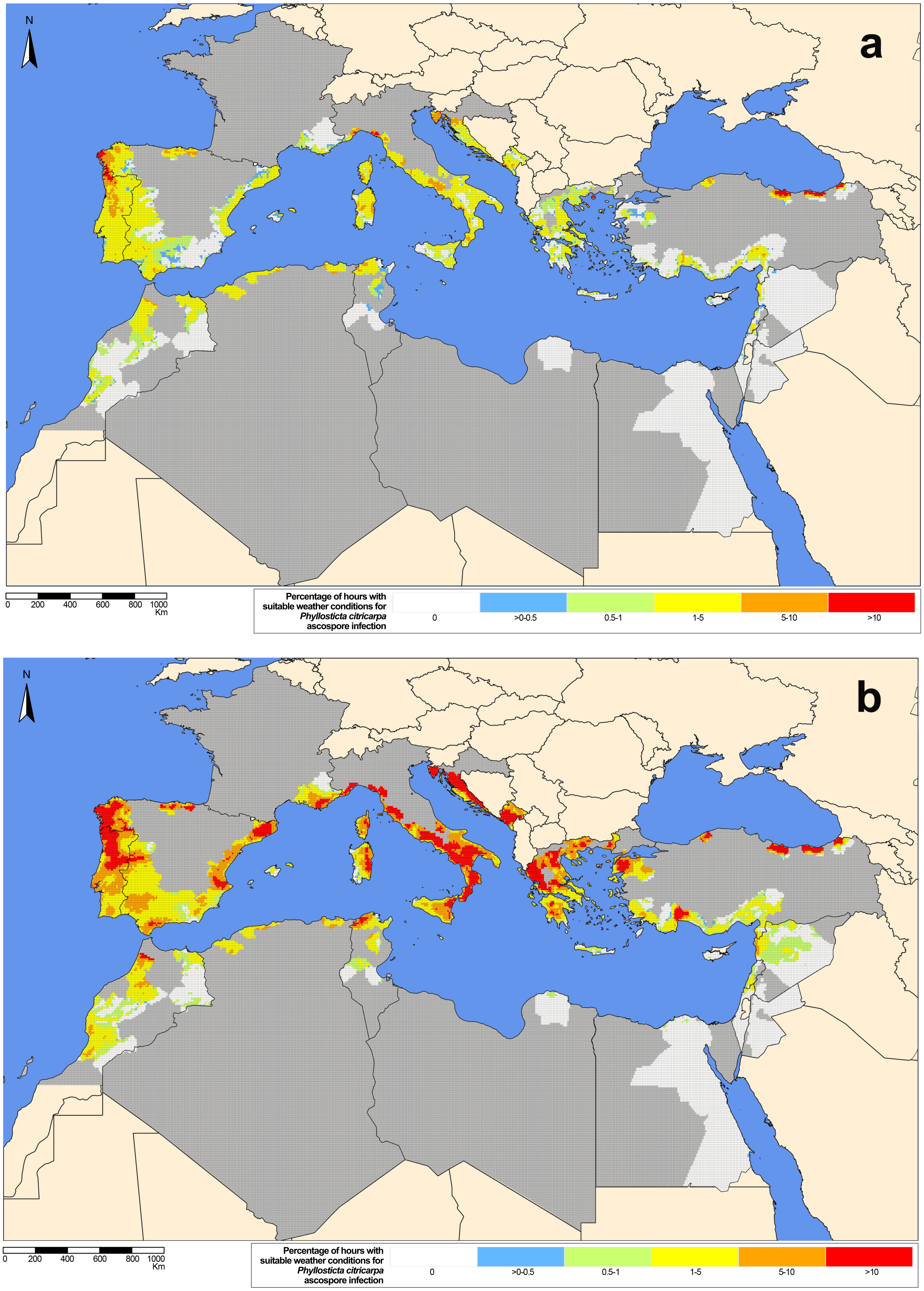

Figure SA1. Percentage of hours ($= 0$ white, $]0 - 0.5]$ blue, $]0.5 - 1]$ green, $]1 - 5]$ yellow, $]5 - 10]$ orange, > 10 red) with suitable weather conditions for *Phyllosticta citricarpa* ascospore infection (generic infection model for foliar fungal pathogens by Magarey et al.[1], configuration scenario S2) for the 9-km grid interpolated climatic data of the citrus-growing regions in the Mediterranean Basin from 2009 to 2018 for **(a)** spring (March to May) and **(b)** autumn (September to November). Non citrus areas inside citrus-growing countries in dark-grey. The maps were created by the authors using the software `R` 3.6.0, `https://www.R-project.org`.

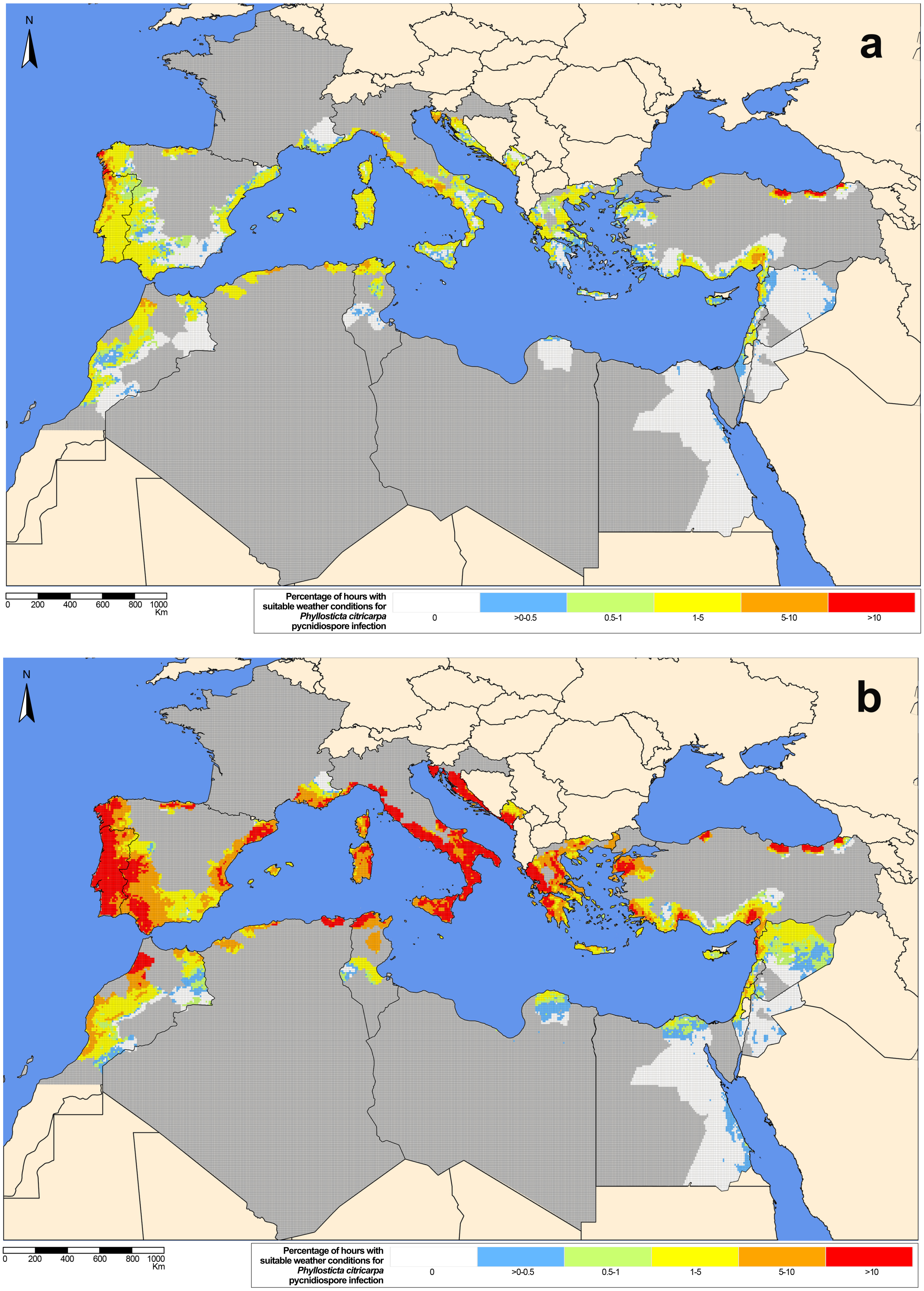

Figure SA2. Percentage of hours ($= 0$ white, $]0-0.5]$ blue, $]0.5-1]$ green, $]1-5]$ yellow, $]5-10]$ orange, > 10 red) with suitable weather conditions for *Phyllosticta citricarpa* pycnidiospore infection (generic infection model for foliar fungal pathogens by Magarey et al.[1], configuration scenario S2) for the 9-km grid interpolated climatic data of the citrus-growing regions in the Mediterranean Basin from 2009 to 2018 for **(a)** spring (March to May) and **(b)** autumn (September to November). Non citrus areas inside citrus-growing countries in dark-grey. The maps were created by the authors using the software `R` 3.6.0, `https://www.R-project.org`.

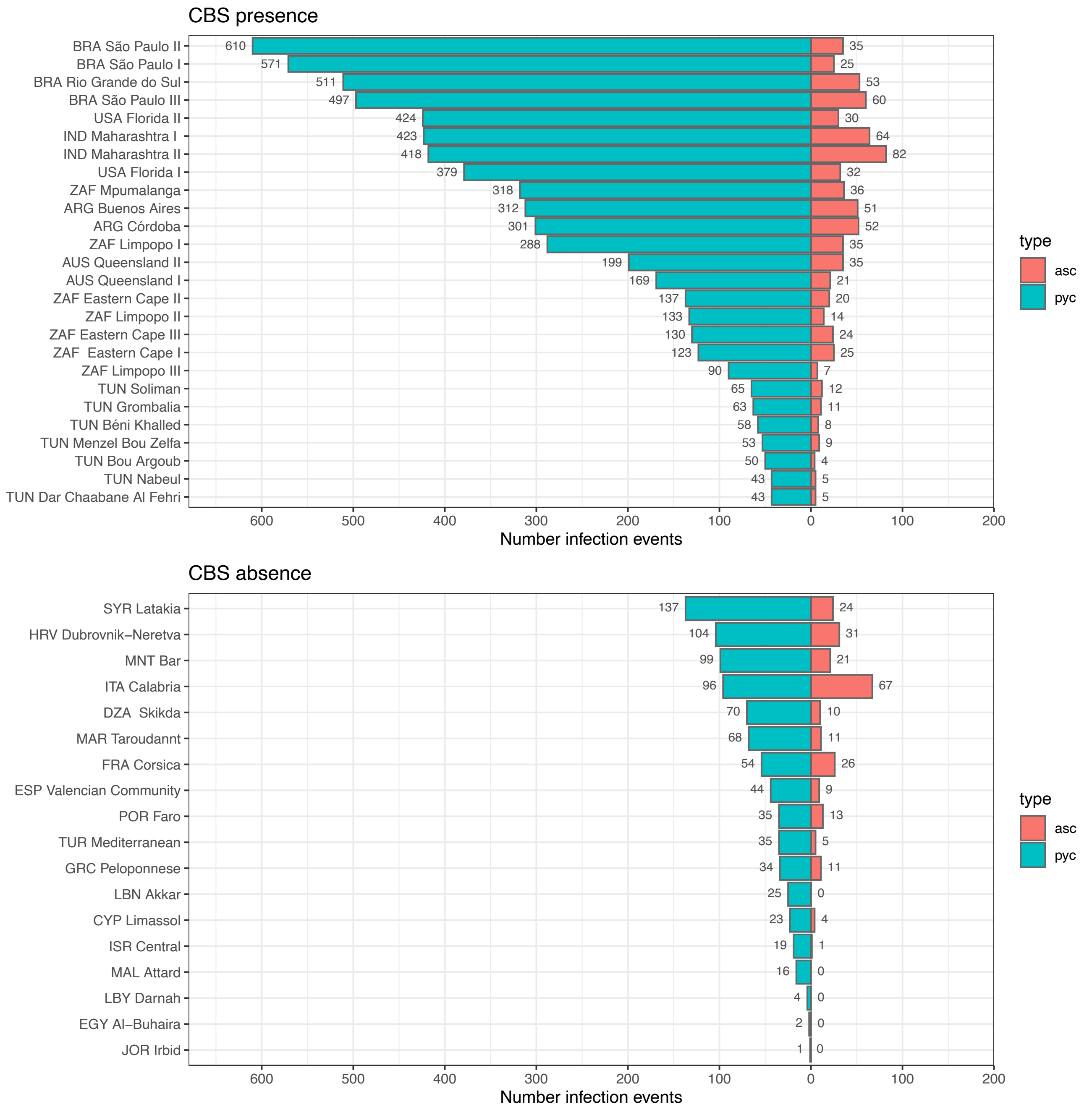

Figure SA3. Number of infection events for *Phyllosticta citricarpa* ascospores (asc) and pycnidiospores (pyc) from 2009 to 2018 simulated by the generic infection model for foliar fungal pathogens by Magarey et al.[1] (configuration scenario S2) for locations where citrus black spot (CBS) is either present or absent. Number of infection events in x-axis and selected locations where CBS is either present or absent in y-axis. Ascospores (asc) in red and pycnidiospores (pyc) in turquoise.

References

1. Magarey, R., Sutton, T. & Thayer, C. A simple generic infection model for foliar fungal plant pathogens. *Phytopathology* **95**, 92–100 (2005).
