# Supplementary material for: Climate suitability of the Mediterranean Basin for citrus black spot disease (Phyllosticta citricarpa) based on a generic infection model
Source: Sci Rep. 2022 Nov 18;12:19876. doi: 10.1038/s41598-022-22775-z (PMC9674692; doi:10.1038/s41598-022-22775-z)
Supplement: Supplementary file 2 — Supplementary Information 2. [file 41598_2022_22775_MOESM2_ESM.pdf]

Supplementary Material B. Climate suitability of the Mediterranean Basin for citrus black spot disease (*Phyllosticta citricarpa*) based on a generic infection model. Figures of configuration scenario S1 for ascospore and pycnidiospore infection dynamics

Anaïs Galvañ[1], **Naima Boughalleb-M'Hamdi**[2], **Najwa Benfradj**[2], **Sabrine Mannai**[2], **Elena Lázaro**[1,+], **and Antonio Vicent**[1,+,*]

[1]Institut Valencià d'Investigacions Agràries (IVIA), Centre de Protecció Vegetal i Biotecnologia, 46113 Moncada, Valencia, Spain
[2]Department of Biological Sciences and Plant Protection,Institut Supérieur Agronomique de Chott Mariem, LR21AGR05, University of Sousse,Chott Mariem,Sousse, 4042, Tunisia
[*]vicent_antciv@gva.es
[+]These authors contributed equally to this work

Supplementary Figures SB1 to SB12

Ascospore infection dynamics

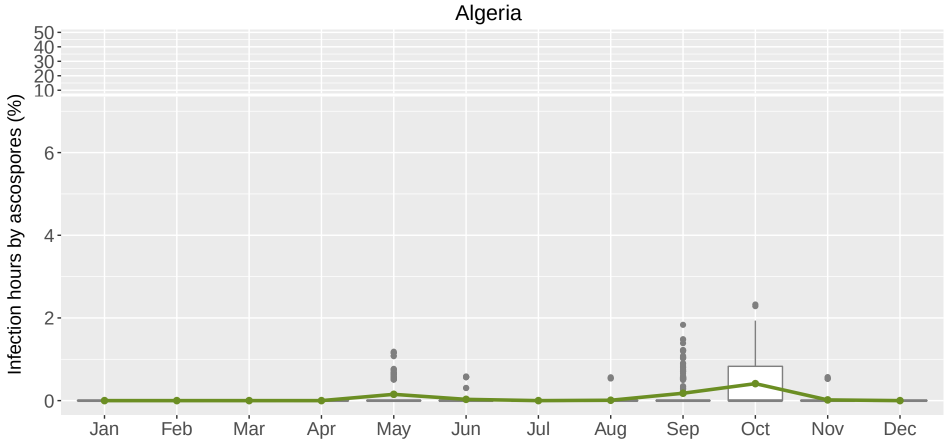

(a)

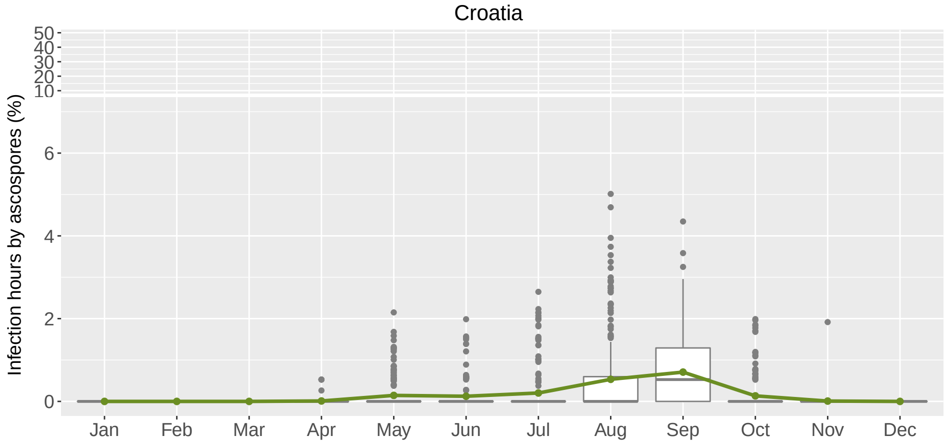

(b)

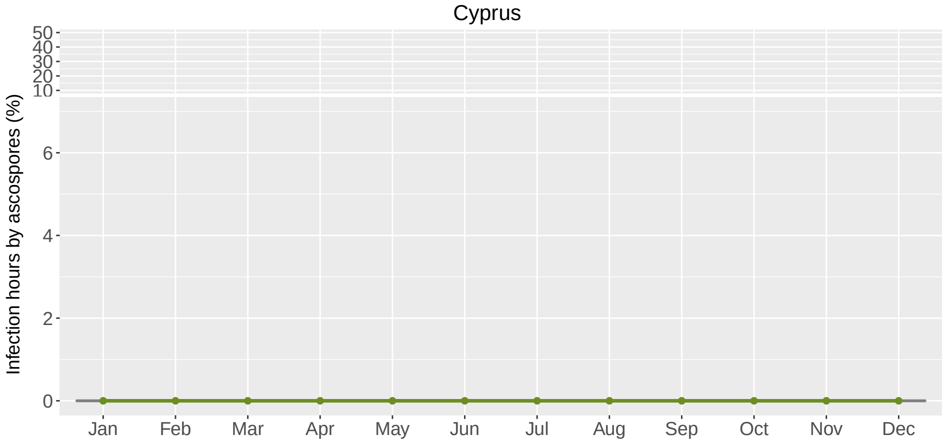

(c)

Figure SB1. Monthly percentage of hours with weather conditions suitable for successful infection events by *Phyllosticta citricarpa* ascospores (generic infection model for foliar fungal pathogens by Magarey et al.[1], configuration scenario S1) for 9-km grid interpolated climatic data from 2009 to 2018. Box-and-whisker plots for the citrus-growing regions in (a) Algeria, (b) Croatia, and (c) Cyprus. Outliers represented by grey dots, and green line denotes the mean trend.

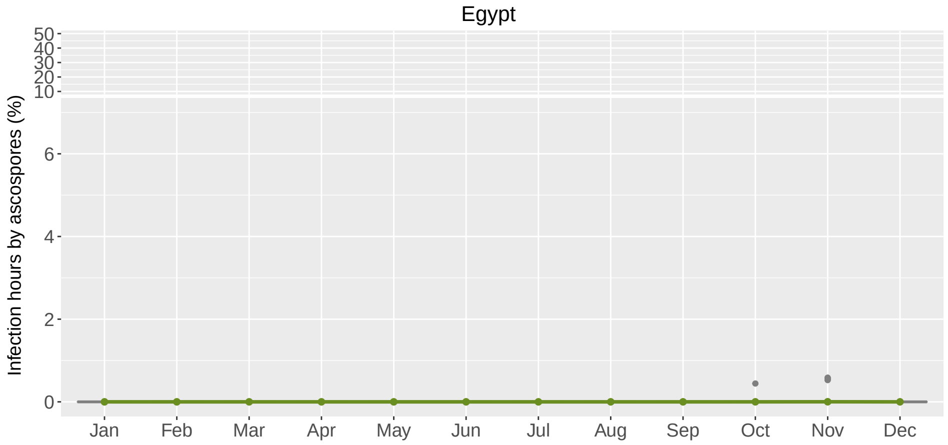

(a)

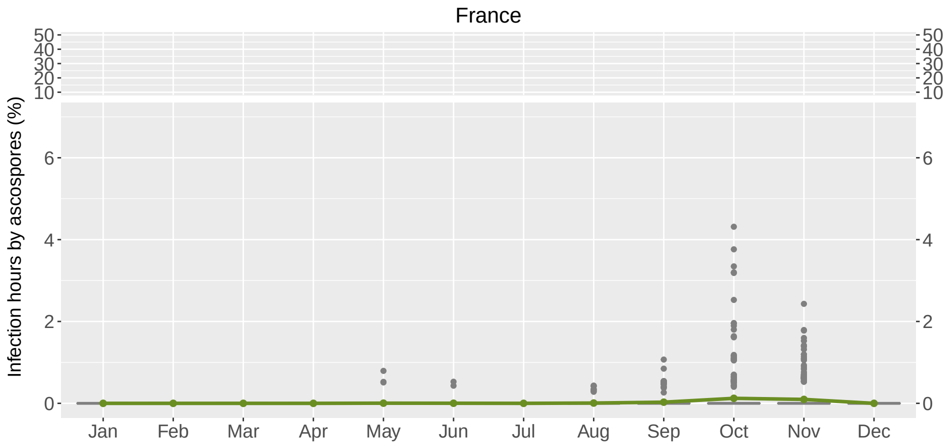

(b)

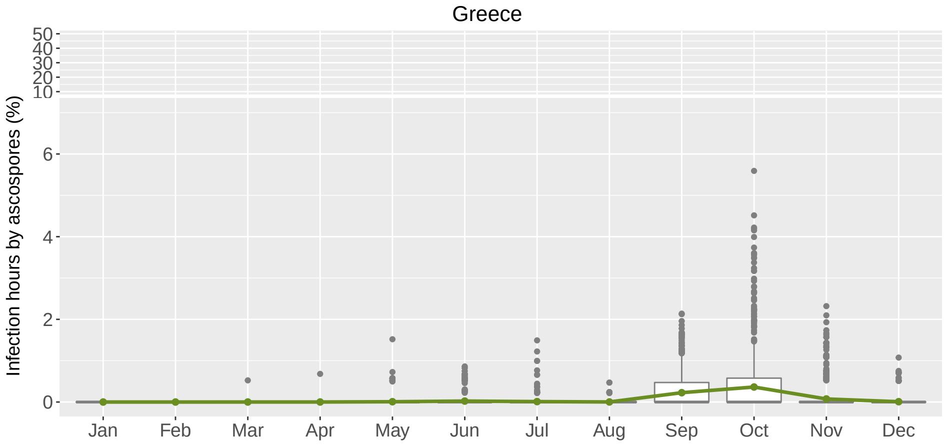

(c)

Figure SB2. Monthly percentage of hours with weather conditions suitable for successful infection events by *Phyllosticta citricarpa* ascospores (generic infection model for foliar fungal pathogens by Magarey et al.[1], configuration scenario S1) for 9-km grid interpolated climatic data from 2009 to 2018. Box-and-whisker plots for the citrus-growing regions in (a) Egypt, (b) France, and (c) Greece. Outliers represented by grey dots, and green line denotes the mean trend.

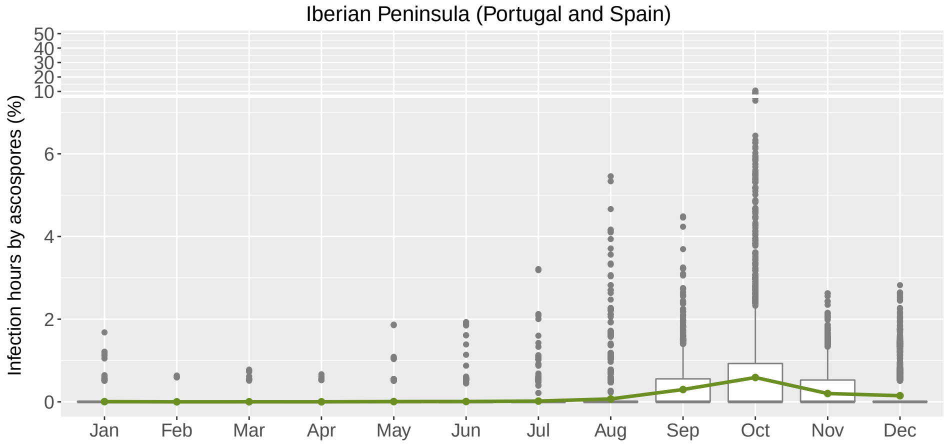

(a)

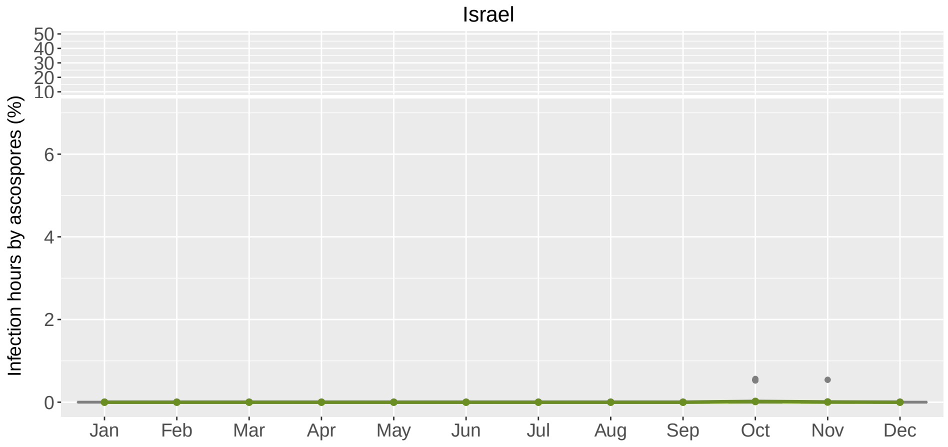

(b)

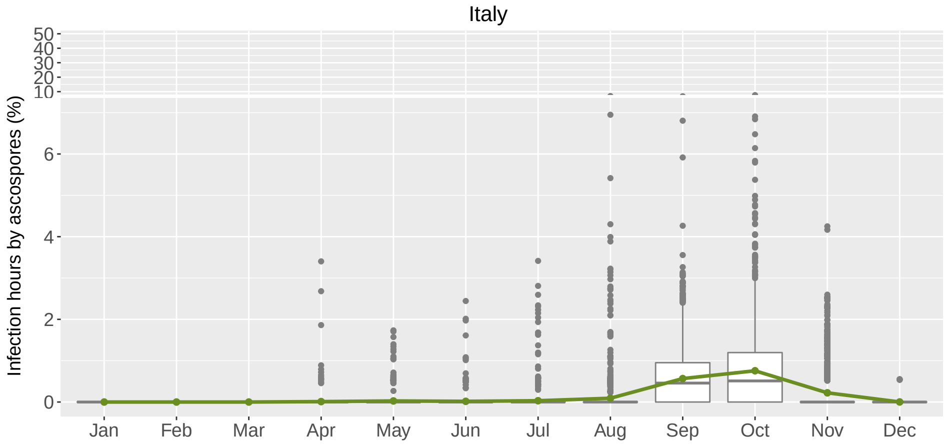

(c)

Figure SB3. Monthly percentage of hours with weather conditions suitable for successful infection events by *Phyllosticta citricarpa* ascospores (generic infection model for foliar fungal pathogens by Magarey et al.[1], configuration scenario S1) for 9-km grid interpolated climatic data from 2009 to 2018. Box-and-whisker plots for the citrus-growing regions in (a) Portugal and Spain, (b) Israel, and (c) Italy. Outliers represented by grey dots, and green line denotes the mean trend.

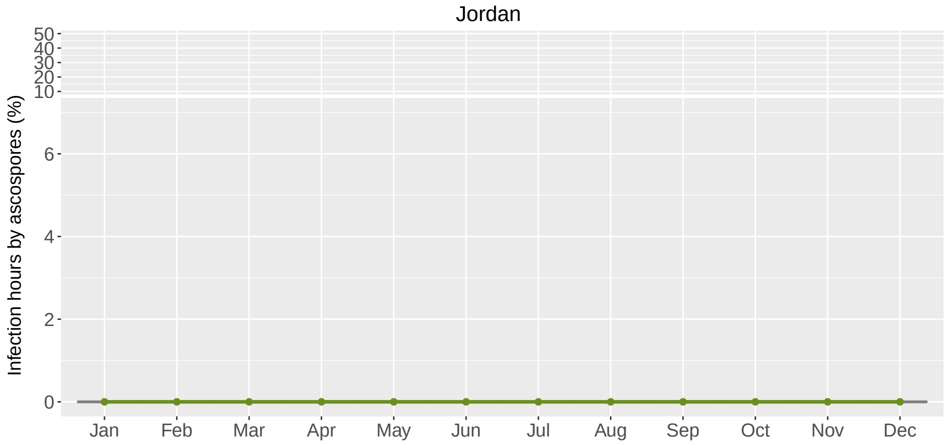

(a)

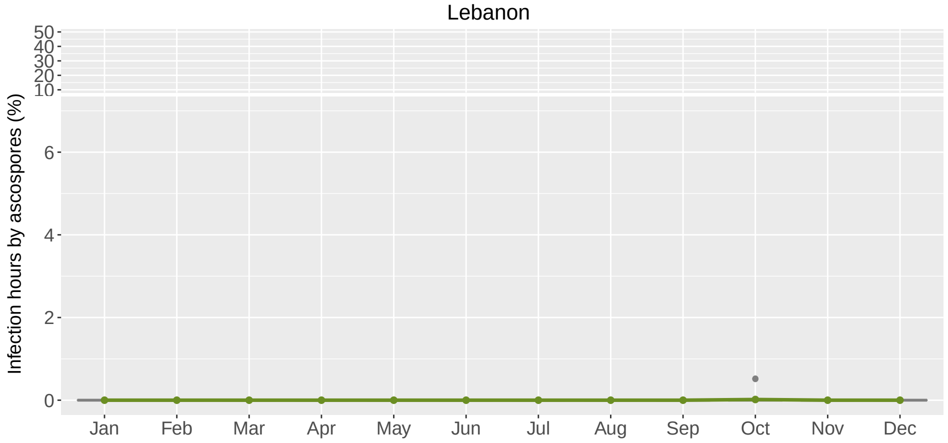

(b)

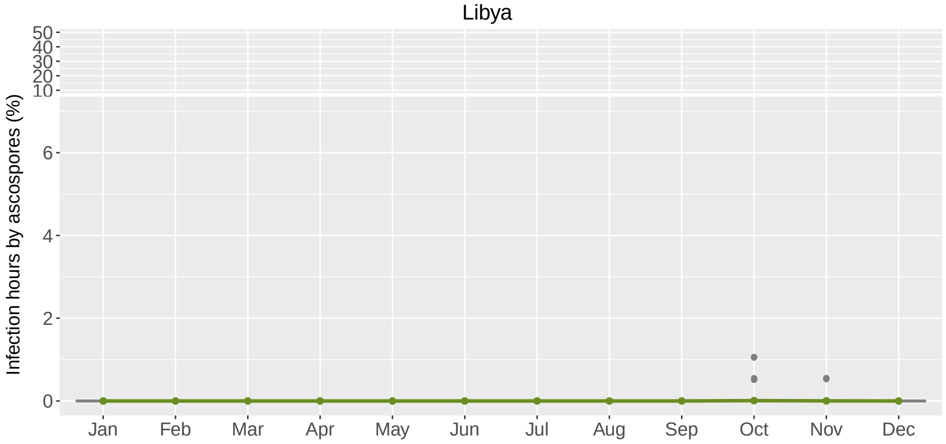

(c)

Figure SB4. Monthly percentage of hours with weather conditions suitable for successful infection events by *Phyllosticta citricarpa* ascospores (generic infection model for foliar fungal pathogens by Magarey et al.[1], configuration scenario S1) for 9-km grid interpolated climatic data from 2009 to 2018. Box-and-whisker plots for the citrus-growing regions in (a) Jordan, (b) Lebanon, and (c) Libya. Outliers represented by grey dots, and green line denotes the mean trend.

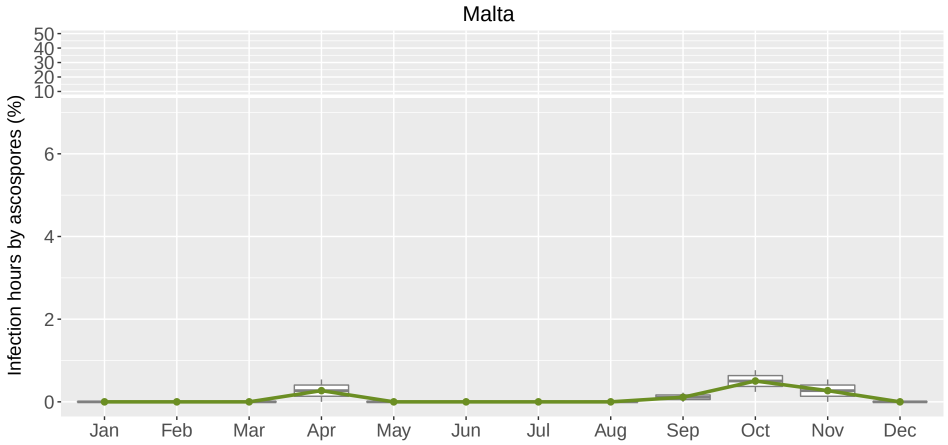

(a)

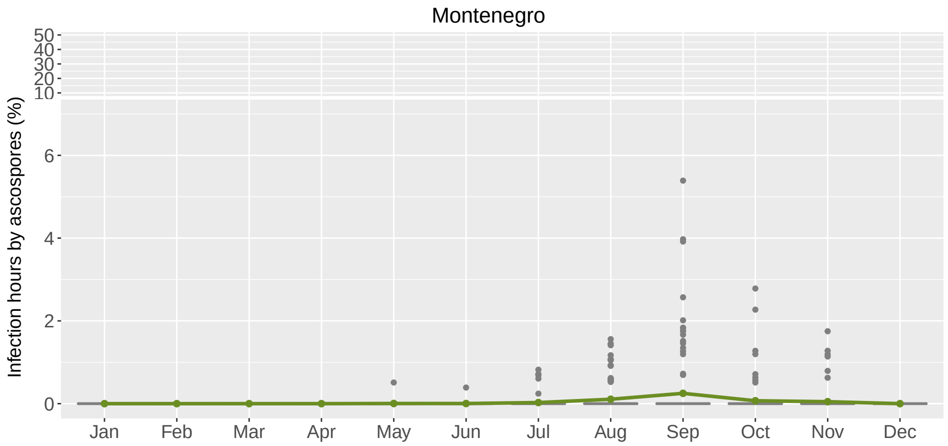

(b)

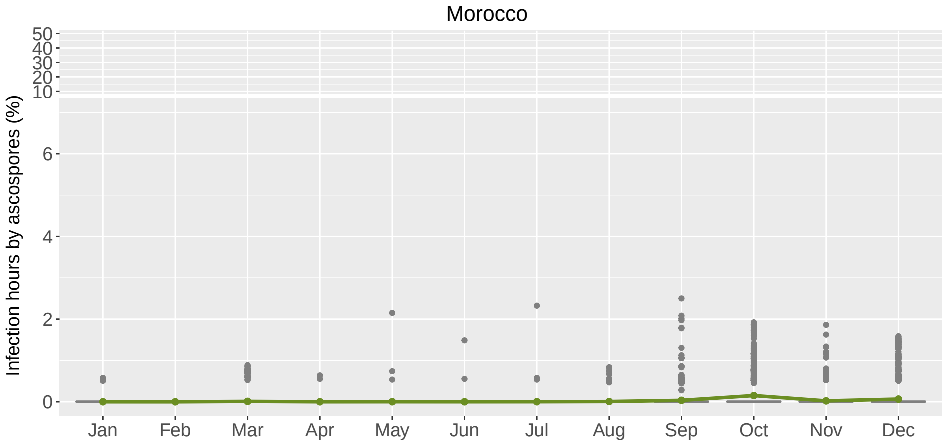

(c)

Figure SB5. Monthly percentage of hours with weather conditions suitable for successful infection events by *Phyllosticta citricarpa* ascospores (generic infection model for foliar fungal pathogens by Magarey et al.[1], configuration scenario S1) for 9-km grid interpolated climatic data from 2009 to 2018. Box-and-whisker plots for the citrus-growing regions in (a) Malta, (b) Montenegro, and (c) Morocco. Outliers represented by grey dots, and green line denotes the mean trend.

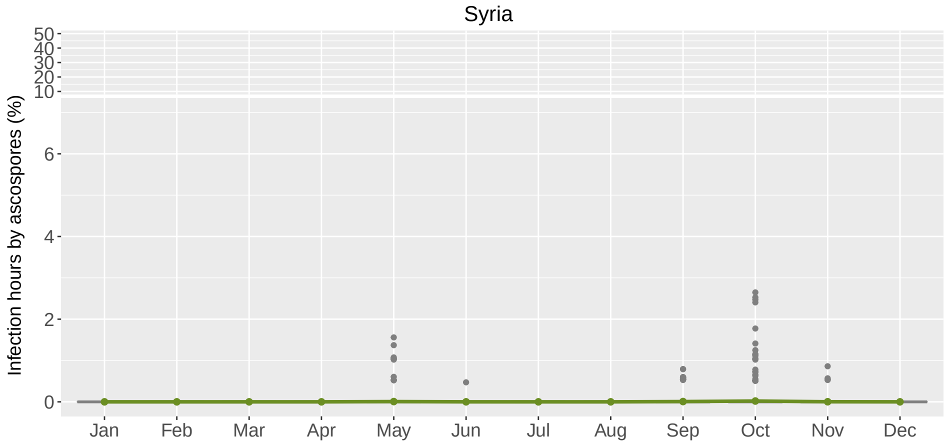

(a)

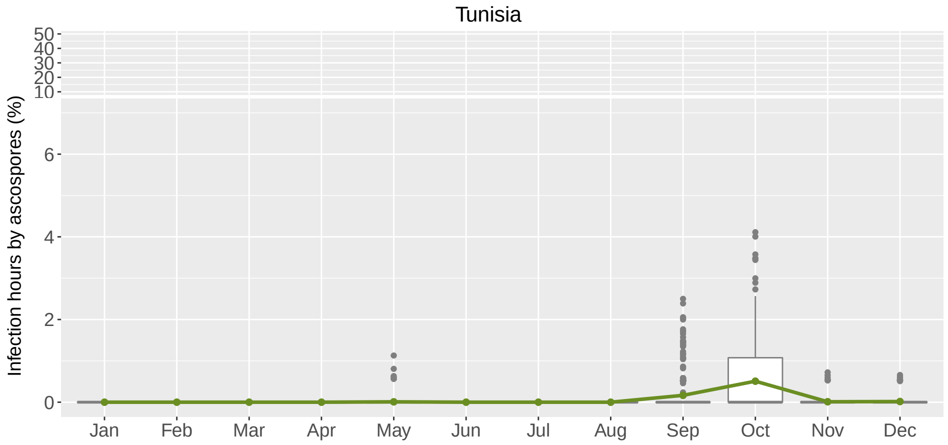

(b)

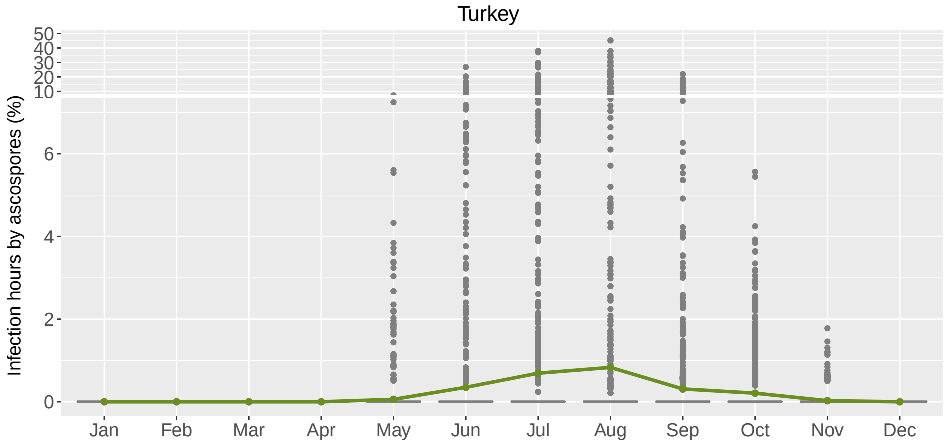

(c)

Figure SB6. Monthly percentage of hours with weather conditions suitable for successful infection events by *Phyllosticta citricarpa* ascospores (generic infection model for foliar fungal pathogens by Magarey et al.[1], configuration scenario S1) for 9-km grid interpolated climatic data from 2009 to 2018. Box-and-whisker plots for the citrus-growing regions in (a) Syria, (b) Tunisia, and (c) Turkey. Outliers represented by grey dots, and green line denotes the mean trend.

Pycnidiospore infection dynamics

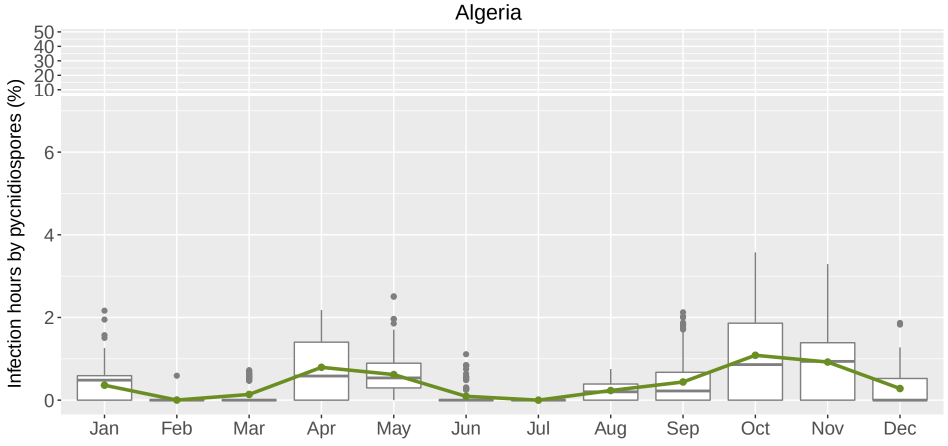

(a)

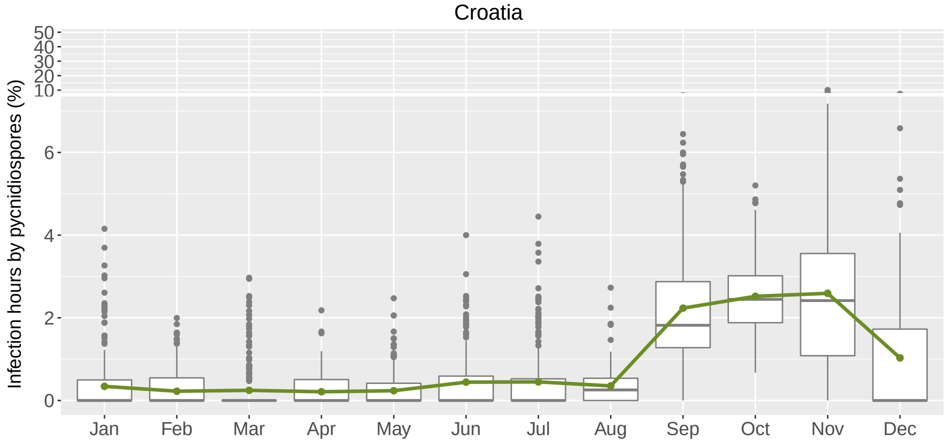

(b)

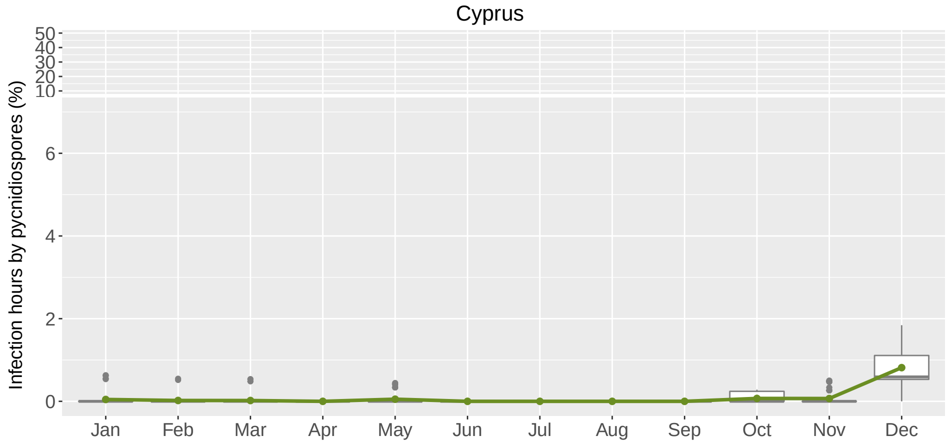

(c)

Figure SB7. Monthly percentage of hours with weather conditions suitable for successful infection events by *Phyllosticta citricarpa* pycnidiospores (generic infection model for foliar fungal pathogens by Magarey et al.[1], configuration scenario S1) for 9-km grid interpolated climatic data from 2009 to 2018. Box-and-whisker plots for the citrus-growing regions in (a) Algeria, (b) Croatia, and (c) Cyprus. Outliers represented by grey dots, and green line denotes the mean trend.

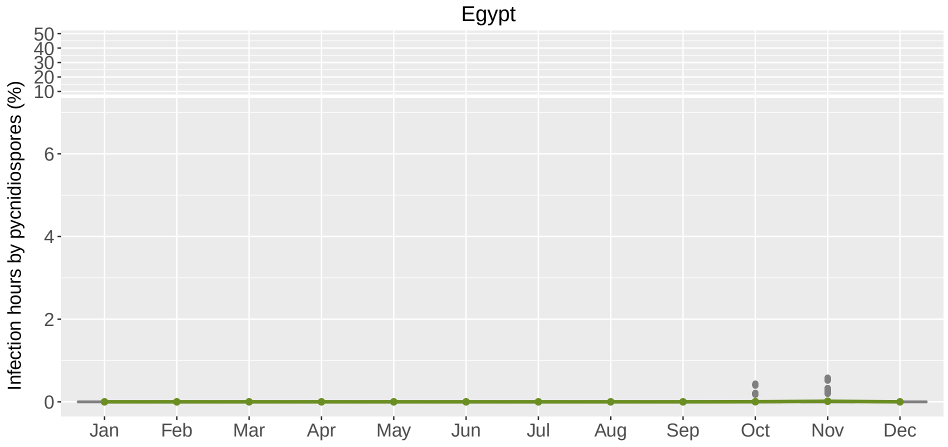

(a)

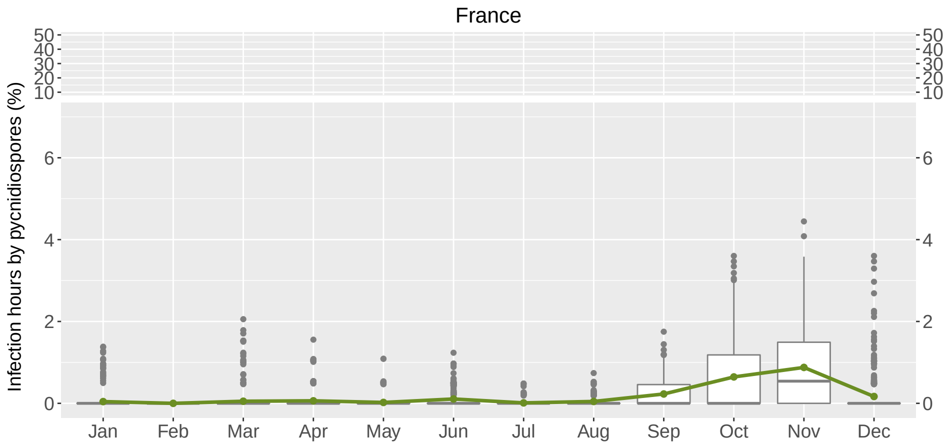

(b)

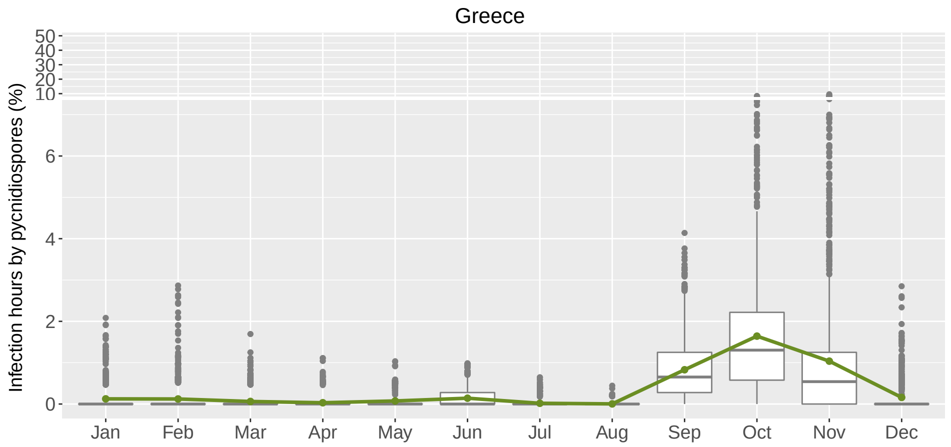

(c)

Figure SB8. Monthly percentage of hours with weather conditions suitable for successful infection events by *Phyllosticta citricarpa* pycnidiospores (generic infection model for foliar fungal pathogens by Magarey et al.[1], configuration scenario S1) for 9-km grid interpolated climatic data from 2009 to 2018. Box-and-whisker plots for the citrus-growing regions in (a) Egypt, (b) France, and (c) Greece. Outliers represented by grey dots, and green line denotes the mean trend.

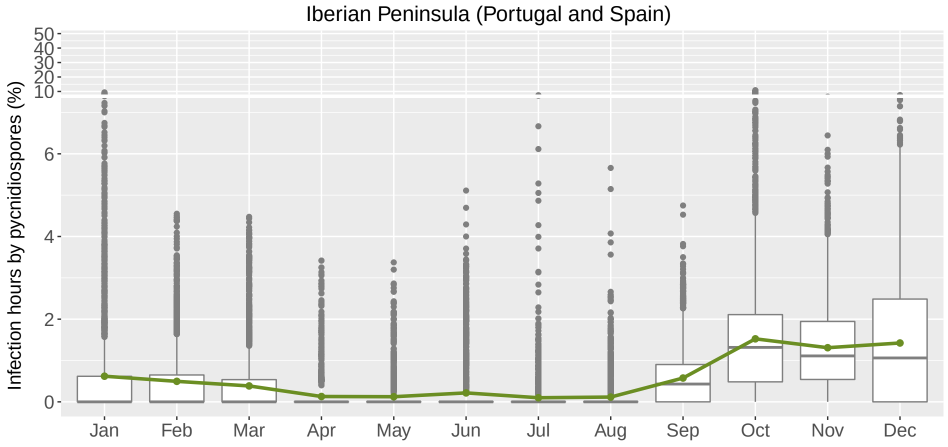

(a)

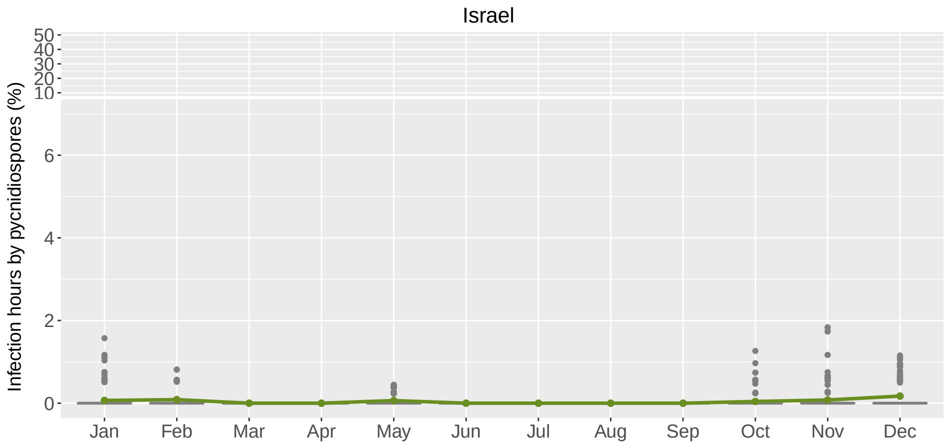

(b)

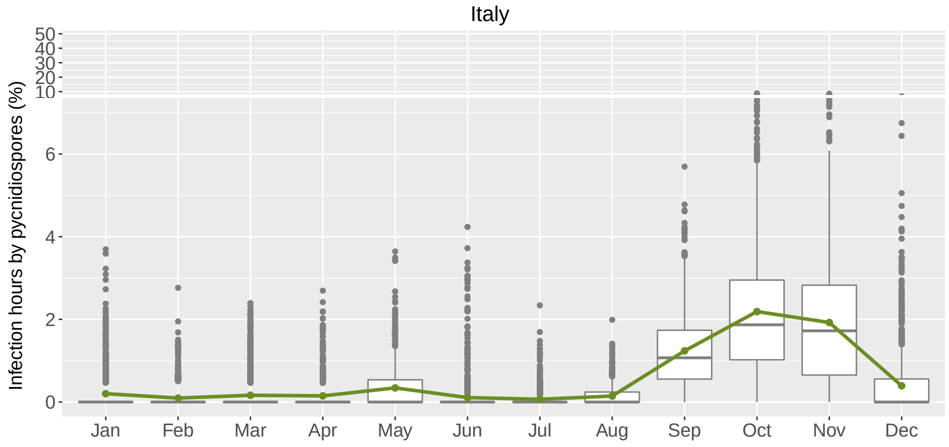

(c)

Figure SB9. Monthly percentage of hours with weather conditions suitable for successful infection events by *Phyllosticta citricarpa* pycnidiospores (generic infection model for foliar fungal pathogens by Magarey et al.[1], configuration scenario S1) for 9-km grid interpolated climatic data from 2009 to 2018. Box-and-whisker plots for the citrus-growing regions in (a) Portugal and Spain, (b) Israel, and (c) Italy. Outliers represented by grey dots, and green line denotes the mean trend.

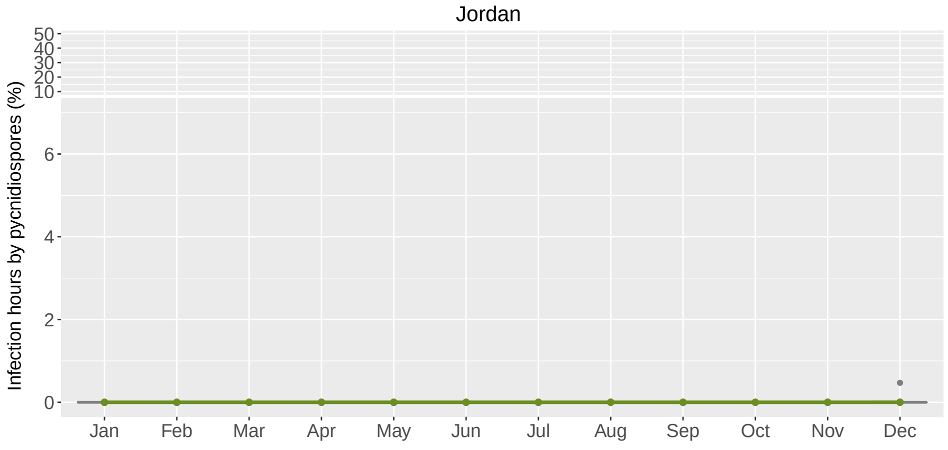

(a)

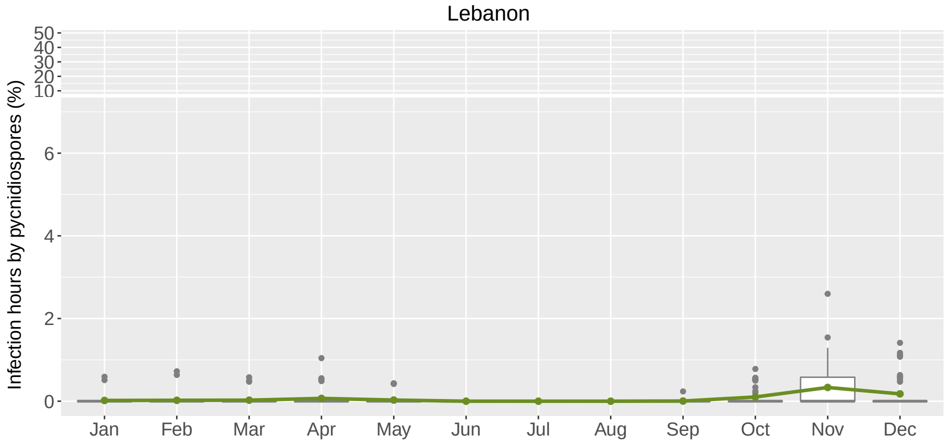

(b)

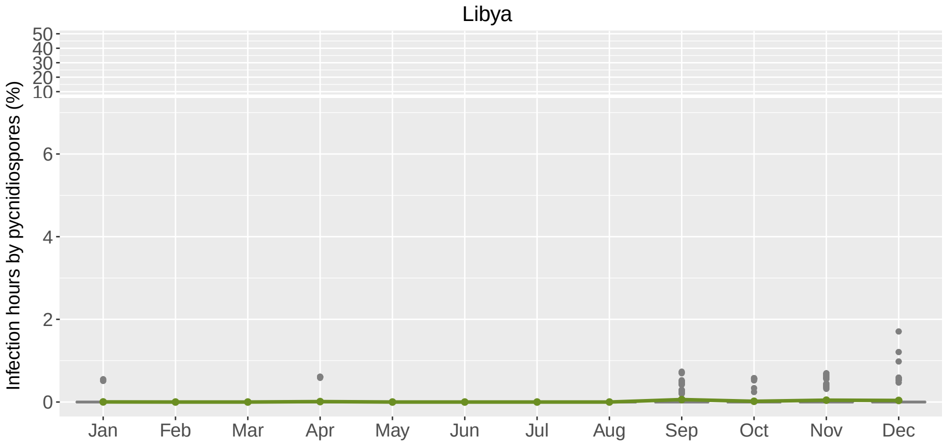

(c)

Figure SB10. Monthly percentage of hours with weather conditions suitable for successful infection events by *Phyllosticta citricarpa* pycnidiospores (generic infection model for foliar fungal pathogens by Magarey et al.[1], configuration scenario S1) for 9-km grid interpolated climatic data from 2009 to 2018. Box-and-whisker plots for the citrus-growing regions in (a) Jordan, (b) Lebanon, and (c) Libya. Outliers represented by grey dots, and green line denotes the mean trend.

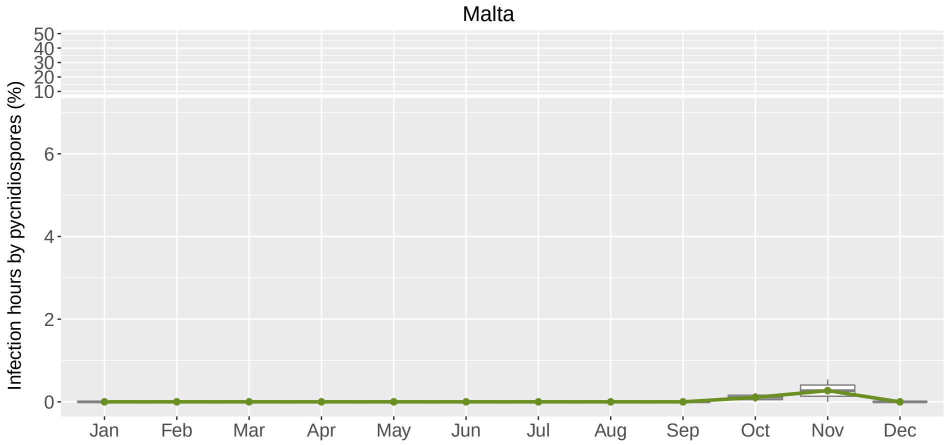

(a)

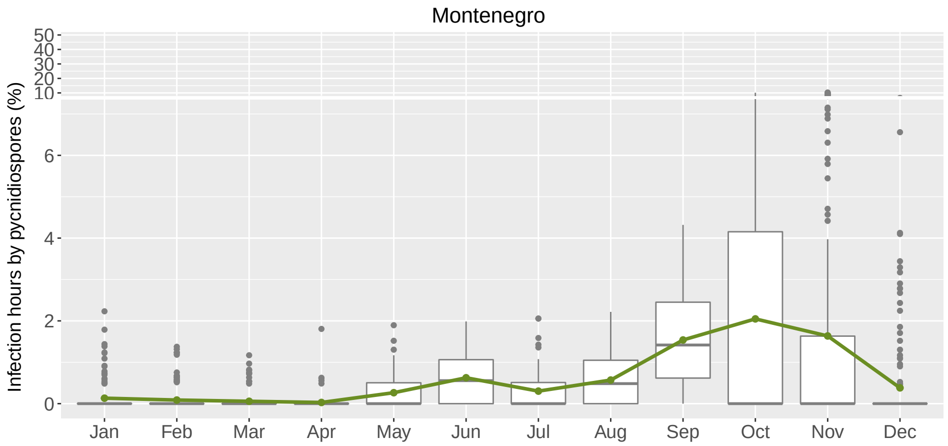

(b)

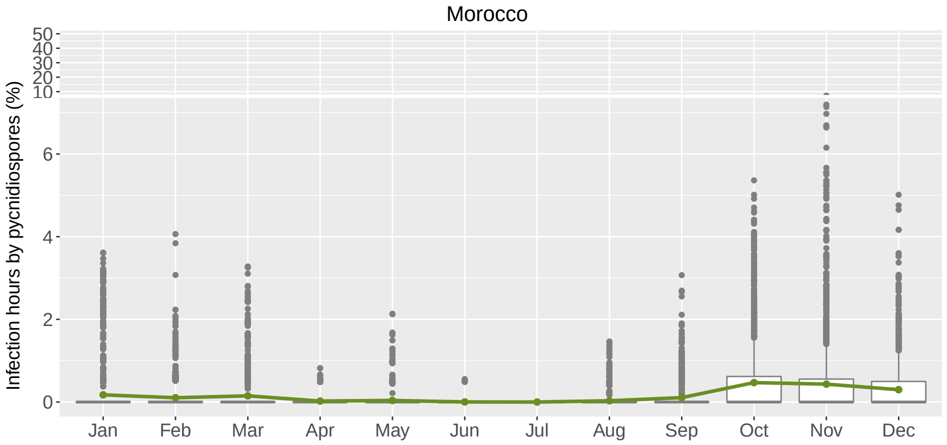

(c)

Figure SB11. Monthly percentage of hours with weather conditions suitable for successful infection events by *Phyllosticta citricarpa* pycnidiospores (generic infection model for foliar fungal pathogens by Magarey et al.[1], configuration scenario S1) for 9-km grid interpolated climatic data from 2009 to 2018. Box-and-whisker plots for the citrus-growing regions in (a) Malta, (b) Montenegro, and (c) Morocco. Outliers represented by grey dots, and green line denotes the mean trend.

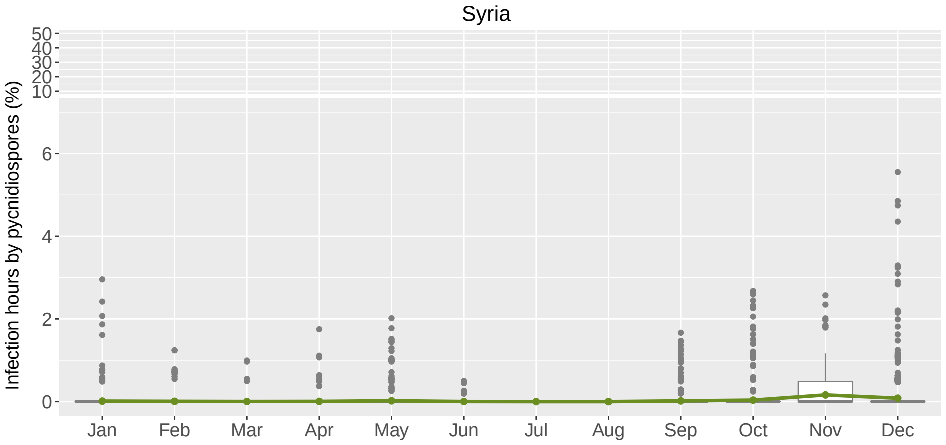

(a)

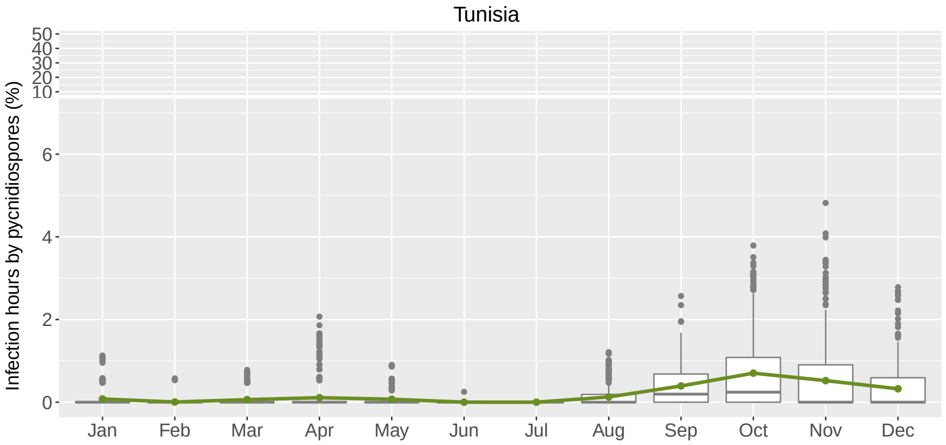

(b)

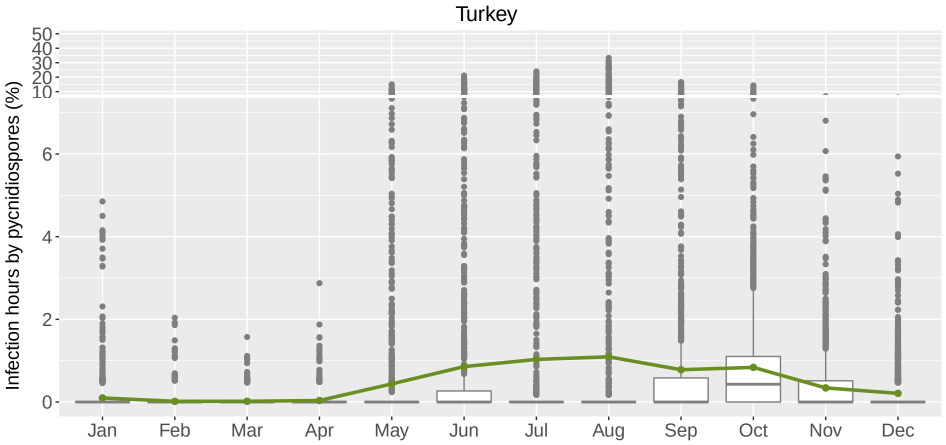

(c)

Figure SB12. Monthly percentage of hours with weather conditions suitable for successful infection events by *Phyllosticta citricarpa* pycnidiospores (generic infection model for foliar fungal pathogens by Magarey et al.[1], configuration scenario S1) for 9-km grid interpolated climatic data from 2009 to 2018. Box-and-whisker plots for the citrus-growing regions in (a) Syria, (b) Tunisia, and (c) Turkey. Outliers represented by grey dots, and green line denotes the mean trend.

References

1. Magarey, R., Sutton, T. & Thayer, C. A simple generic infection model for foliar fungal plant pathogens. *Phytopathology* **95**, 92–100 (2005).
