# Supplementary material for: Climate suitability of the Mediterranean Basin for citrus black spot disease (Phyllosticta citricarpa) based on a generic infection model
Source: Sci Rep. 2022 Nov 18;12:19876. doi: 10.1038/s41598-022-22775-z (PMC9674692; doi:10.1038/s41598-022-22775-z)
Supplement: Supplementary file 4 — Supplementary Information 4. [file 41598_2022_22775_MOESM4_ESM.pdf]

Supplementary Material D. Climate suitability of the Mediterranean Basin for citrus black spot disease (*Phyllosticta citricarpa*) based on a generic infection model. Figures of configuration scenario S1 for pycnidiospores by country

Anaïs Galvañ[1], Naima Boughalleb-M'Hamdi[2], Najwa Benfradj[2], Sabrine Mannai[2], Elena Lázaro[1,+], and Antonio Vicent[1,+,*]

[1]Institut Valencià d'Investigacions Agràries (IVIA), Centre de Protecció Vegetal i Biotecnologia, 46113 Moncada, Valencia, Spain
[2]Department of Biological Sciences and Plant Protection,Institut Supérieur Agronomique de Chott Mariem, LR21AGR05, University of Sousse,Chott Mariem,Sousse, 4042, Tunisia
[*]vicent_antciv@gva.es
[+]These authors contributed equally to this work

Supplementary Figures SD1 to SD18

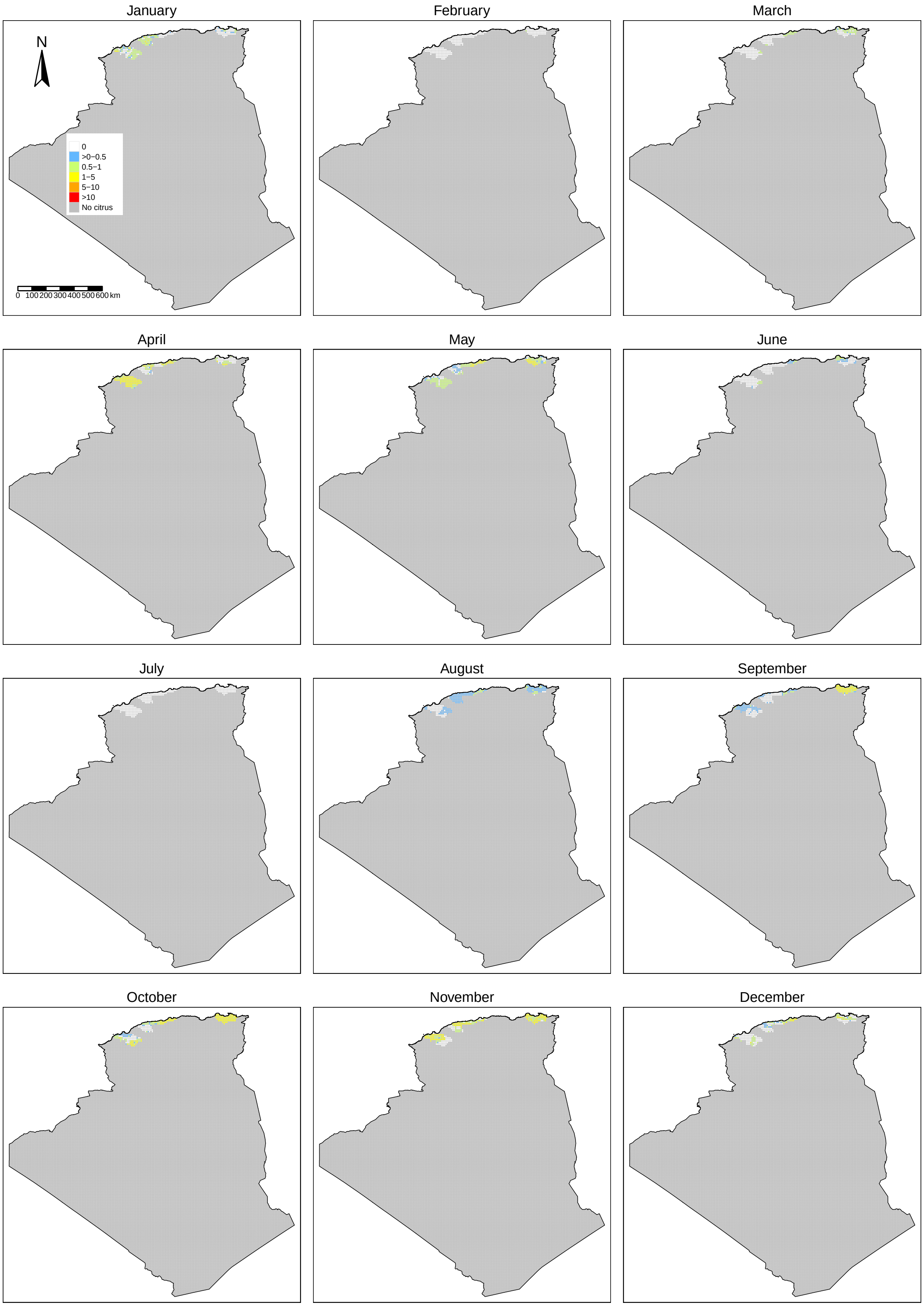

Figure SD1. Monthly percentage of hours (= 0 white,]0 – 0.5] blue,]0.5 – 1] green,]1 – 5] yellow,]5 – 10] orange, > 10 red) with suitable weather conditions for *Phyllosticta citricarpa* pycnidiospore infection (generic infection model for foliar fungal pathogens by Magarey et al.[1], configuration scenario S1) for the 9-km grid interpolated climatic data of the citrus-growing regions in Algeria from 2009 to 2018. Non citrus areas inside citrus-growing countries in dark-grey. The maps were created by the authors using the software R 3.6.0, https://www.R-project.org

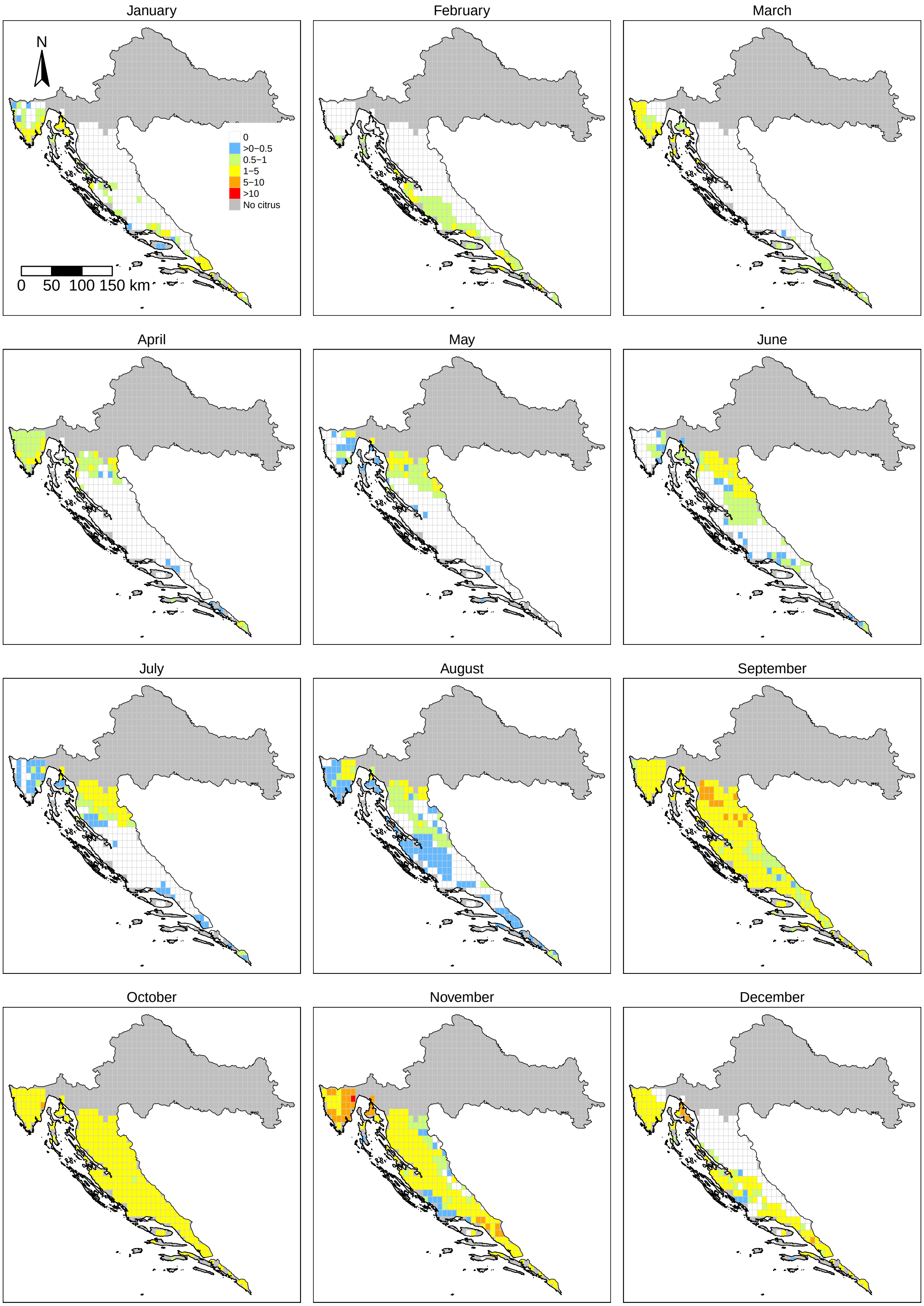

Figure SD2. Monthly percentage of hours (= 0 white,]0 − 0.5] blue,]0.5 − 1] green,]1 − 5] yellow,]5 − 10] orange, > 10 red) with suitable weather conditions for *Phyllosticta citricarpa* pycnidiospore infection (generic infection model for foliar fungal pathogens by Magarey et al.[1], configuration scenario S1) for the 9-km grid interpolated climatic data of the citrus-growing regions in Croatia from 2009 to 2018.Non citrus areas inside citrus-growing countries in dark-grey. The maps were created by the authors using the software R 3.6.0, https://www.R-project.org

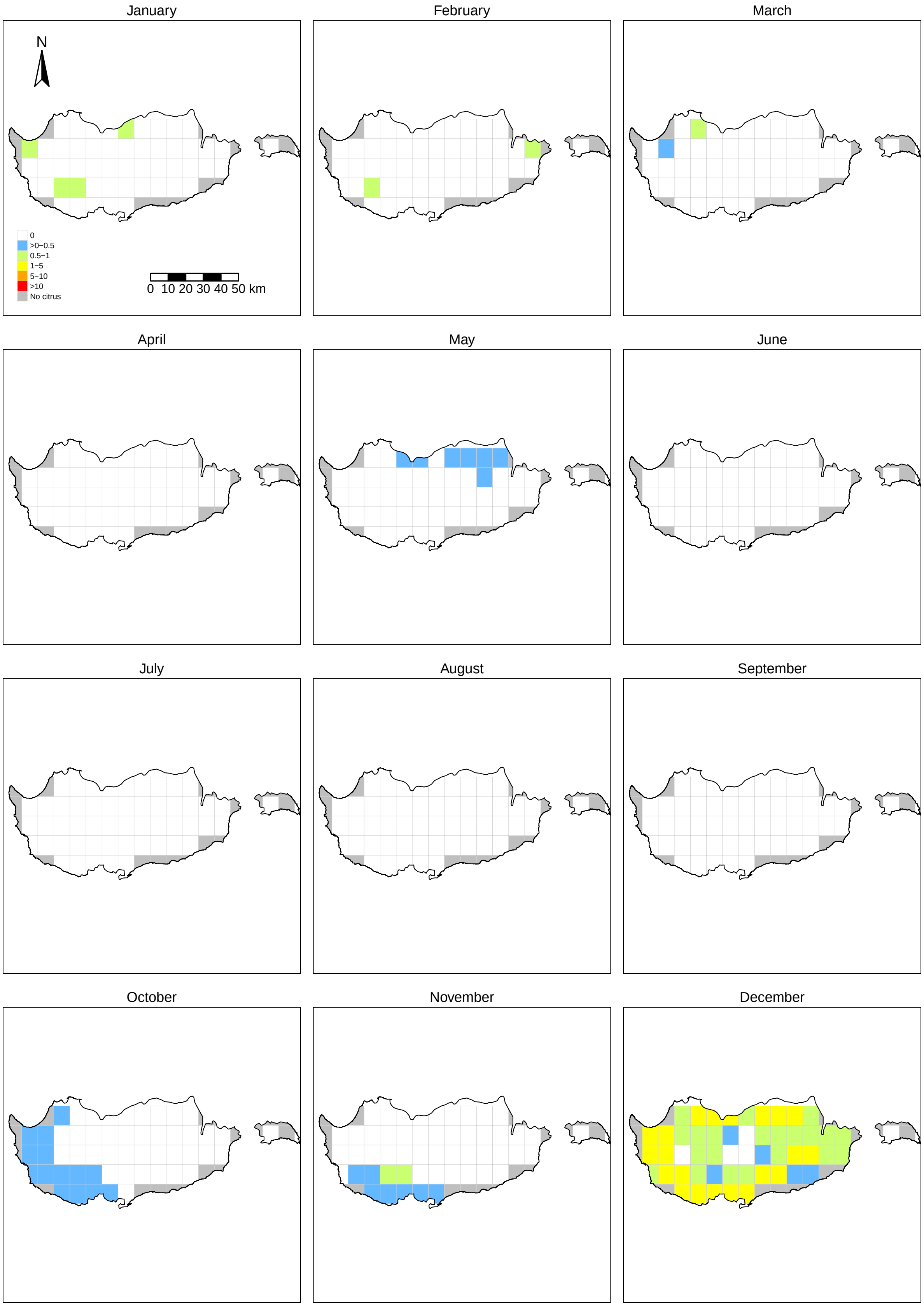

Figure SD3. Monthly percentage of hours (= 0 white,]0 – 0.5] blue,]0.5 – 1] green,]1 – 5] yellow,]5 – 10] orange, > 10 red) with suitable weather conditions for *Phyllosticta citricarpa* pycnidiospore infection (generic infection model for foliar fungal pathogens by Magarey et al.[1], configuration scenario S1) for the 9-km grid interpolated climatic data of the citrus-growing regions in Cyprus from 2009 to 2018. Non citrus areas inside citrus-growing countries in dark-grey. The maps were created by the authors using the software R 3.6.0, https://www.R-project.org

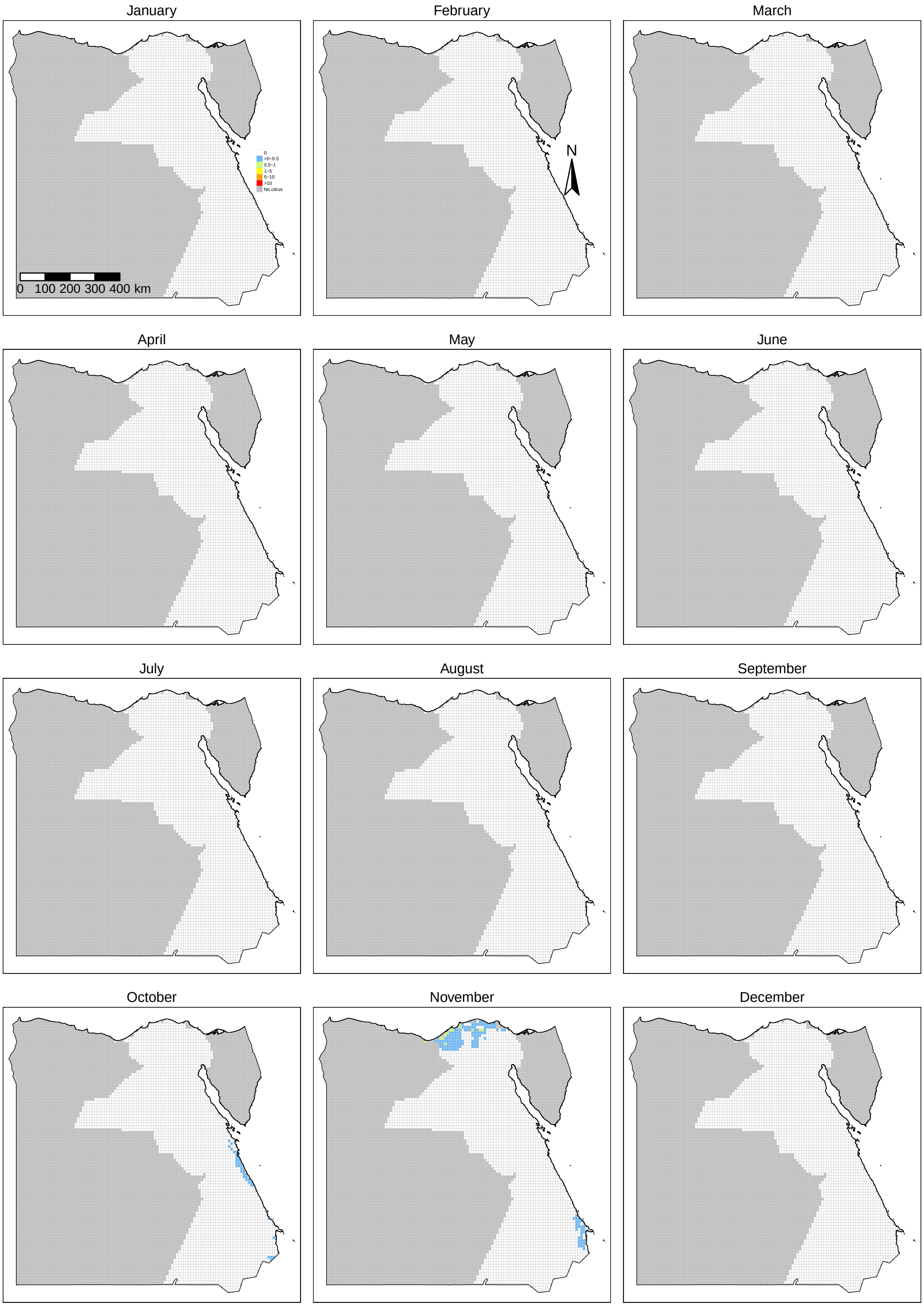

Figure SD4. Monthly percentage of hours (= 0 white,]0 − 0.5] blue,]0.5 − 1] green,]1 − 5] yellow,]5 − 10] orange, > 10 red) with suitable weather conditions for *Phyllosticta citricarpa* pycnidiospore infection (generic infection model for foliar fungal pathogens by Magarey et al.[1], configuration scenario S1) for the 9-km grid interpolated climatic data of the citrus-growing regions in Egypt from 2009 to 2018. Non citrus areas inside citrus-growing countries in dark-grey. The maps were created by the authors using the software `R` 3.6.0, `https://www.R-project.org`

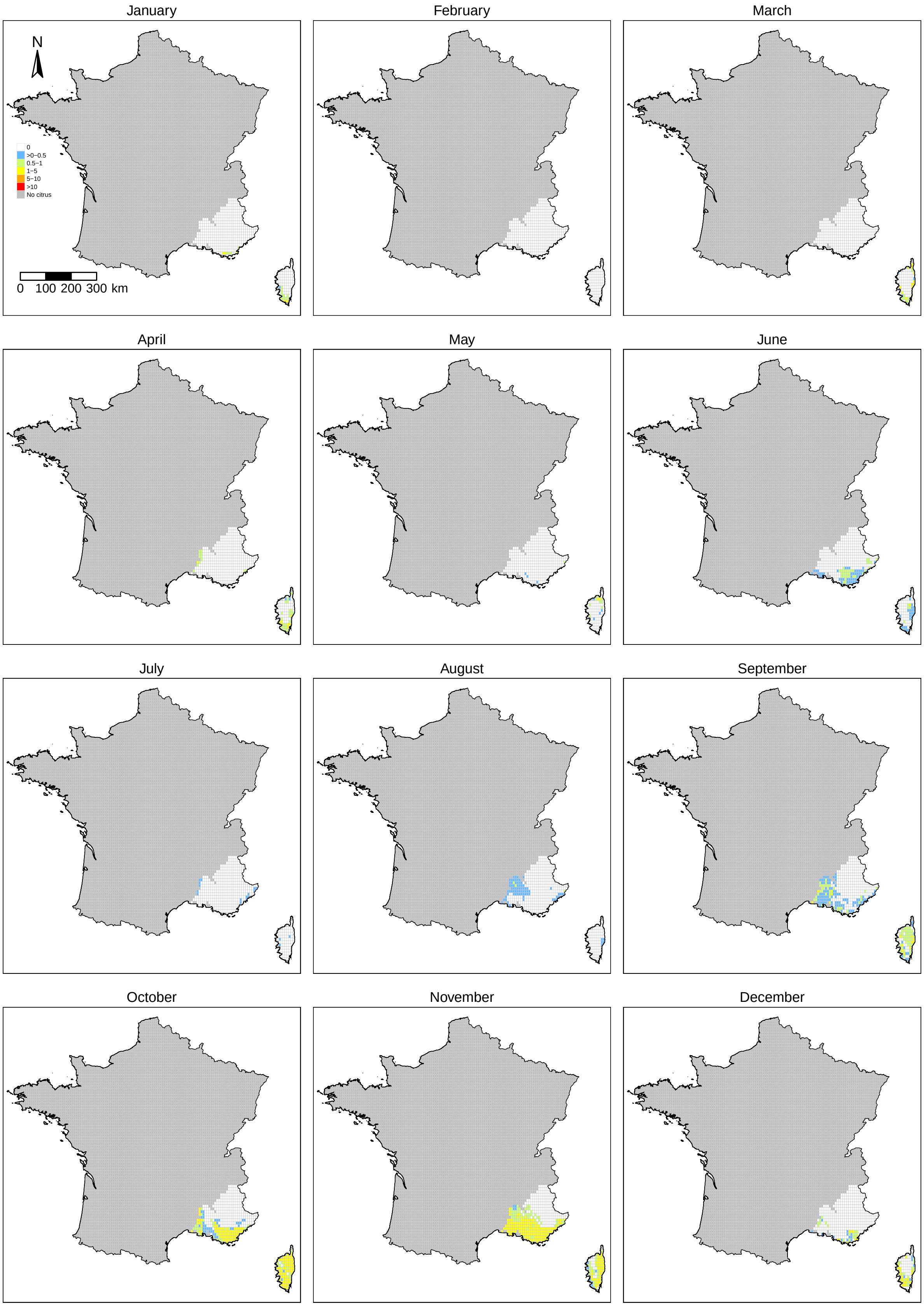

Figure SD5. Monthly percentage of hours ($= 0$ white, $]0 - 0.5]$ blue, $]0.5 - 1]$ green, $]1 - 5]$ yellow, $]5 - 10]$ orange, > 10 red) with suitable weather conditions for *Phyllosticta citricarpa* pycnidiospore infection (generic infection model for foliar fungal pathogens by Magarey et al.[1], configuration scenario S1) for the 9-km grid interpolated climatic data of the citrus-growing regions in France from 2009 to 2018.Non citrus areas inside citrus-growing countries in dark-grey. The maps were created by the authors using the software R 3.6.0, https://www.R-project.org

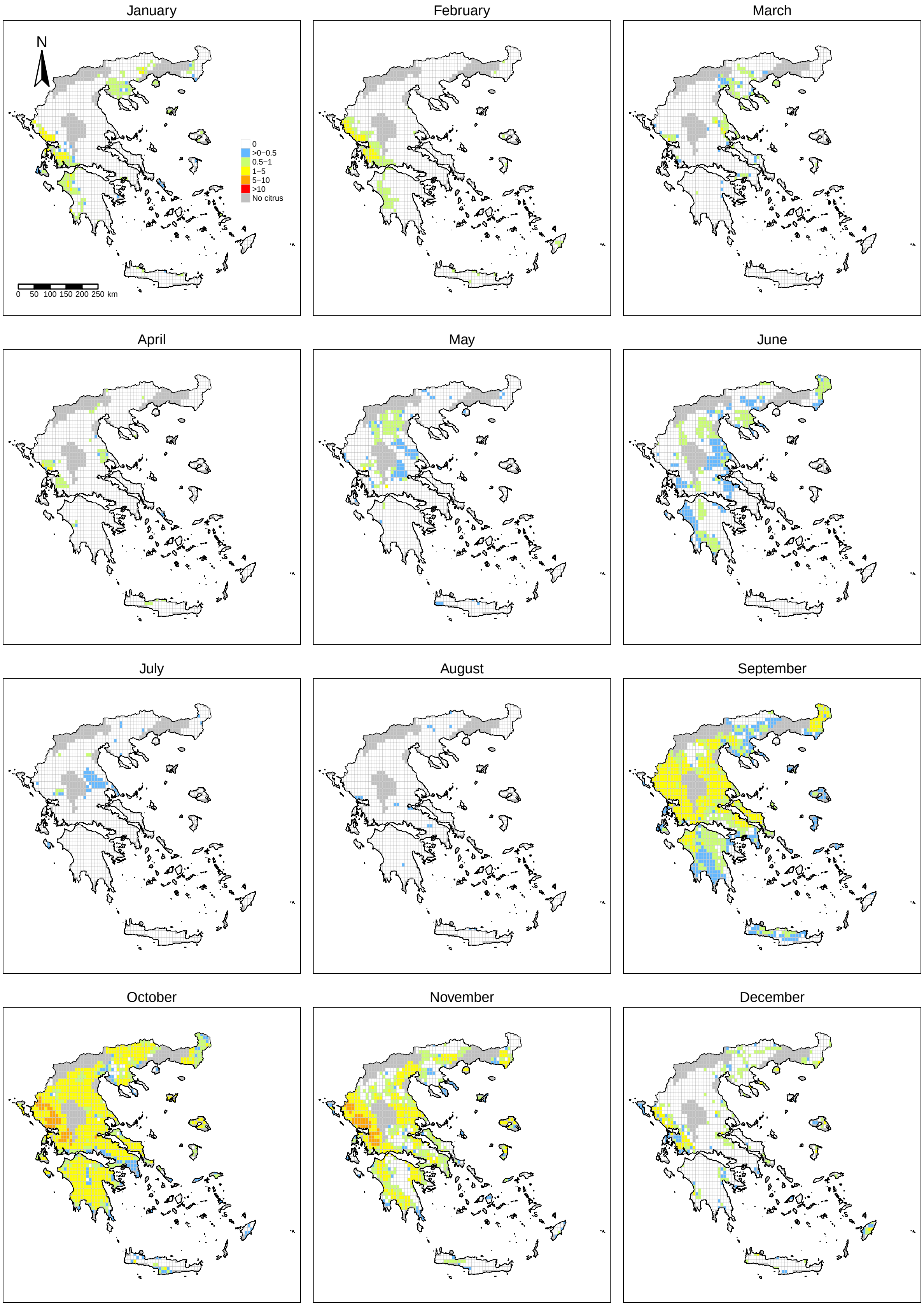

Figure SD6. Monthly percentage of hours($= 0$ white, $]0-0.5]$ blue, $]0.5-1]$ green, $]1-5]$ yellow, $]5-10]$ orange, > 10 red) with suitable weather conditions for *Phyllosticta citricarpa* pycnidiospore infection (generic infection model for foliar fungal pathogens by Magarey et al.[1], configuration scenario S1) for the 9-km grid interpolated climatic data of the citrus-growing regions in Greece from 2009 to 2018.Non citrus areas inside citrus-growing countries in dark-grey. The maps were created by the authors using the software R 3.6.0, https://www.R-project.org

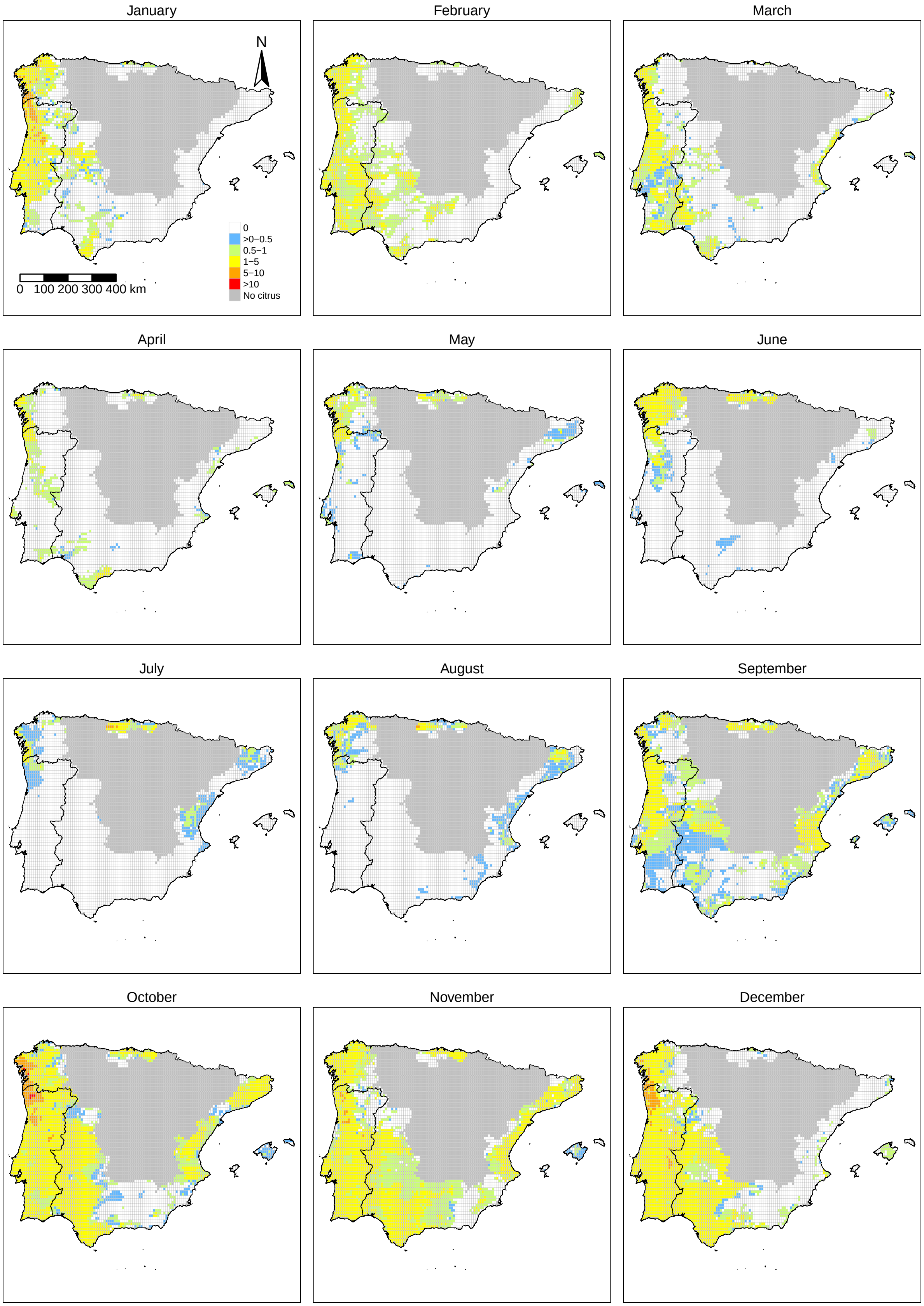

Figure SD7. Monthly percentage of hours (= 0 white,]0 – 0.5] blue,]0.5 – 1] green,]1 – 5] yellow,]5 – 10] orange, > 10 red) with suitable weather conditions for *Phyllosticta citricarpa* pycnidiospore infection (generic infection model for foliar fungal pathogens by Magarey et al.[1], configuration scenario S1) for the 9-km grid interpolated climatic data of the citrus-growing regions in Portugal and Spain from 2009 to 2018. Non citrus areas inside citrus-growing countries in dark-grey. The maps were created by the authors using the software R 3.6.0, https://www.R-project.org

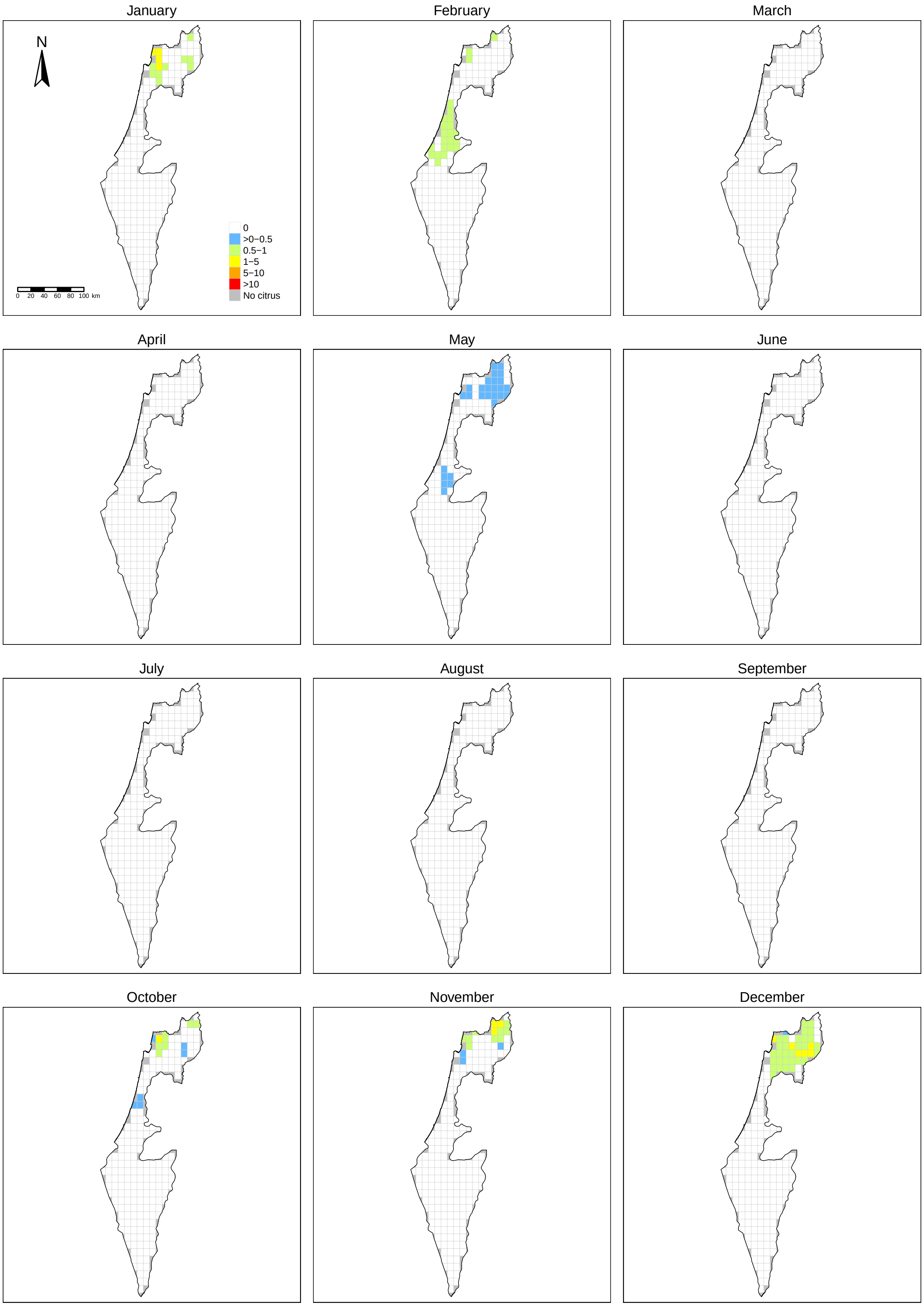

Figure SD8. Monthly percentage of hours (= 0 white,]0 − 0.5] blue,]0.5 − 1] green,]1 − 5] yellow,]5 − 10] orange, > 10 red) with suitable weather conditions for *Phyllosticta citricarpa* pycnidiospore infection (generic infection model for foliar fungal pathogens by Magarey et al.[1], configuration scenario S1) for the 9-km grid interpolated climatic data of the citrus-growing regions in Israel from 2009 to 2018.Non citrus areas inside citrus-growing countries in dark-grey. The maps were created by the authors using the software R 3.6.0, https://www.R-project.org

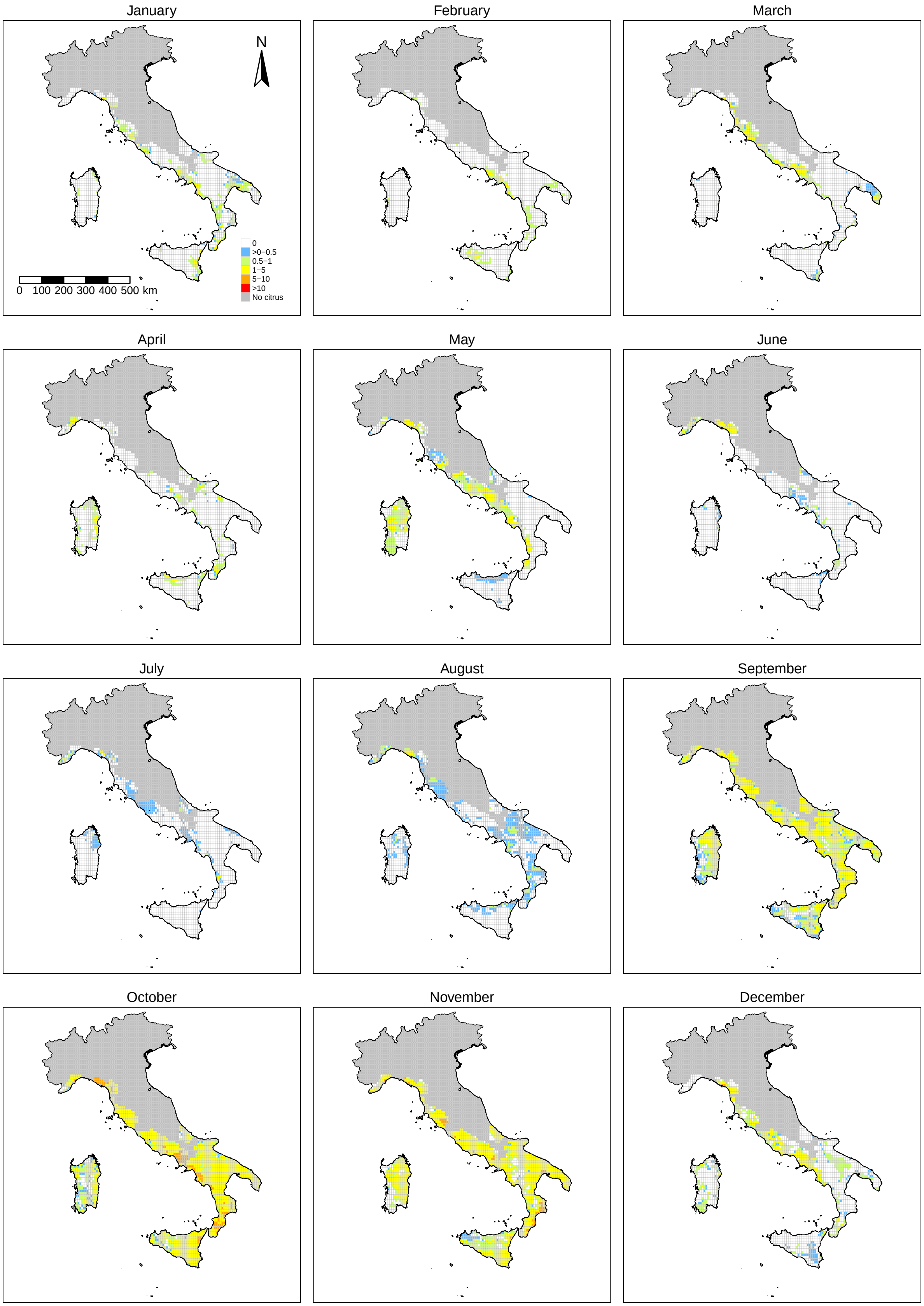

Figure SD9. Monthly percentage of hours (= 0 white,]0 − 0.5] blue,]0.5 − 1] green,]1 − 5] yellow,]5 − 10] orange, > 10 red) with suitable weather conditions for *Phyllosticta citricarpa* pycnidiospore infection (generic infection model for foliar fungal pathogens by Magarey et al.[1], configuration scenario S1) for the 9-km grid interpolated climatic data of the citrus-growing regions in Italy from 2009 to 2018.Non citrus areas inside citrus-growing countries in dark-grey. The maps were created by the authors using the software R 3.6.0, https://www.R-project.org

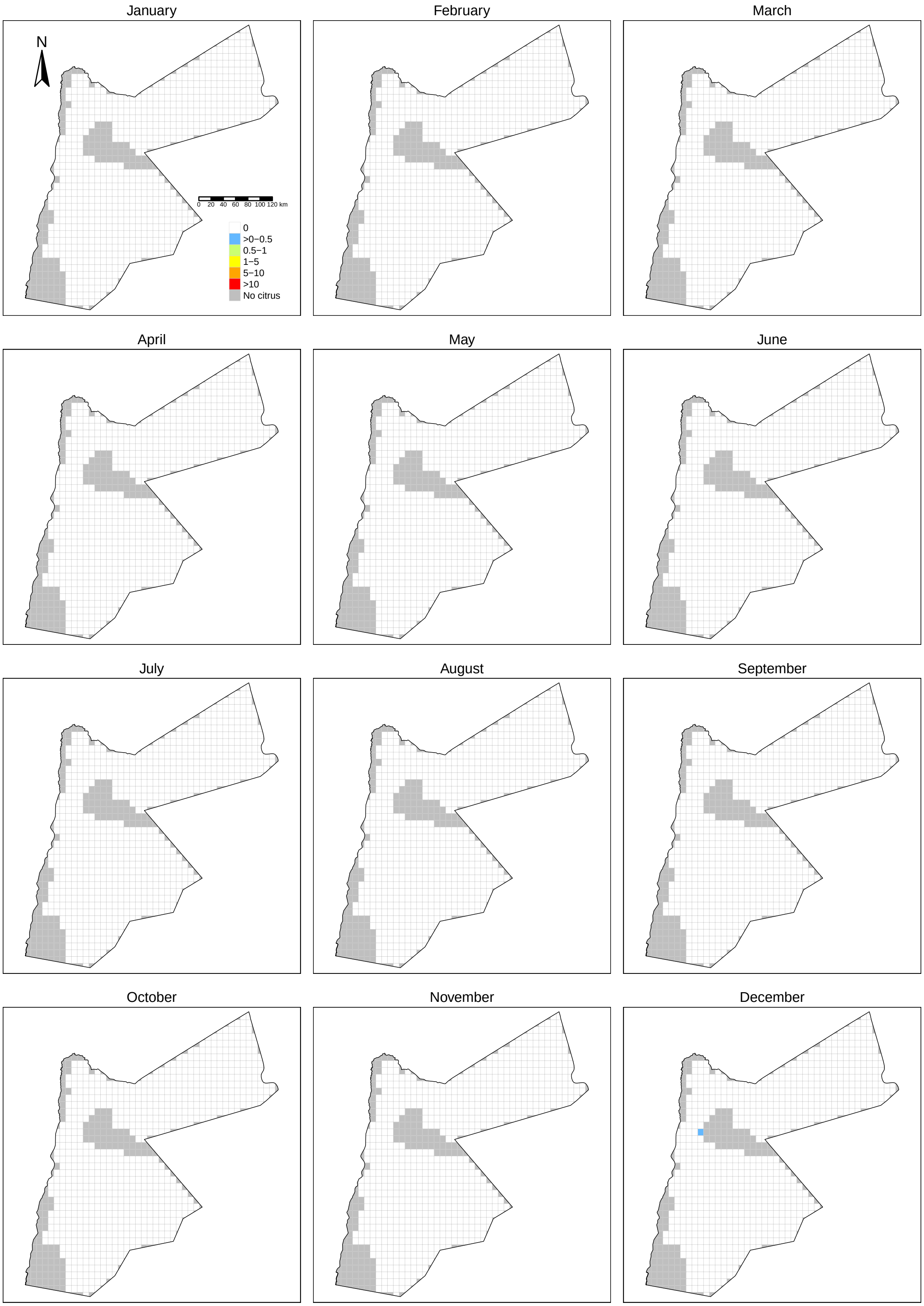

Figure SD10. Monthly percentage of hours (= 0 white,]0 − 0.5] blue,]0.5 − 1] green,]1 − 5] yellow,]5 − 10] orange, > 10 red) with suitable weather conditions for *Phyllosticta citricarpa* pycnidiospore infection (generic infection model for foliar fungal pathogens by Magarey et al.[1], configuration scenario S1) for the 9-km grid interpolated climatic data of the citrus-growing regions in Jordan from 2009 to 2018. Non citrus areas inside citrus-growing countries in dark-grey. The maps were created by the authors using the software R 3.6.0, https://www.R-project.org

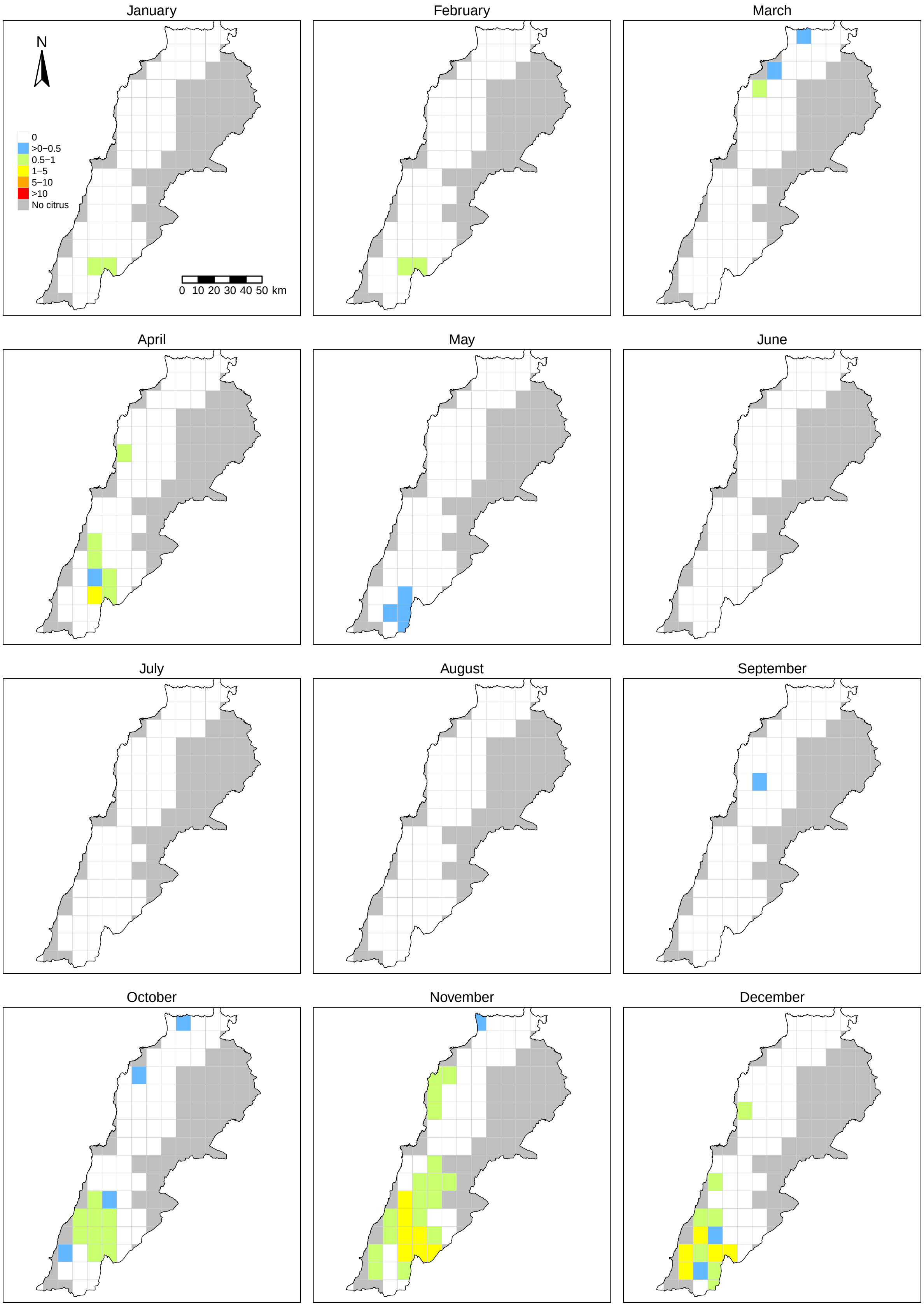

Figure SD11. Monthly percentage of hours (= 0 white,]0 − 0.5] blue,]0.5 − 1] green,]1 − 5] yellow,]5 − 10] orange, > 10 red) with suitable weather conditions for *Phyllosticta citricarpa* pycnidiospore infection (generic infection model for foliar fungal pathogens by Magarey et al.[1], configuration scenario S1) for the 9-km grid interpolated climatic data of the citrus-growing regions in Lebanon from 2009 to 2018. Non citrus areas inside citrus-growing countries in dark-grey. The maps were created by the authors using the software R 3.6.0, https://www.R-project.org

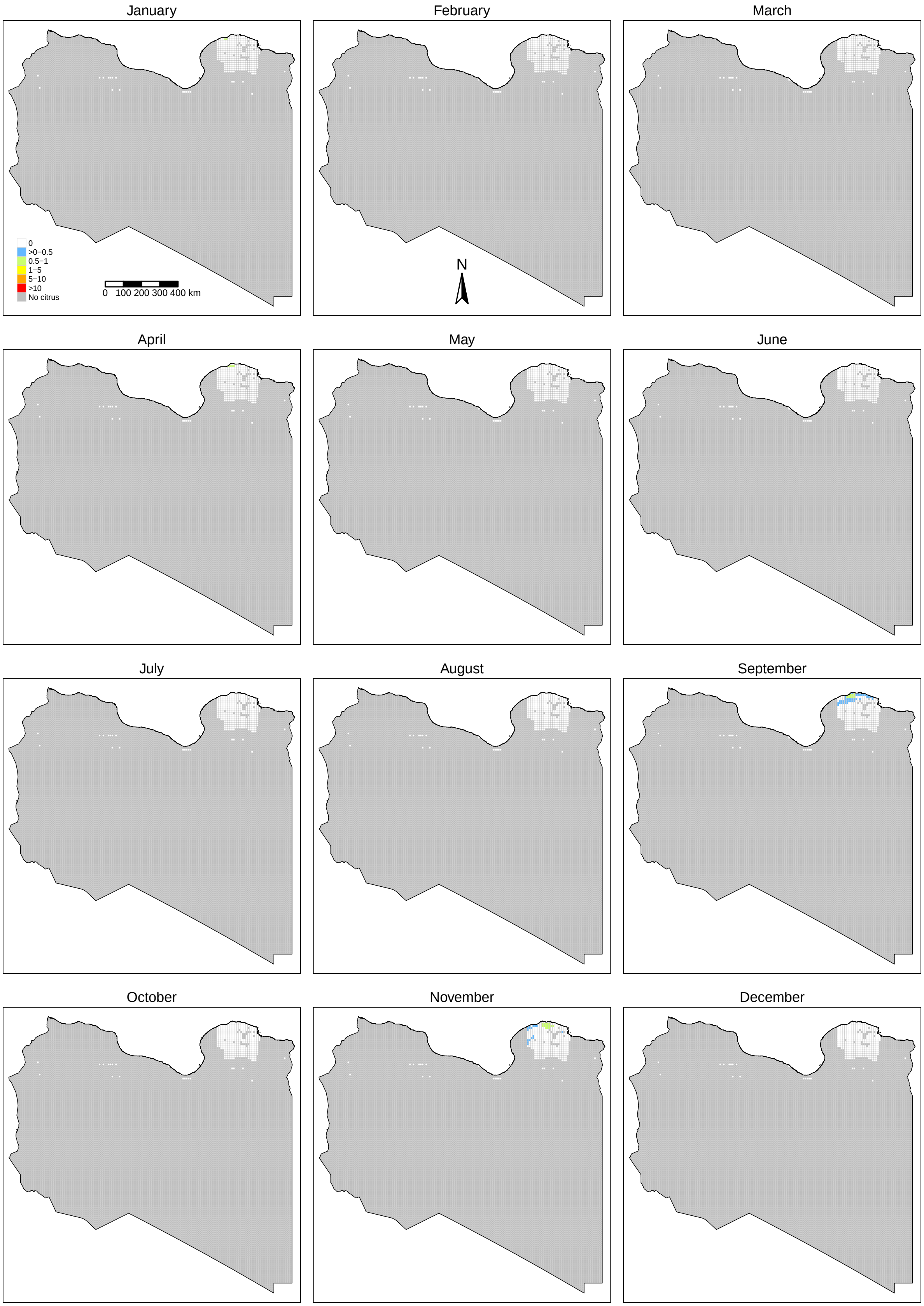

Figure SD12. Monthly percentage of hours (= 0 white,]0 − 0.5] blue,]0.5 − 1] green,]1 − 5] yellow,]5 − 10] orange, > 10 red) with suitable weather conditions for *Phyllosticta citricarpa* pycnidiospore infection (generic infection model for foliar fungal pathogens by Magarey et al.[1], configuration scenario S1) for the 9-km grid interpolated climatic data of the citrus-growing regions in Libya from 2009 to 2018. Non citrus areas inside citrus-growing countries in dark-grey. The maps were created by the authors using the software R 3.6.0, https://www.R-project.org

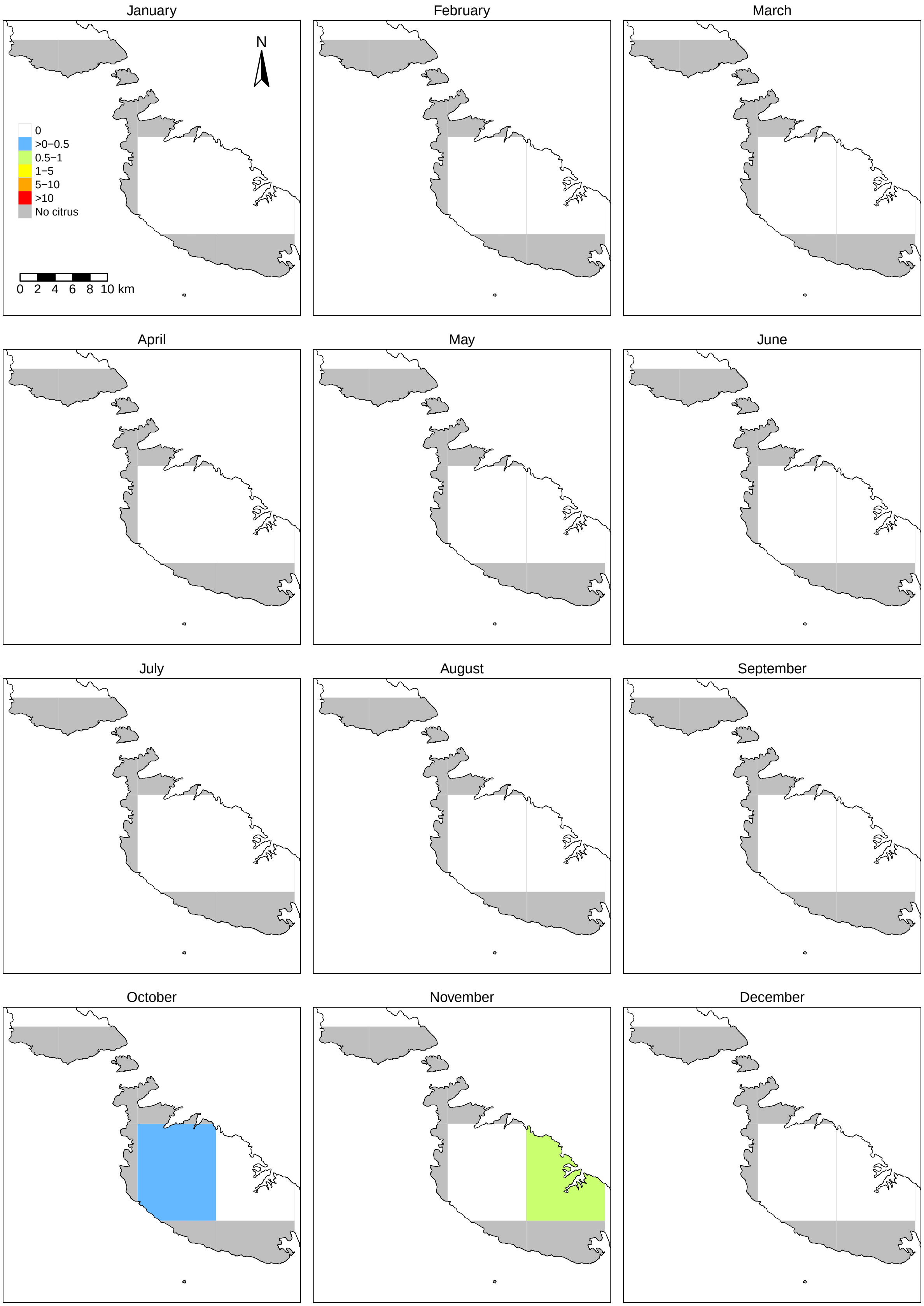

Figure SD13. Monthly percentage of hours (= 0 white,]0 – 0.5] blue,]0.5 – 1] green,]1 – 5] yellow,]5 – 10] orange, > 10 red) with suitable weather conditions for *Phyllosticta citricarpa* pycnidiospore infection (generic infection model for foliar fungal pathogens by Magarey et al.[1], configuration scenario S1) for the 9-km grid interpolated climatic data of the citrus-growing regions in Malta from 2009 to 2018.Non citrus areas inside citrus-growing countries in dark-grey. The maps were created by the authors using the software R 3.6.0, https://www.R-project.org

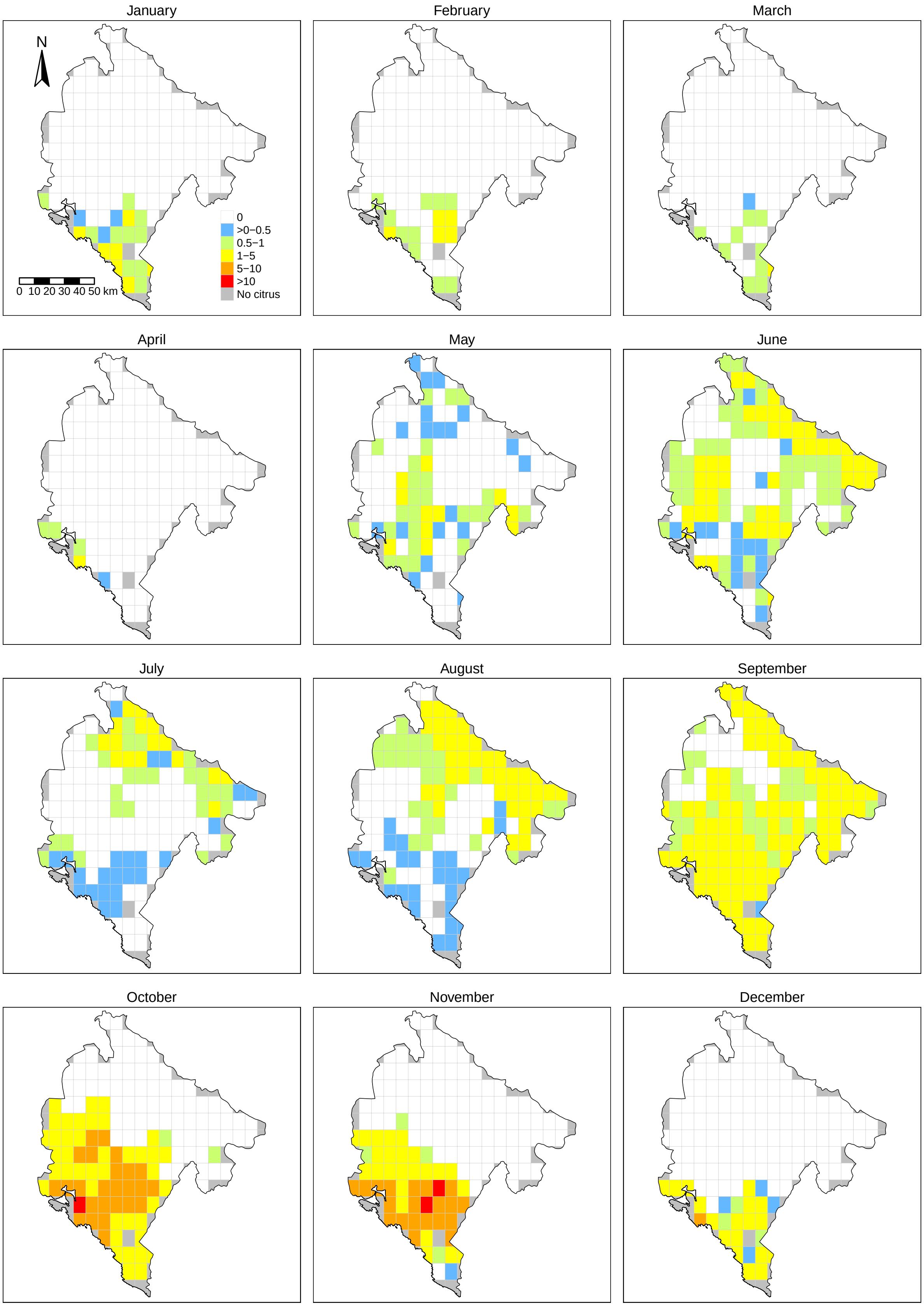

Figure SD14. Monthly percentage of hours (= 0 white,]0 − 0.5] blue,]0.5 − 1] green,]1 − 5] yellow,]5 − 10] orange, > 10 red) with suitable weather conditions for *Phyllosticta citricarpa* pycnidiospore infection (generic infection model for foliar fungal pathogens by Magarey et al.[1], configuration scenario S1) for the 9-km grid interpolated climatic data of the citrus-growing regions in Montenegro from 2009 to 2018. Non citrus areas inside citrus-growing countries in dark-grey. The maps were created by the authors using the software `R` 3.6.0, https://www.R-project.org

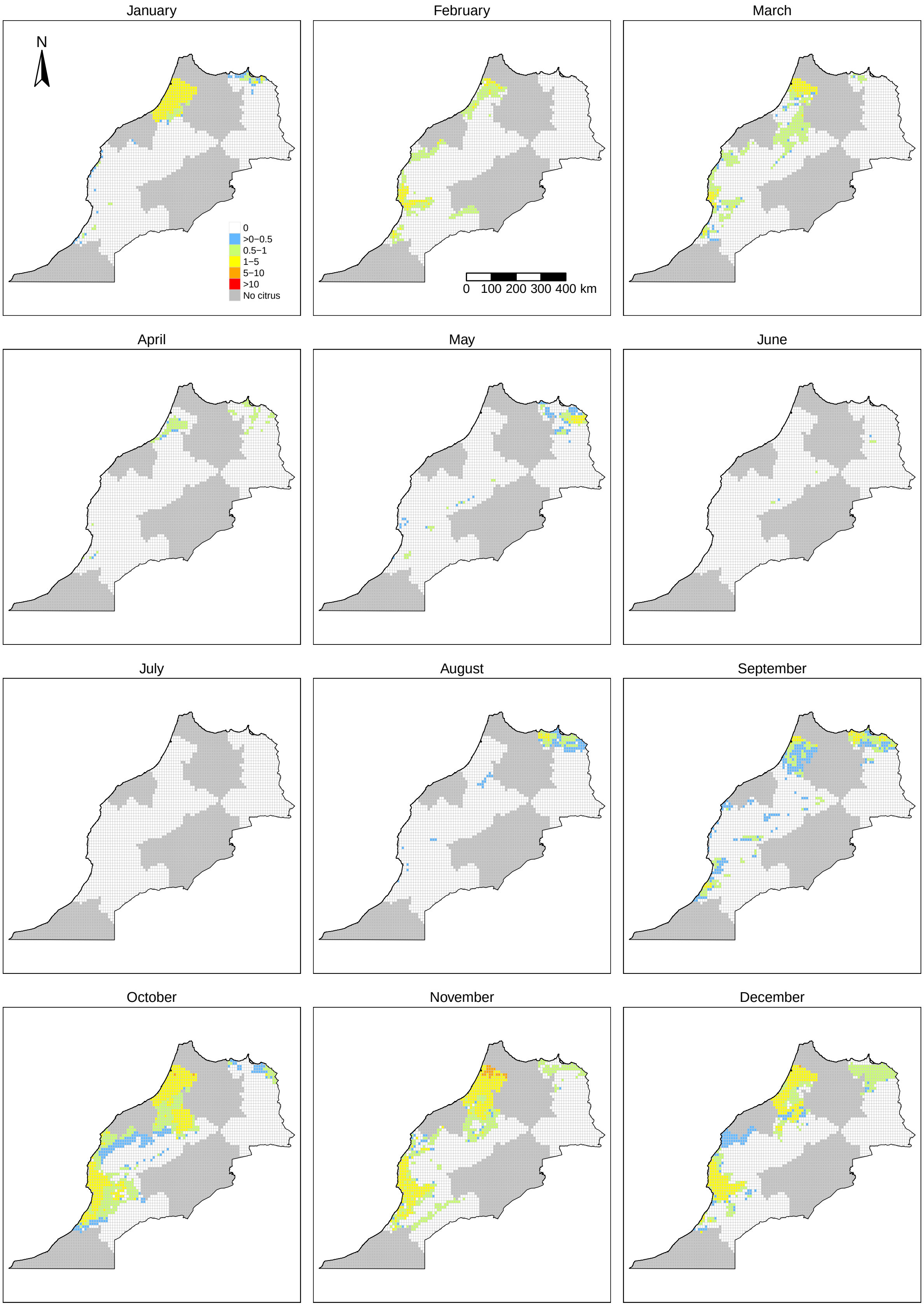

Figure SD15. Monthly percentage of hours (= 0 white,]0 – 0.5] blue,]0.5 – 1] green,]1 – 5] yellow,]5 – 10] orange, > 10 red) with suitable weather conditions for *Phyllosticta citricarpa* pycnidiospore infection (generic infection model for foliar fungal pathogens by Magarey et al.[1], configuration scenario S1) for the 9-km grid interpolated climatic data of the citrus-growing regions in Morocco from 2009 to 2018. Non citrus areas inside citrus-growing countries in dark-grey. The maps were created by the authors using the software R 3.6.0, https://www.R-project.org

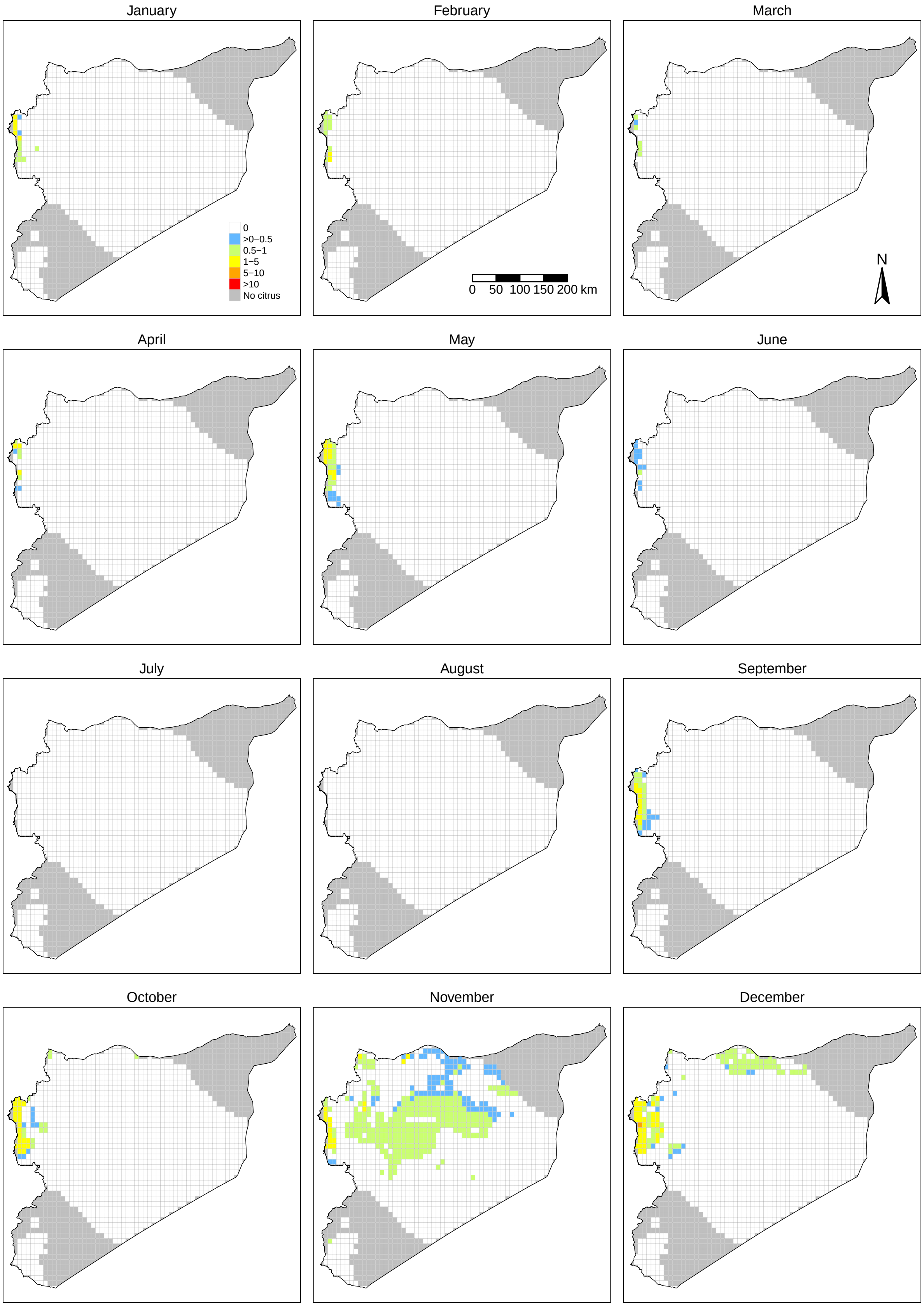

Figure SD16. Monthly percentage of hours($= 0$ white, $]0-0.5]$ blue, $]0.5-1]$ green, $]1-5]$ yellow, $]5-10]$ orange, > 10 red) with suitable weather conditions for *Phyllosticta citricarpa* pycnidiospore infection (generic infection model for foliar fungal pathogens by Magarey et al.[1], configuration scenario S1) for the 9-km grid interpolated climatic data of the citrus-growing regions in Syria from 2009 to 2018. Non citrus areas inside citrus-growing countries in dark-grey. The maps were created by the authors using the software R 3.6.0, https://www.R-project.org

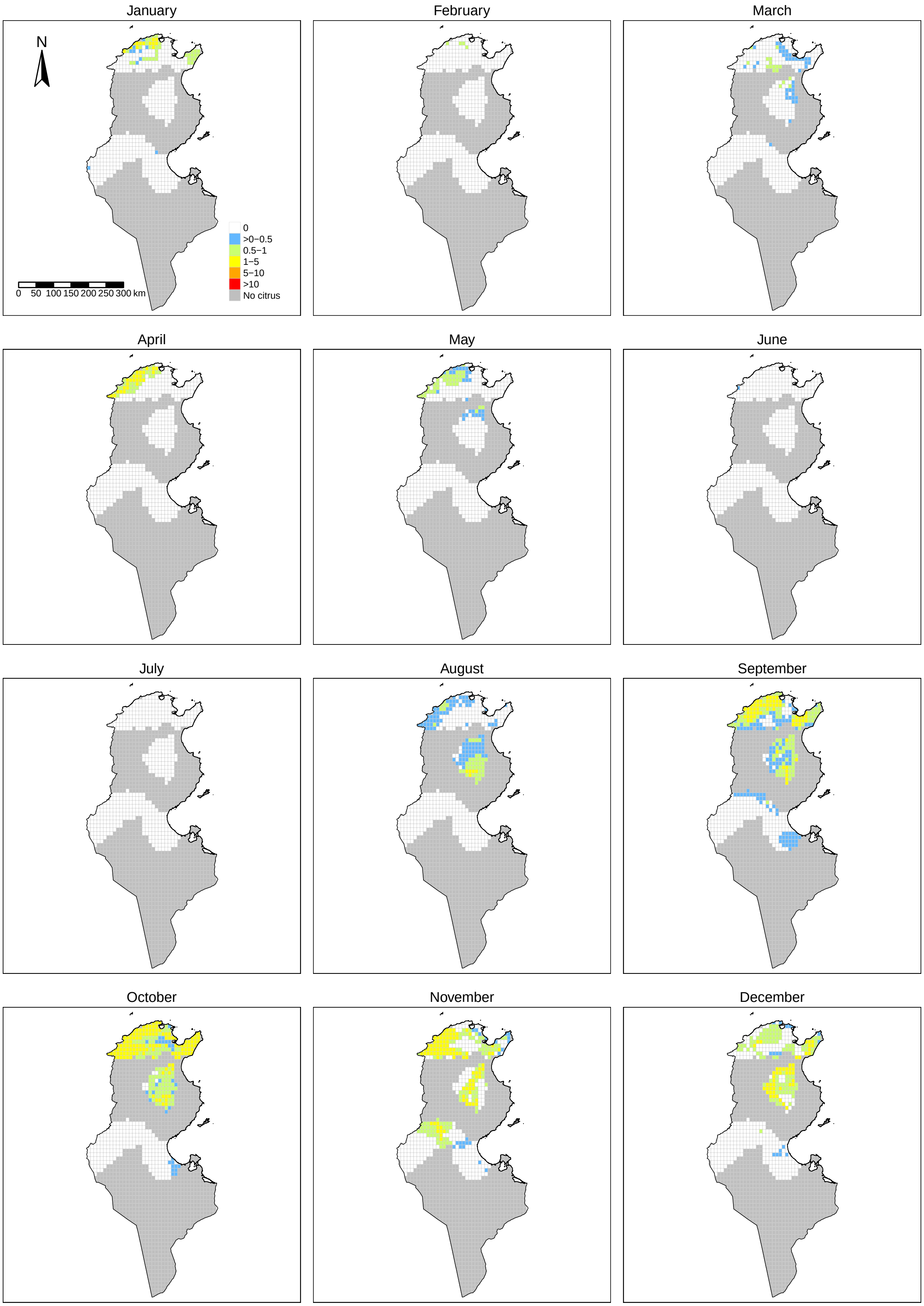

Figure SD17. Monthly percentage of hours (= 0 white,]0 − 0.5] blue,]0.5 − 1] green,]1 − 5] yellow,]5 − 10] orange, > 10 red) with suitable weather conditions for *Phyllosticta citricarpa* pycnidiospore infection (generic infection model for foliar fungal pathogens by Magarey et al.[1], configuration scenario S1) for the 9-km grid interpolated climatic data of the citrus-growing regions in Tunisia from 2009 to 2018. Non citrus areas inside citrus-growing countries in dark-grey. The maps were created by the authors using the software R 3.6.0, https://www.R-project.org

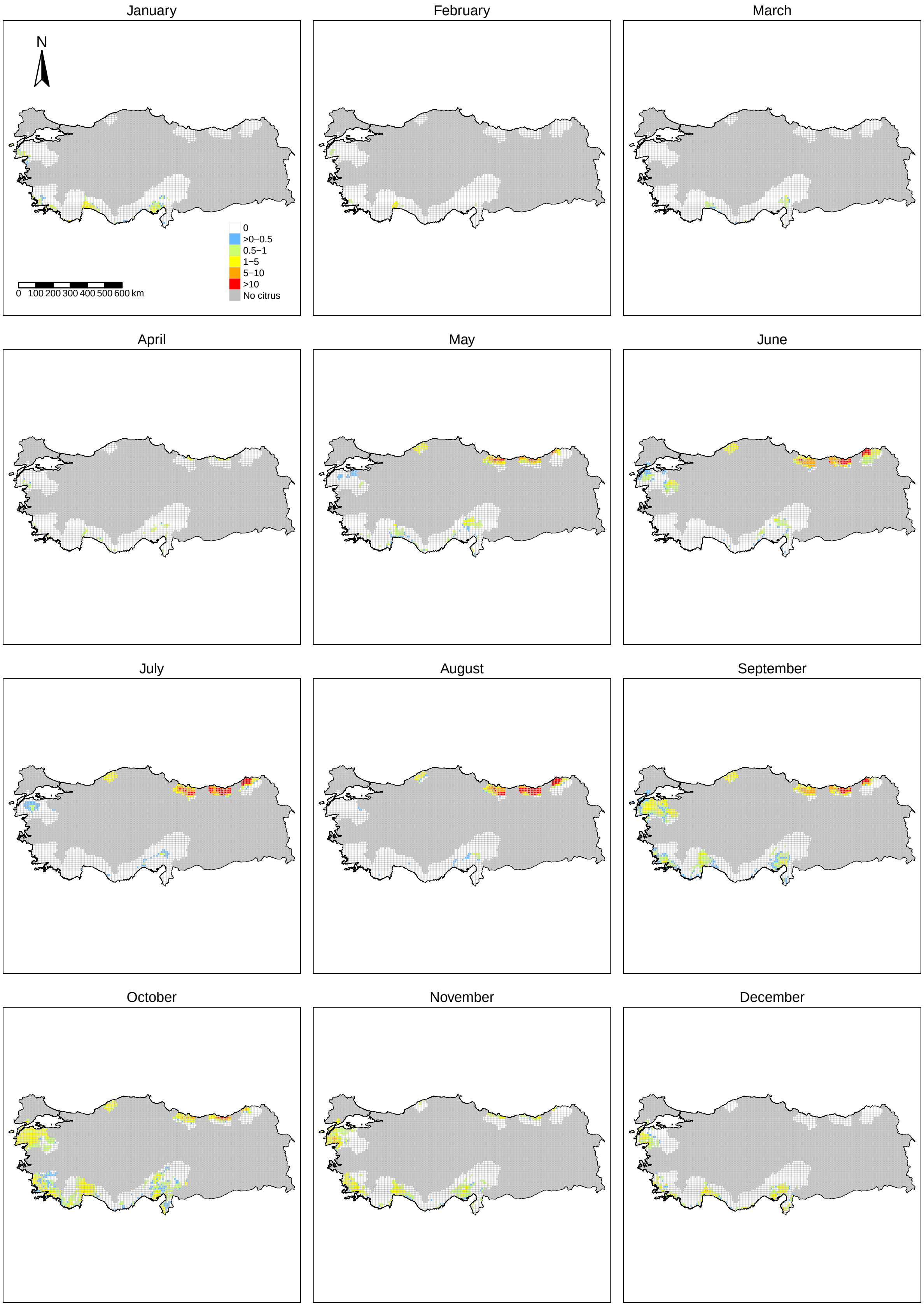

Figure SD18. Monthly percentage of hours ($= 0$ white, $]0 - 0.5]$ blue, $]0.5 - 1]$ green, $]1 - 5]$ yellow, $]5 - 10]$ orange, > 10 red) with suitable weather conditions for *Phyllosticta citricarpa* pycnidiospore infection (generic infection model for foliar fungal pathogens by Magarey et al.[1], configuration scenario S1) for the 9-km grid interpolated climatic data of the citrus-growing regions in Turkey from 2009 to 2018. Non citrus areas inside citrus-growing countries in dark-grey. The maps were created by the authors using the software R 3.6.0, https://www.R-project.org

References

1. Magarey, R., Sutton, T. & Thayer, C. A simple generic infection model for foliar fungal plant pathogens. *Phytopathology* **95**, 92–100 (2005).
