# Supplementary material for: Climate suitability of the Mediterranean Basin for citrus black spot disease (Phyllosticta citricarpa) based on a generic infection model
Source: Sci Rep. 2022 Nov 18;12:19876. doi: 10.1038/s41598-022-22775-z (PMC9674692; doi:10.1038/s41598-022-22775-z)
Supplement: Supplementary file 5 — Supplementary Information 5. [file 41598_2022_22775_MOESM5_ESM.pdf]

Supplementary Material E. Climate suitability of the Mediterranean Basin for citrus black spot disease (*Phyllosticta citricarpa*) based on a generic infection model. Figures of ascospore release from 2009 to 2018 dates by country

Anaïs Galvañ[1], Naima Boughalleb-M'Hamdi[2], Najwa Benfradj[2], Sabrine Mannai[2], Elena Lázaro[1,+], and Antonio Vicent[1,+,*]

[1]Institut Valencià d'Investigacions Agràries (IVIA), Centre de Protecció Vegetal i Biotecnologia, 46113 Moncada, Valencia, Spain
[2]Department of Biological Sciences and Plant Protection,Institut Supérieur Agronomique de Chott Mariem, LR21AGR05, University of Sousse,Chott Mariem,Sousse, 4042, Tunisia
[*]vicent_antciv@gva.es
[+]These authors contributed equally to this work

Supplementary Figures SE1 to SE6

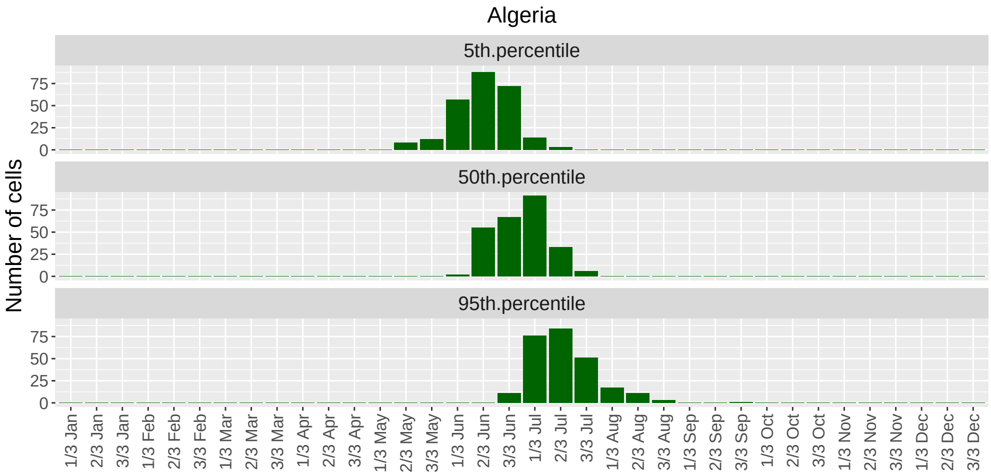

(a)

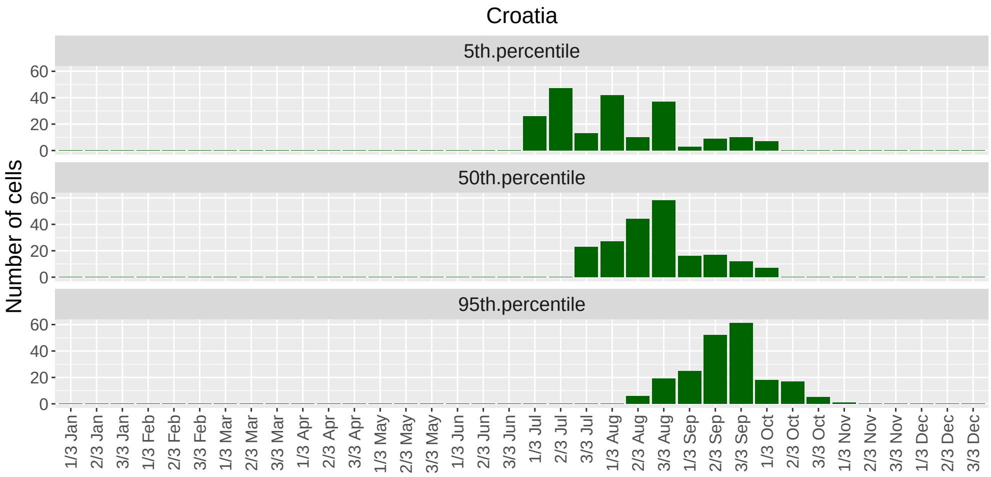

(b)

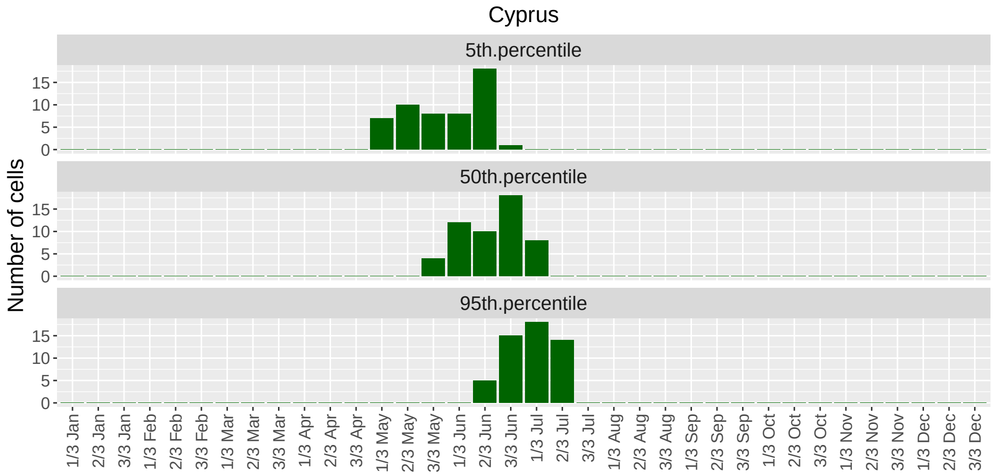

(c)

Figure SE1. Number of cells in relation to the onset of ascospore release from 2009 to 2018 (5th, 50th and 95th percentiles) predicted by the model of Moyo et al.[1] with a probability threshold of 0.5 for the 9-km grid interpolated climatic data of the citrus-growing regions in (a) Algeria, (b) Croatia, and (c) Cyprus.

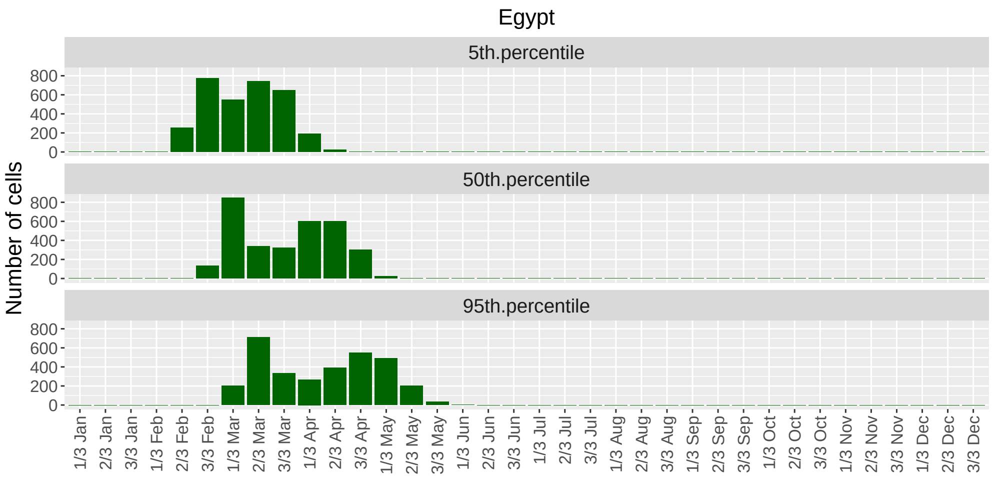

(a)

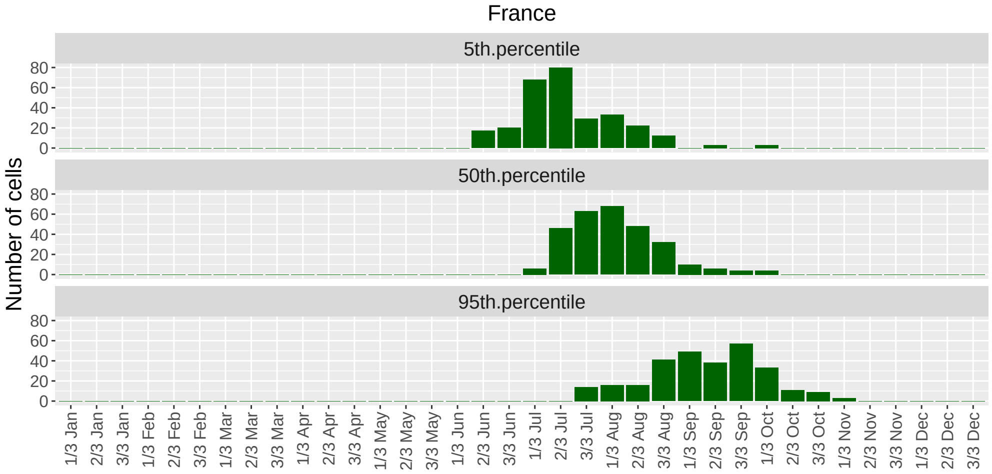

(b)

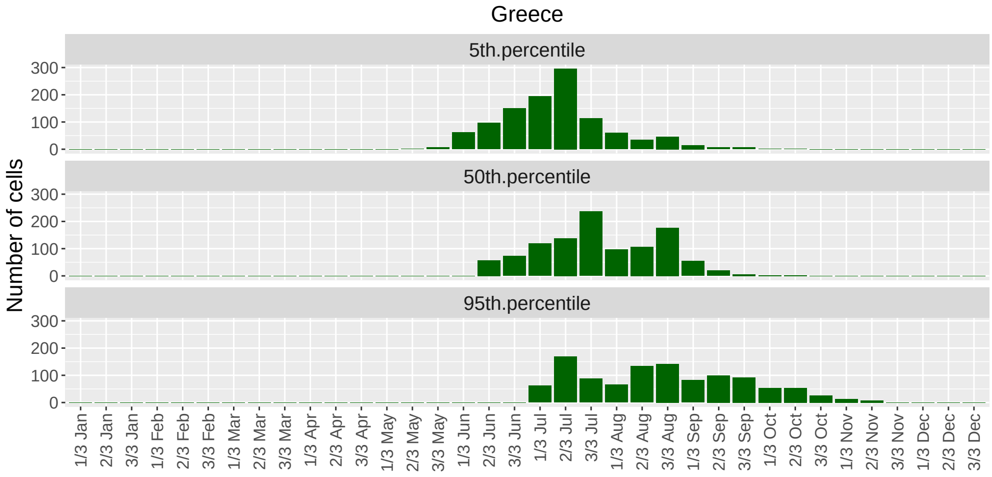

(c)

Figure SE2. Number of cells in relation to the onset of ascospore release from 2009 to 2018 (5th, 50th and 95th percentiles) predicted by the model of Moyo et al.[1] with a probability threshold of 0.5 for the 9-km grid interpolated climatic data of the citrus-growing regions in (a) Egypt, (b) France, and (c) Greece.

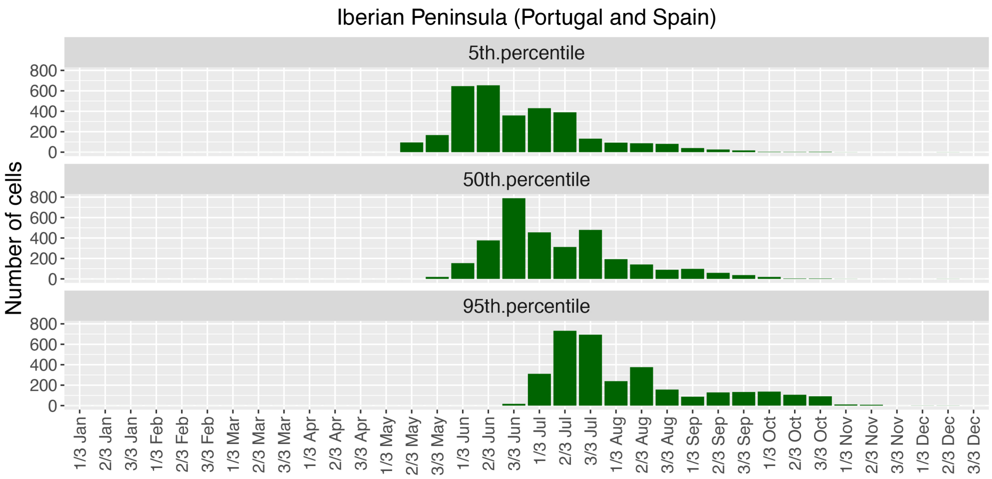

(a)

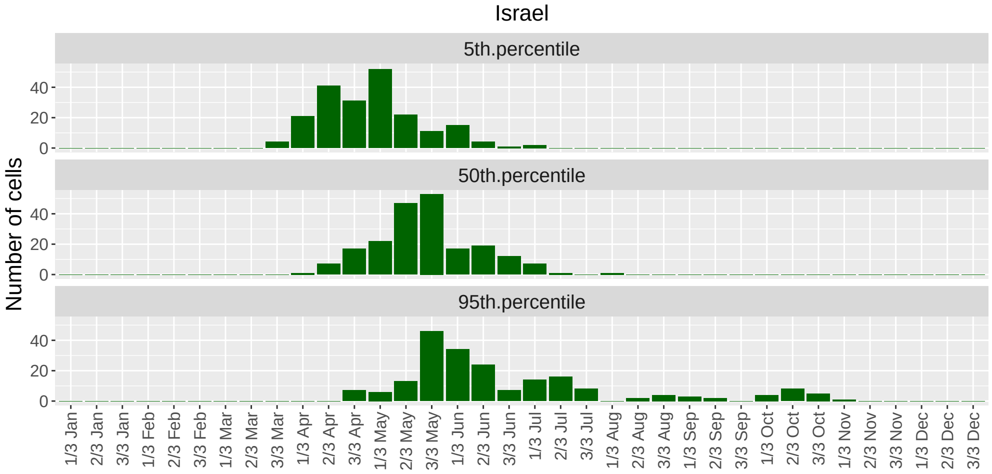

(b)

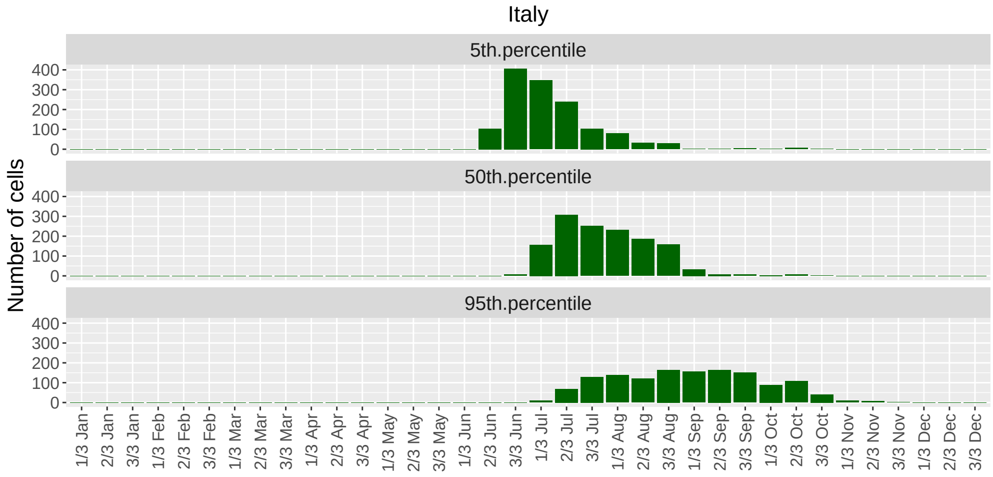

(c)

Figure SE3. Number of cells in relation to the onset of ascospore release from 2009 to 2018 (5th, 50th and 95th percentiles) predicted by the model of Moyo et al.[1] with a probability threshold of 0.5 for the 9-km grid interpolated climatic data of the citrus-growing regions in (a) Portugal and Spain, (b) Israel, and (c) Italy.

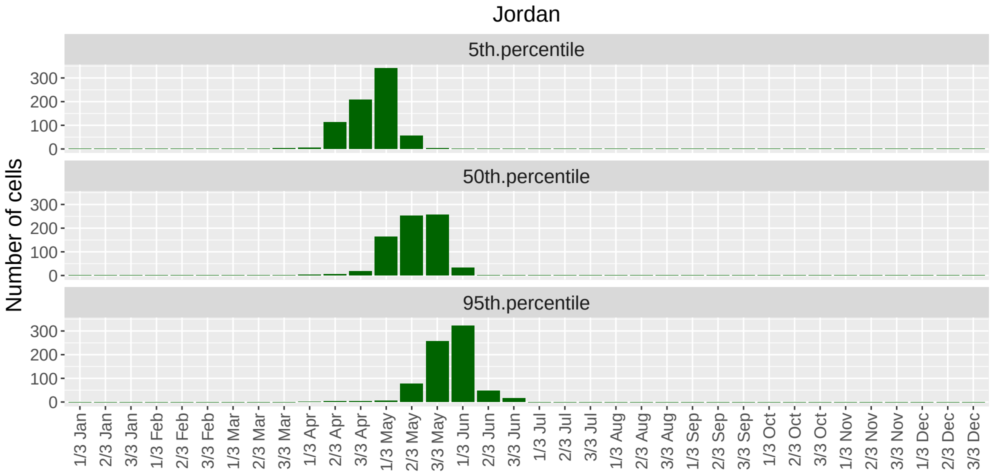

(a)

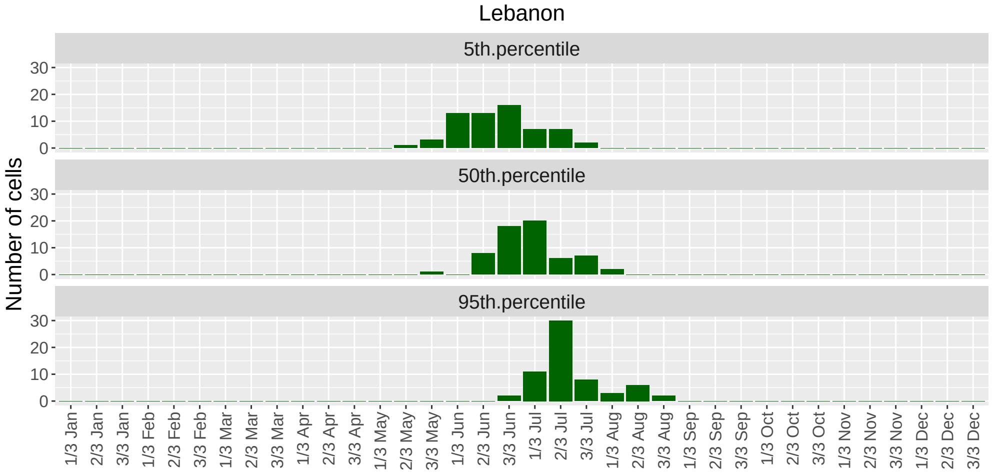

(b)

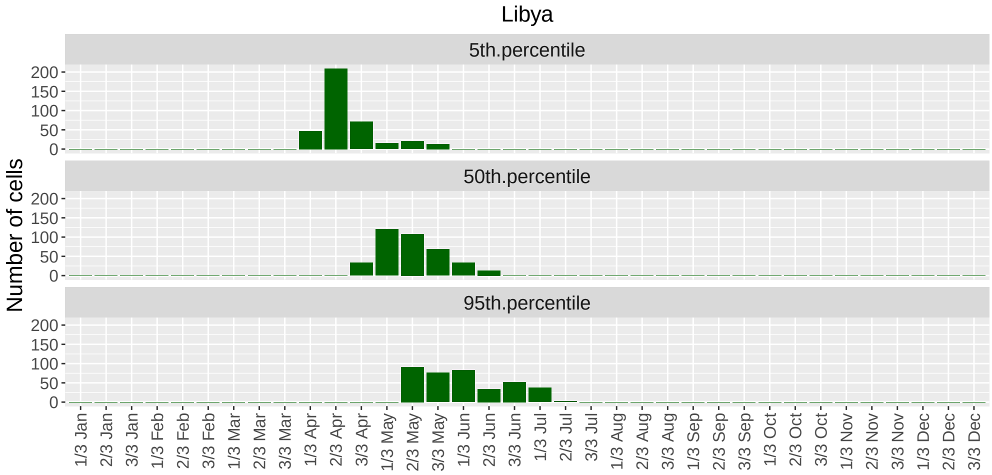

(c)

Figure SE4. Number of cells in relation to the onset of ascospore release from 2009 to 2018 (5th, 50th and 95th percentiles) predicted by the model of Moyo et al.[1] with a probability threshold of 0.5 for the 9-km grid interpolated climatic data of the citrus-growing regions in (a) Jordan, (b) Lebanon, and (c) Libya.

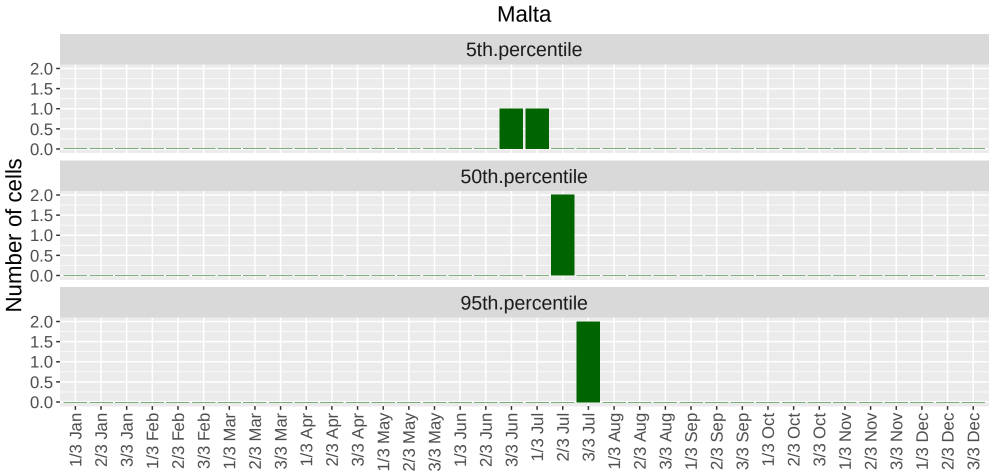

(a)

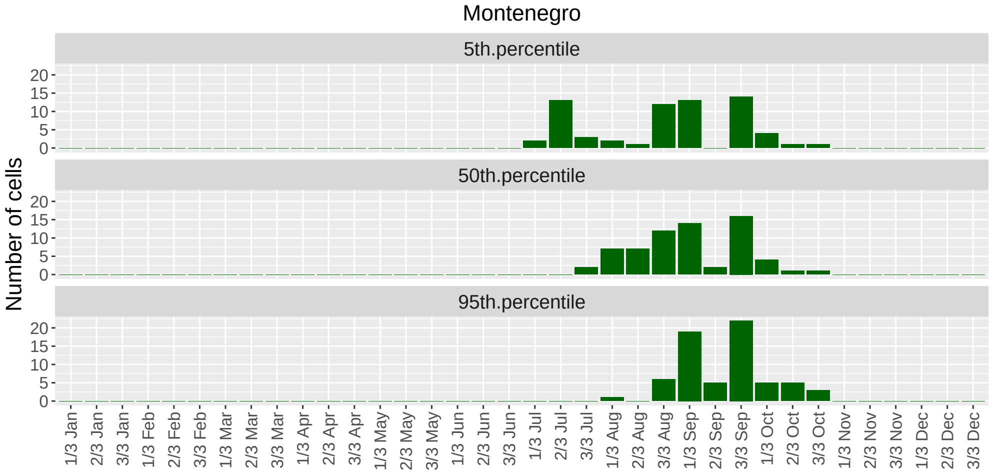

(b)

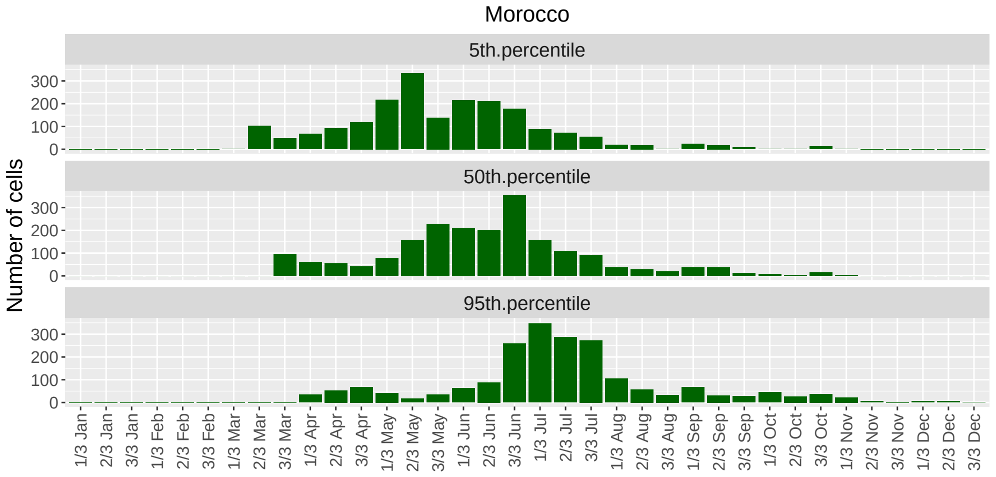

(c)

Figure SE5. Number of cells in relation to the onset of ascospore release from 2009 to 2018 (5th, 50th and 95th percentiles) predicted by the model of Moyo et al.[1] with a probability threshold of 0.5 for the 9-km grid interpolated climatic data of the citrus-growing regions in (a) Malta, (b) Montenegro, and (c) Morocco.

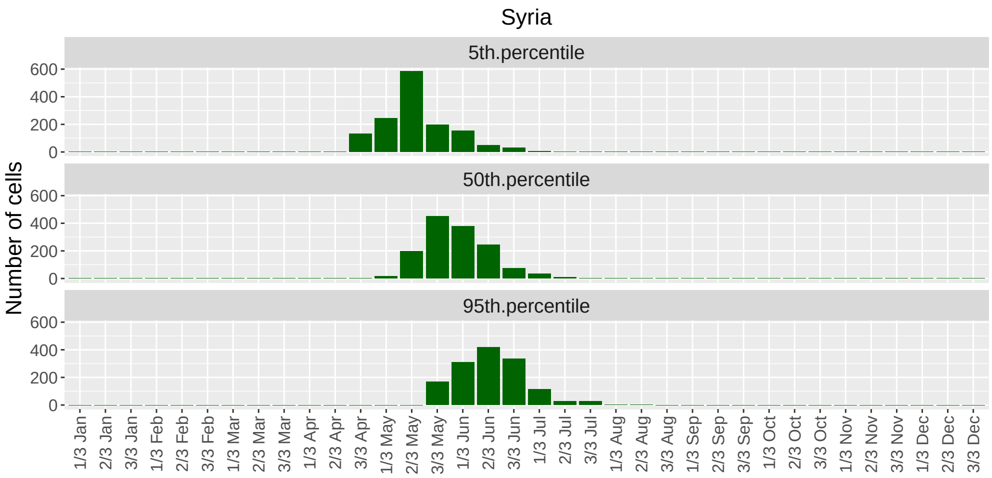

(a)

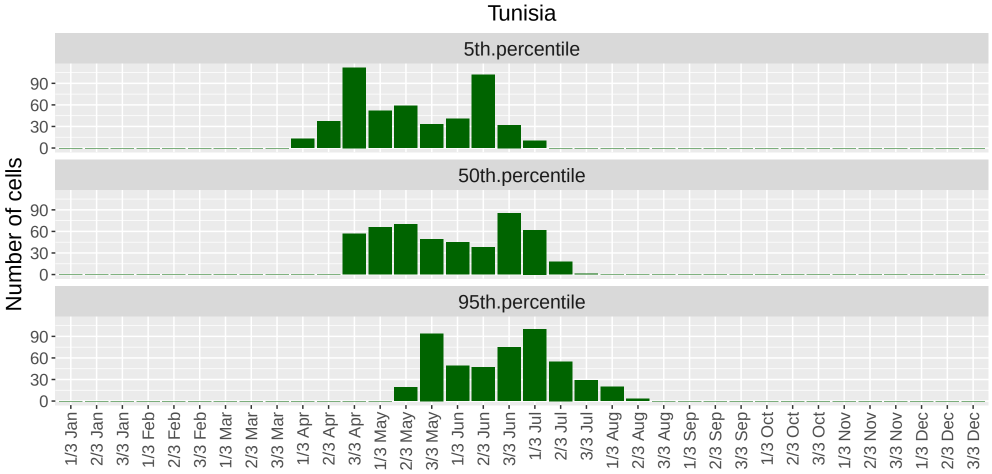

(b)

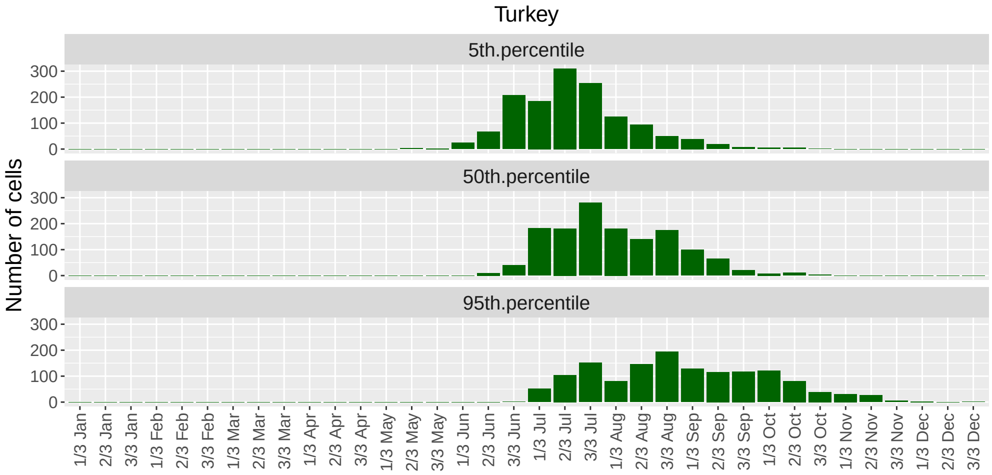

(c)

Figure SE6. Number of cells in relation to the onset of ascospore release from 2009 to 2018 (5th, 50th and 95th percentiles) predicted by the model of Moyo et al.[1] with a probability threshold of 0.5 for the 9-km grid interpolated climatic data of the citrus-growing regions in (a) Syria, (b) Tunisia, and (c) Turkey.

References

1. Moyo, P., du Raan, S. & Fourie, P. H. Models for predicting pseudothecium maturity and ascospore release of phyllosticta spp. in south african citrus orchards. *South Afr. J. Sci.* **116**, 1–10 (2020).
